# Genetic Code Expansion Facilitates Position‐Selective Labeling of RNA for Biophysical Studies

**DOI:** 10.1002/chem.201904623

**Published:** 2020-01-21

**Authors:** Andreas Hegelein, Diana Müller, Sylvester Größl, Michael Göbel, Martin Hengesbach, Harald Schwalbe

**Affiliations:** ^1^ Institute for Organic Chemistry and Chemical Biology Center for Biomolecular Magnetic Resonance Goethe University Frankfurt Max-von-Laue-Strasse 7 60438 Frankfurt am Main Germany; ^2^ Institute for Organic Chemistry and Chemical Biology Goethe University Frankfurt Max-von-Laue-Strasse 7 60438 Frankfurt am Main Germany

**Keywords:** fluorescence, genetic code expansion, NMR, RNA, site-specific labeling

## Abstract

Nature relies on reading and synthesizing the genetic code with high fidelity. Nucleic acid building blocks that are orthogonal to the canonical A‐T and G‐C base‐pairs are therefore uniquely suitable to facilitate position‐specific labeling of nucleic acids. Here, we employ the orthogonal kappa‐xanthosine‐base‐pair for *in vitro* transcription of labeled RNA. We devised an improved synthetic route to obtain the phosphoramidite of the deoxy‐version of the kappa nucleoside in solid phase synthesis. From this DNA template, we demonstrate the reliable incorporation of xanthosine during *in vitro* transcription. Using NMR spectroscopy, we show that xanthosine introduces only minor structural changes in an RNA helix. We furthermore synthesized a clickable 7‐deaza‐xanthosine, which allows to site‐specifically modify transcribed RNA molecules with fluorophores or other labels.

## Graphical abstract

**Orthogonal nucleic acid building blocks** to the canonical A‐T and G‐C base‐pairs are uniquely suitable to facilitate position‐specific labeling. Here, the orthogonal kappa‐xanthosine‐base‐pair for *in vitro* transcription is employed. Through NMR spectroscopy, it is shown that xanthosine introduces only minor structural changes. A clickable 7‐deaza‐xanthosine, which allows to site‐specifically modify transcribed RNA, is also synthesized.

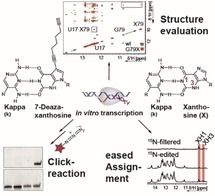

## Introduction

Over the last decade, an increasing number of functional roles has been identified for ribonucleic acids and their biophysical investigation is increasingly pursued. For a number of reasons, methods developed for proteins cannot easily be transferred for studies of RNAs. Thus, novel methods have to be developed, for example to attach labels that allow for detailed spectroscopic studies of RNA. Structural studies additionally require labels to be inserted in a site‐selective manner to obtain position‐specific readouts.

By chemical solid‐phase synthesis, RNA with such position‐specific label can be obtained. A variety of chemically modifiable building blocks has been made available over the last years. However, the use of solid phase synthesis brings along the well‐known limitations and drawbacks of this technique; most importantly the limited product yield that decreases with the length of the RNA and the concomitant increase of by‐products make purification of the target RNA difficult.

Several strategies to circumvent these limitations have been devised and successfully established in recent years. Many of these techniques use the standard approach of *in vitro* transcription to generate RNA, and employ an additional base pair that is orthogonal to the two Watson–Crick‐like base‐pairs that establish sequence specificity during transcription.^1^, ^2^, ^3^, ^4^

Two main strategies have been previously reported: either larger hydrophobic moieties establish an entirely novel complementary system or the hydrogen bonding pattern of the standard purine and pyrimidine‐based scaffolds are expanded. Both these strategies have generated additional, orthogonal base‐pairs that can be employed to different degrees in *in vitro* applications or for genetic code expansion *in vivo*. Some of the designed bases have also been elegantly used for the incorporation of modifiers, that is, for nitroxide spin labels or fluorophores.^9^, ^10^, ^11^

Our focus for the current study was to use the capabilities of a labeling strategy employing one orthogonal DNA nucleotide together with the complementary ribonucleoside triphosphate to generate site‐specifically modified RNA by *in vitro* transcription.

To introduce only minimal structural perturbation of the target RNA, we therefore opted to follow the strategy of modifying the hydrogen bond arrangement of the near canonical kappa nucleobase, which would also be sterically similar to the natural nucleobases and provide comparable stacking interfaces. Based on the work initiated by Piccirilli et al.,^4^ we first developed a novel synthesis route for the kappa nucleoside, and incorporated the kappa nucleoside phosphoramidite into a DNA used as a template for RNA transcription. We successfully incorporated xanthosine at this position with high specificity and good efficiency. We introduced xanthosine into a sizeable RNA riboswitch and could site‐specifically detect the introduced NMR‐active label. We furthermore implemented novel covalent labeling options by attaching a modifier to the xanthosine‐based nucleobase, focusing on a click chemistry compatible derivative.

## Results and Discussion

The steps required here to obtain a site‐specifically labeled RNA can be described as follows: (i) Synthesis of the kappa base (and DNA synthesis), (ii) transcription, (iii) quality control, and (iv) labeling.

### Synthesis of kappa base phosphoramidite

**Figure 1:**
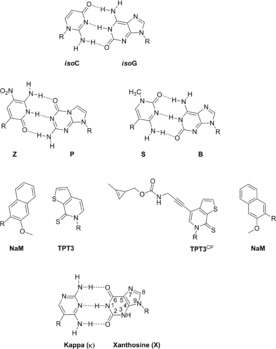
Examples of unnatural base pairs. Top: The isoC‐isoG base pair which was the first developed unnatural base pair.^5^ **Z** and **P** as well as **S** and **B** are two unnatural base pairs which were developed by Benner et al.^6^, ^7^ Middle: Hydrophobic base pairs (**NaM** and **TPT3**) which was developed by Romesberg et al.^2^ and the TPT3^CP^ and NaM base pair which was developed by Kath‐Schorr et al.^8^ Bottom: The kappa (**κ**) xanthosine (**X**) base pair used in this paper.

**Figure 2:**
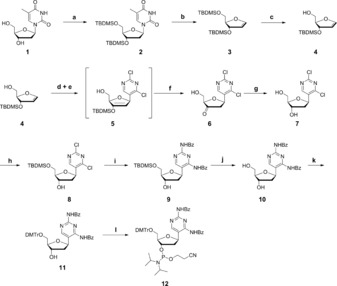
Synthetic route to the benzoyl protected 2,4‐diaminopyrimidine phosphoramidite **12**. Novel synthesis options were implemented for compounds **6**, which followed a Heck reaction, and compound **9**, where we opted for the Pd‐catalyzed Buchwald‐Hartwig coupling. Reaction conditions: a. imidazole, TBDMS‐Cl, DMF, RT, 16 h, 99 %; b. HMDS, (NH_4_)_2_SO_4_, 140 °C, 3,5 h, 88 %; c. 1 m TBAF in THF solution, THF, 0 °C, 2 h, 45 %; d. 2,4‐dichloro‐5‐iodopyrimidine, Pd(OAc)_2_, P(PhF_5_)_3_, Ag_2_CO_3_, CHCl_3_, 70 °C, 10 h; e. 1.0 m TBAF in THF solution, AcOH, THF, 0 °C, 2 h, 37 % over two steps; f. sodium triacetoxyborohydride, AcOH, MeCN, 0 °C, 15 min, 70 %; g. imidazole, TBDMS‐Cl, DMF, RT, 16 h, 76 %; h. Pd_2_(dba)_3_, xantphos, Cs_2_CO_3_, 100 °C, 24 h, 37 %; i. NEt_3_ **⋅**3 HF, THF, RT, 2,5 h, 62 %; j. DMTr‐Cl, DMAP, pyridine, RT, 12 h, 77 %; k. *N*,*N*‐diisopropylethylamine, 2‐cyanoethyl *N*,*N*‐diisopropylchlorophosphoramidite, DCM, RT, 2 h, 74 %.

Our synthetic strategy to obtain the kappa deoxynucleoside (Figure 1) incorporated a number of novel synthetic developments. Most importantly, we opted to synthesize the C−C glycosidic bond using a Heck coupling, which required generation of the glycal intermediate **4** (Figure 2).^12^, ^13^ In addition, formation of the protected amine ring substituents **9** was achieved using a palladium‐catalyzed Buchwald–Hartwig coupling.^14^

To this end, we started with deoxythymidine **1**, which was protected using *tert*‐butyldimethylsilyl chloride (TBDMS‐Cl) to yield **2**. The elimination of the nucleobase to obtain the double silylated glycal **3** turned out to be experimentally demanding due to the instability of **3** and the required purification. The following deprotection was achieved with 1.0 m tetrabutylammonium fluoride (TBAF) in THF solution. Here, both reaction monitoring and column purification had to be extensively optimized to reach a yield of 88 %. We assume that selectivity of the deprotection of C5 was limited, the yield of **4** therefore could not be optimized beyond 45 %; we could, however, not fully characterize the side products. As the double silylated glycal presumably cannot undergo the following Heck reaction,^15^ it is necessary to block the bottom of the glycal **4** by the sterically demanding TBDMS‐group to avoid the attack from this side which would result in the formation of the wrong α‐anomer.

One of the key reactions of our Scheme was the palladium‐catalyzed Heck reaction. The Heck reaction was applied to nucleic acid chemistry by Hocek et al. and Wagenknecht et al.^14^, ^15^, ^16^, ^17^ To perform this reaction, the commercially available 2,4‐dichloro‐5‐iodopyrimidine as the heterocyclic halide and the glycal **4** which acted as a cyclic alkene are coupled to yield the protected nucleoside **5**. The β‐anomer was obtained as a single product.

However, as compound **5** was unstable, we opted for an *in situ* deprotection with 1 m TBAF to yield the ketoriboside **6** with 37 %. Reduction of the ketone to the deoxyribose **7** was achieved using sodium triacetoxyborohydride (62 % yield). The Buchwald‐Hartwig coupling to obtain the benzoyl‐protected pyrimidine diamine, which was first applied by Wagenknecht et al.^14^ shows increased selectivity when the C5 of the ribose is silylated. We therefore coupled a TBDMS protection group yielding compound **8**. Here the selectivity was high due to the slightly more reactive C5′‐hydroxyl group, and the yield was 76 %. The Buchwald‐Hartwig coupling is catalyzed by palladium, and we increased the amount of benzamide to three equivalents to obtain the double protected compound **9** in sufficient yield (37 %). However, purification of **9** at this point proved difficult due to the slow migration on the column and the poor separation from the starting benzamide. Removal of TBDMS group using trimethylamine trihydrofluoride yielded compound **10** in 62 %. From here, the protocol for protection to form the phosphoramidite suitable for solid phase synthesis followed standard procedures: The DMTr protection of C5 to form **11** was achieved by DMAP‐catalyzed coupling of 4,4′‐dimethoxytritylchloride in dry pyridine. Formation of the phosphoramidite **12** was performed with 2‐cyanoethyl *N*,*N*‐diisopropylethylamine in dry DCM.

As synthesis of the longer DNA especially for transcription of a riboswitch RNA with more than 80 nucleotides is demanding, we purchased DNA from IBA (Göttingen), providing phosphoramidite **12** to be site‐specifically incorporated into the desired transcription templates.

### Synthesis of functionalized 7‐deazaxanthosine

Several options are available to provide a modifiable xanthosine derivative that maintains its Watson–Crick‐face base‐pairing interactions. Introduction of chemical modifications at C8 would be the most immediate choice. However, introduction of a sterically demanding modification at C8 introduces steric clashes in the major groove of an A‐form RNA helix.^18^ Thus, attachment of modifications at N7 promises to be structurally less perturbing, but their syntheses are difficult. However, several nucleoside derivatives have been reported that introduce a 7‐deazapurine backbone and a number of synthetic strategies^19^, ^20^, ^21^, ^22^ as well as suitable starting compounds are available. Major advantages of this approach are the conservation of the hydrogen bonding pattern at the Watson–Crick side, the advantageous substrate properties of such modified nucleotides for RNA polymerases^23^ as well as the possibility to specifically couple extended linkers using cross‐coupling reactions.

**Figure 3:**
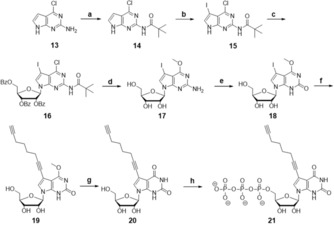
Synthetic route to the 7‐deazaxanthosine with a C‐8 linker **21**. Attachment of the clickable linker was achieved by Sonogashira coupling to **19**, and only after that the methyl ether deprotection to **20** was performed. Reaction conditions: a. pivaloyl chloride, pyridine, RT, 1 h, 92 %; b. *N*‐iodosuccinimide, THF, RT, 1 h, 93 %, c. *N*,*O*‐Bis(trimethylsilyl) acetamide, RT, 10 min, trimethylsilyl triflate, 1‐acetyl‐2,3,5‐tribenzoyl‐1‐ribose, MeCN, 50 °C, 24 h, 61 %; d. 0,5 m sodium methoxide solution in MeOH, 80 °C, 10 h, 61 %; e. sodium nitrite in H_2_O, glacial acetic acid, H_2_O, RT, 1 h, 67 %; f. Pd(PPh_3_)_4_, copper(I)iodide, NEt_3_, 1,7‐octadiyne, DMF, RT, 24 h, 96 %; g. Trimethylsilyl chloride, sodium iodide, MeCN, RT, 1 h, 61 %; h. dist. POCl_3_, *n*(Bu_3_)N, proton sponge, Me_3_PO_4_, −7.5 °C, 5 h, TBA PPi, RT, 30 min, 0,1 m TEAB buffer, RP‐HPLC, 16 %.

The synthesis starts with the commercially available 4‐chloro‐7*H*‐pyrrolo[2,3‐*d*]pyrimidin‐2‐amine **13 (**Figure 3). **13** was protected with pivaloyl chloride to obtain product **14** in excellent yield (90 %). Without the pivaloyl group, the electrophilic substitution exclusively places the bromine or iodine at the undesired C8‐position.^24^ It was also reported^21^ that without the pivaloyl protection glycosylation under Vorbrüggen conditions failed. Iodination to **15** was achieved with NIS (93 %).^25^

Initial attempts for glycosylation were made with HMDS as the silylating agent. As a result, the glycosylation reaction yielded one single product (**16**). NMR spectra, however, showed impurities and the yield was not satisfying (<40 %). To improve both purity and yield, glycosylation was done with *N*,*O*‐bis(trimethylsilyl)acetamide (BSA). Under these conditions, the glycosylation product was pure and the yields varied between 53–61 %. After the following removal of the protecting groups with 0.5 m NaOMe at 80 °C (**17**) and deamination using NaNO_3_, the desired compound **18** was obtained.

In a previously published synthesis,^21^ the methyl ether at the C4‐position was removed with trimethylsilyl (TMS) chloride and sodium iodide. Under these conditions, it was reported that a 3:2 ratio of the 7‐deazaxanthosine without iodine and 7‐deazaxanthosin with iodine was recovered and attempts to remove the methyl ether using sodium hydroxide were unsuccessful. In our case, the terminal alkyne linker is sufficiently stable during Sonogashira coupling conditions. We therefore directly conducted the Sonogashira coupling of the 1,7‐octadiyne linker to obtain **19**. We used 10 equivalents of 1,7‐octadiyne and a 2:1 ratio of copper/palladium.^26^ The methyl protection group at O6 was only then removed using TMS chloride and sodium iodide at room temperature with a 61 % yield of **20**.

Triphosphorylation of the modified xanthosine derivative is necessary to make it amenable for T7 RNA polymerase‐based transcription. Therefore, the final step is the coupling of the triphosphate specifically to C5 of the ribose. Here, protecting strategies have been used previously.^27^ We found that the conditions used by Yoshikawa and Ludwig^28^, ^29^, ^30^ were compatible with our modification, and provided the advantage that protection of the other two ribose hydroxyl moieties was not required to obtain the desired specificity for C5. We adapted the protocol and extended the hydrolysis step with 0,1 m TEAB buffer to eight hours. After purification by RP‐HPLC, the final nucleoside triphosphate **21** was obtained in 16 % yield. With this, both orthogonal base‐pairing building blocks were ready to be tested in transcription and labeling.

### Site‐specific incorporation of xanthosine by *in vitro* transcription

We first tested incorporation of a xanthosine (X) to be decoded by a DNA nucleotide containing the kappa nucleobase by standard T7 run‐off transcription. The kappa nucleobase was incorporated into two DNA‐templates, encoding a 14mer RNA hairpin with a UUCG tetraloop motif, and the aptamer domain of the guanine‐riboswitch‐aptamer RNA (Gsw^73^) from *Bacillus subtilis*. Run‐off transcription can utilize single‐stranded DNA where only the T7 primer region of the template DNA is double stranded. Using this strategy, it is sufficient to synthesize the kappa DNA phosphoramidite.^31^

**Figure 4:**
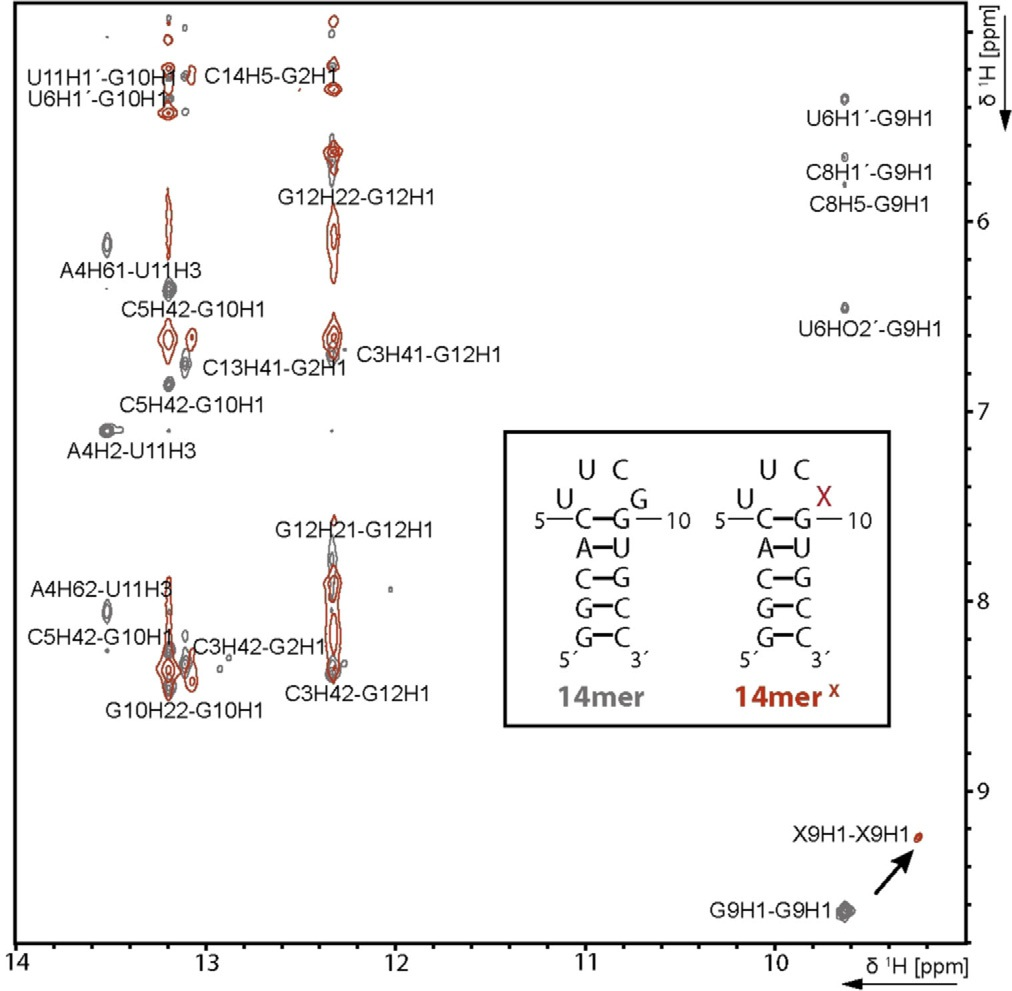
Overlaid imino‐amino‐ and imino‐sugar region of ^1^H, ^1^H‐NOESY spectra of the 14mer (grey) and 14mer^X^ (red) RNA displayed on the left. The assignment of the 14mer has been transferred from Fürtig et al.^32^ The signals of the G9X modification are significantly shifted and no crosspeaks can be detected for the X9, whereas the cross signals of G2, G10 and G12 are mostly retained. This finding hints towards a restructuring in the loop region by conservation of the hairpin structure. Experimental condition: 1 mm RNA in 25 mm potassium phosphate buffer pH 6.2 and 10 % D_2_O were measured at 278 K and 600 MHz.

As a proof of concept, a G9X mutation was introduced into the 14mer UUCG‐tetraloop hairpin RNA, as guanosine has high structural similarity to xanthosine. Transcription yielded a highly pure RNA. The yield upon switching from G to X dropped by 87 % (Figure S1, Table S1). We further investigated the impact of introducing xanthosine on RNA structure using NMR spectroscopy. 1D ^1^H NMR showed that folding into the hairpin structure is retained and introduction of xanthosine leads to only small structural deviations (Figure S2). The UUCG tetraloop is usually highly ordered^33^ and was destabilized by the mutation which is visible in both 1D ^1^H and ^1^H, ^1^H‐NOESY NMR spectra (Figure 4 and Figure S2). These results show that preparative transcription using the modified constructs is feasible, and that replacing a guanosine with a xanthosine yields an interesting atomic mutagenesis approach to investigate local structural effects by NMR.

**Figure 5:**
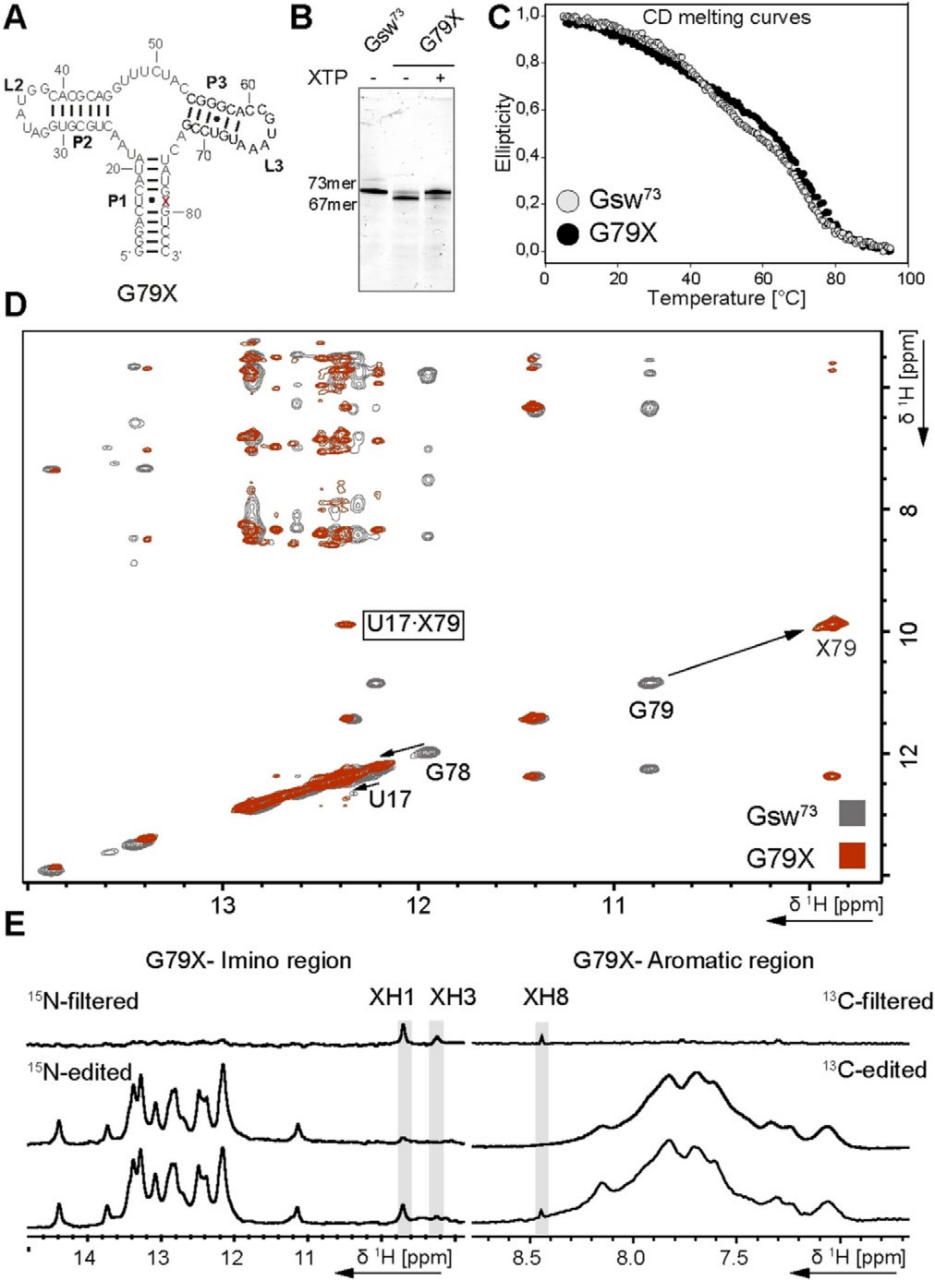
**A** secondary structure of G79X **B** denaturing PAA‐Gel for comparison of the transcription efficiency of unmutated Gsw^73^ versus G79X in absence and presence of XTP. **C** Overlay of CD melting curves recorded with Gsw^73^ (grey) and G79X (black) showing minimal changes in stability of 0.25 K. **D** Overlay of the imino‐imino‐ and imino‐cross peak region of ^1^H, ^1^H‐NOESY spectra of Gsw^73^ (grey) and G79X (red). Again, a strong shift for the iminos of the mutated position and minor shifts for the imino signals of the nearby nucleotides can be observed. Here, the cross signals for the X79 are detectable showing complete structural conservation. Experimental condition: G79X (100 μm) was recorded at 950 MHz in 25 mm potassium phosphate pH 6.2 containing 50 mm KCl and 10 % D_2_O Gsw^73^ was recorded by Janina Buck (unpublished data) in the same buffer at 900 MHz. The partial assignment has been transferred from Buck et al.^36^ **E** Imino and aromatic region of ^13^C, ^15^N A,C,G,U‐labeled G79X. Bottom: spectra without *x*‐filter, middle: ^15^N‐/^13^C‐edited spectra, only hydrogens bound to ^15^N or ^13^C are visible, top: ^15^N‐/^13^C‐filtered spectra, only hydrogens bound to the unlabeled xanthosine are visible. Experimental condition: The RNA was in a 25 mm potassium phosphate pH 6.2 containing 50 mm KCl and 10 % D_2_O for the imino spectra and 100 % D_2_O for the spectra of the aromatic region. Spectra were recorded at 600 MHz.

We then turned to a large structured RNA to test whether these findings also hold true for the analysis of functional/biologically relevant RNA. Here, we used the guanine sensing riboswitch RNA from *B. subtilis*.^34^, ^35^, ^36^, ^37^ For this construct, we replaced G79 forming a G⋅U wobble base pair in the P1 stem with X79 (Figure 5 A). We optimized the transcription conditions with regard to the concentration of XTP (Figure S3). Under optimized conditions, the yield of the full‐length G79X transcript was 71.4 % in comparison with transcription of the unmodified Gsw^73^‐transcript. Not surprisingly, we observed abortion products at the site of the mutation, with a ratio of 77:23 full‐length RNA to abortion fragment. In absence of the xanthosine triphosphate, we also observed 22 % full‐length product (Figure 5 B, Table S2), which presumably arises from misincorporation of adenosine.^4^

CD melting experiments showed a small difference of 0.25 K in the melting points (Δ*T*_m_) between the Gsw^73^ and G79X riboswitch, indicating that the structure is retained (Figure 5 C). We further purified the full‐length G79X RNA and obtained about 22 nmol of RNA, which was sufficient for NMR studies. The NMR data fully supported our proposed strategy that introduction of xanthosine induces minimal structural perturbation. Furthermore, the previous G79 H1′ cross peaks are now visible as X79 H1′ cross peaks, indicating that the structure is retained (Figure 5 D).

We prepared a reversed labeled riboswitch with ^13^C, ^15^N‐labeled ATP, CTP, GTP and UTP together with unlabeled XTP. With this isotopically labeled RNA sample, we performed ^13^C and ^15^N‐filtered 1D ^1^H‐NMR experiments that suppress signals from hydrogen atoms bound to ^13^C and ^15^N. In this experimental setup, we detected the single ^12^C,^14^N‐“labeled” xanthosine, in particular the signals annotated XH1 and XH3 imino‐ and the XH8 aromatic signal in the background of the other 73 nucleotides of the riboswitch RNA (Figure 5 E). Thus, transcribing RNA from a ssDNA template that contains the kappa nucleotide can yield large functional RNAs, as both selectivity and specificity are very high. Purification yields a highly homogenous RNA sample suitable for NMR spectroscopy.

### Post‐transcriptional functionalization of RNA via click reaction

Site‐specific introduction of a non‐natural nucleobase can be exploited for labeling purposes.^4^ We therefore used the 7‐deazaxanthosine (7dX) derivative containing a terminal alkyne in the form of a triphosphate during *in vitro* transcription to introduce a position‐selective spectroscopic label.

**Figure 6:**
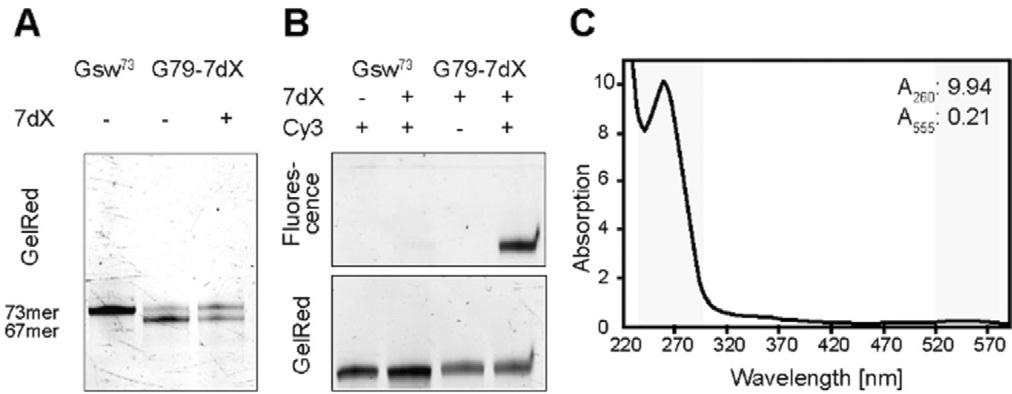
**A** denaturing PAA‐Gel for comparison of the transcription efficiency of unmutated Gsw^73^ versus G79‐7dX in absence and presence of 7‐deazaxanthosinetriphosphate (7dX). **B** Fluorescence scan (upper gel) and gel red staining (lower gel) of denaturing PAA‐Gel after Click‐reaction. **C** UV‐spectrum of fluorophore‐labeled RNA after further gel purification to determine the yield of labeling reaction.

Transcription of the kappa‐modified Gsw^73^ DNA‐template with the alkyne‐XTP **13** resulted in a 1:1 ratio of full‐length G79‐7dX to abortion fragment (67 nt), with a total yield of 34.5 % full‐length RNA compared to the unmodified riboswitch (Figure 6 A, Figure S4, Table S3). As expected, the rate of misincorporation in absence of 7dXTP remains comparable to the misincorporation observed in absence of XTP under the respective optimized conditions for both transcripts. The modified 73mer RNA was purified by extraction from a polyacrylamide gel. For labeling, a click reaction with Cy3‐Azide was performed, and the resulting RNA analyzed by PAGE. The signals shown in the gel lanes for RNA staining showed that the RNA remained intact during labeling, and no significant shift was observed. For RNA that contained the alkyne‐modified xanthosine, a fluorescent signal could be observed comigrating with the unlabeled RNA, demonstrating a highly specific labeling (Figure 6 B). The fluorescently labeled RNA was further excised from the gel, and analyzed using UV/Vis spectroscopy. Based on the intensities at the absorption maxima (DNA: 260 nm, Cy3: 555 nm), we calculated the labeling yield of the RNA to be 11 % (Figure 6 C).

## Conclusion

We prepared two well‐structured RNAs—a model 14mer as well as a 73 nt aptamer of a riboswitch—via run‐off transcription and introduced a single guanosine‐to‐xanthosine mutation, in each case yielding a 100 μm NMR sample. We show that the structural perturbations introduced into two different RNA constructs by the unmodified xanthosine are minor. We furthermore devised a modifiable 7‐deazaxanthosine triphosphate carrying a modifiable linker for posttranscriptional attachments of spectroscopic labels for example, for FRET‐, EPR‐ or IR‐measurements. Due to the demanding synthesis of this compound, the final triphosphorylation currently limits the obtainable yield, motivating the urgent need for novel synthetic routes for triphosphorylation.^38^ We show that our approach facilitates labeling of biologically relevant RNAs (i.e. riboswitches) in a position‐selective manner. With the synthesis of both the kappa DNA phosphoramidite and a modifiable RNA‐xanthosinetriphosphate derivative, we provide a proof of concept to employ non‐natural, Watson–Crick‐like base pairs for site‐specific bioorthogonal labeling of RNA.

## Experimental Section

### Solid‐phase synthesis of the kappa (κ)‐containing DNA template

The phosphoramidite containing the κ‐nucleobase has been incorporated into the DNA via solid‐phase synthesis by commercial DNA synthesis (IBA Lifesciences; Göttingen, Germany).

### Expression and purification of P266L T7 RNA Polymerase

The protein was expressed in BL21(DE3)cells carrying a polyhistidine (His_6_‐Tag). Expression was induced at OD600=0.6–0.8 with 0.5 mm isopropyl‐d‐1‐thiogalactopyranoside (IPTG). T7 RNA Polymerase was expressed over night at 37 °C and were purified via HisTrap HP(GE Healthcare) columns using 50 mm Tris/HCl (pH 8.1), 400 mm NaCl, 20 mm imidazole and 5 mm β‐mercaptoethanol as lysis buffer. The protein was further purified via size exclusion chromatography with a buffer containing 20 mm sodium phosphate (pH 7.7) 150 mm NaCl, 1 mm EDTA and 5 mm DTT.

### DNA templates

14mer‐template (template 1): GGC ACC GAA GTG CCT ATA GTG AGT CGT ATT A

14mer: GGCACUUCGGUGCC

14mer^X^‐ template (template 2): GGC ACκ GAA GTG CCT ATA GTG AGT CGT ATT A

14mer: GGCACUUCXGUGCC

Gsw^73^‐template (template 3): GGG ACC CAT AGT CGG ACA TTT ACG GTG CCC GGT AGA AAC CTG CGT GCC ATA TCC ACG CAG TTA TAT GAG TCC CTA TAG TGA GTC GTA TTA

Gsw^73^: GGG ACU CAU AUA ACU GCG UGG AUA UGG CAC GCA GGU UUC UAC CGG GCA CCG UAA AUG UCC GAC UAU GGG UCC C

G79X‐template (template 4): GGG ACκ CAT AGT CGG ACA TTT ACG GTG CCC GGT AGA AAC CTG CGT GCC ATA TCC ACG CAG TTA TAT GAG TCC CTA TAG TGA GTC GTA TTA

G79X: GGG ACU CAU AUA ACU GCG UGG AUA UGG CAC GCA GGU UUC UAC CGG GCA CCG UAA AUG UCC GAC UAU GXG UCC C

G79‐7dX: GGG ACU CAU AUA ACU GCG UGG AUA UGG CAC GCA GGU UUC UAC CGG GCA CCG UAA AUG UCC GAC UAU G7dXG UCC C

The underlined regions correspond to the inverse complementary T7 promoter sequence.

### *In vitro* transcription

40 mm tris glutamate (pH 8.1), 2 mm spermidine, 20 mm DTT and 10 ng μL^−1^ P266L T7 RNA polymerase were used for all the transcription reactions. The other reaction components have been optimized for the 14mer to 45 mm Mg^2+^, 1 mm NTPs (each) and 300 nm DNA template 1 and for the 14mer^X^ to 10 mm Mg^2+^, 2 mm NTPs (each), 1 mm XTP, and 200 nm DNA template 2. The transcription reactions of Gsw^73^, G79X and G79‐7dX were performed with the same concentration of tris glutamate, spermidine, DTT and RNA polymerase. Gsw^73^ was transcribed from 300 nm DNA template 3 in presence of 35 mm Mg^2+^ and 6 mm NTPs (each). For the *in vitro* transcription of G79X 200 nm DNA template 4 were used and 35 mm Mg^2+^, 6 mm NTPs (each) and 3 mm XTP (Jena Bioscience) were added. The transcription of G79‐7dX was performed in presence of 200 nm DNA template 4, 35 mm Mg^2+^, 6 mm NTPs (each), 15 % DMSO and 2.5 mm 7‐deazaxanthosine derivative. The relative yield was determined with ImageJ^39^ or ImageLab software after polyacrylamide gel electrophoresis (PAGE).

### RNA purification

The 14mer transcription reaction was purified using the protocol described by Helmling et al.^40^

The G79X/‐7dX transcription reaction was desalted with ddH_2_O using centrifuge concentrators (5 kDa MWCO cut‐off, Satorius), subsequently gel purified with a 15 % denaturing polyacrylamide gel. The desired fraction were extracted from gel by incubating in 0.3 m NaOAc over night at 37 °C. The supernatant was separated and the dissolved RNA was precipitated by addition of 2.5 V 99.5 % EtOH (Carl Roth) and storage at −20 °C for 2 h. The RNA was collected by centrifugation at 8500 g for 30 min and the pellet was dried and dissolved in an appropriate volume of ddH_2_O. For removal of PAA impurities, the RNA was HPLC purified, lyophilized, desalted using centrifuge concentrators (5 kDa MWCO cut‐off, Satorius) and first precipitated with EtOH (see above), then with 5 V 2 % LiClO_4_ in Acetone (*w*/*v*) for 3 times to remove residual salt from HPLC‐buffer. In the last step, the sample was desalted again and concentrated to 100–300 μm.

### Click‐reaction

2 μm RNA, 15X excess of Cy‐3‐azide, 50 % DMSO, 0.3 mm CuSO_4_, 2 mm TBTA and 5 mm sodium ascorbate were mixed and incubated at 20 °C for 1 h. A sample was separated using PAGE and analysis of the fluorescence signal was carried out with a Typhoon9400. After further PAGE purification of the RNA, the click efficiency could be determined via UV absorption measurement using *ϵ*_260_ of 796.5 L mmol^−1^ cm^−1^ for the RNA and *ϵ*_555_ of 150.0 L mmol^−1^ cm^−1^ for Cy3.

### CD melting analysis

16 μm RNA in 25 mm potassium phosphate buffer pH 6.2 and 50 mm KCl was provided. 400 μm Mg^2+^ was added. The melting curves were recorded between 5–95 °C and reverse at 264.2 nm. SigmaPlot 12.5 was used for the analysis. A bisigmoidal fit was used to determine the melting points.

### NMR‐spectroscopy

14mer and 14mer^X^ were measured at 278 K and 600 MHz at 1 mm RNA concentration in 25 mm potassium phosphate buffer pH 6.2 and 10 % D_2_O.

The ^1^H,^1^H‐NOESY of G79X was recorded at 950 MHz on an unlabeled sample (100 μm) in 25 mm potassium phosphate pH 6.2 containing 50 mm KCl and 10 % D_2_O. A sequence with jump‐return‐echo water suppression^41^ was used. For the *x*‐filter experiments,^42^ a ^13^C, ^15^N‐A, C, G, U labeled sample was prepared. For the imino region, jump‐return‐echo water suppression was used as well. The concentration was 160 μm in the same buffer as described above and 10 % D_2_O. The aromatic region was recorded with transfer using DIPSI2 sequence for mixing with ^13^C‐ and ^15^N‐filter^42^, ^43^, ^44^, ^45^, ^46^ and watergate sequences for water suppression.^47^ The concentration was 60 μm in the same buffer as described above in 100 % D_2_O. The spectra were recorded at 600 MHz at 308 K.

### Chemical synthesis

**Synthesis of 3′,5′‐bis‐*O*‐(*tert*‐butyldimethylsilyl) thymidine (2)**: In a round flask *tert*‐butyldimethylsilylchloride (TBDMS‐Cl) (3 equiv, 65.1 g, 0.432 mol) and imidazole (6 equiv, 59.01 g, 0.867 mol) was dissolved in 1085 mL *N*,*N*‐dimethylformamide (DMF). After that, thymidine (1 equiv, 35 g, 0.144 mol) was added. The reaction mixture was stirred over night at room temperature. The mixture was quenched with 75 mL methanol (MeOH) and was diluted with 750 mL ethyl acetate (EtOAc). The organic phase was washed with distilled water (2×600 mL), with saturated sodium bicarbonate (300 mL, NaHCO_3_) and with saturated sodium chloride (300 mL, NaCl). After that it was dried over sodium sulfate (Na_2_SO_4_). The solvent was evaporated under reduced pressure to afford a white solid. This solid was purified by column chromatography on silica gel, elution with cyclohexane (Cy)/ ethyl acetate (EE) 2:1 afforded **2** as a white solid. TLC (cyclohexane/ethylacetate 2:1): *R*_f_=0.44. Yield: 67.1 g (99 %) (Figure S5–S7). ^1^H‐NMR (500 MHz, CDCl_3_): *δ*=8.43 (br s, 1 H, NH), 7.47 (m, 1 H, H‐6), 6.33 (dd, *J=*5.7, 7.9 Hz, 1 H, H‐1′), 4.40 (m, 1 H, H‐3′), 3.93 (m, 1 H, H‐4′), 3.88–3.85 (m, 1 H, H‐5′a), 3.77–3.74 (m, 1 H, H‐5′b), 2.26–2.22 (m, 1 H, H‐2′a), 2.02–1.97 (m, 1 H, H‐2′b), 1.91 (m, 3 H, CH_3_), 0.93 (s, 9 H, C(CH_3_)_3_), 0.89 (s, 9 H, C(CH_3_)_3_), 0.11 (d, *J*=1.3 Hz, 6 H, Si(CH_3_)_2_), 0.08 (s,3 H, Si(CH_3_)_2_), 0.07 ppm (s, 3 H, Si(CH_3_)_2_). ^13^C‐NMR (125 MHz, CDCl_3_): *δ*=163.6 (C‐4), 150.3 (C‐2), 135.6 (C‐6), 110.9 (C‐5), 87.9 (C‐4′), 84.9 (C‐1′), 72.4 (C‐3′), 63.1 (C‐5′), 41.5 (C‐2′), 26.0 (*C*CH_3a_), 25.9 (*C*CH_3b_), 18.5 (C*C*H_3a_), 18.1 (C*C*H_3b_), 12.7 (CH_3_), −4.5 (SiCH_3a_), −4.7 (SiCH_3b_), −5.2 (SiCH_3c_), −5.3 ppm (SiCH_3d_). ESI‐MS: *m*/*z* calcd for C_22_H_42_N_2_O_5_Si_2_ [*M*+H^+^]^+^: 470.75; found: 471.25

**Synthesis of 1,4‐anhydro‐3,5‐bis‐*O*‐(*tert*‐butyldimethylsilyl)‐2‐deoxy‐d‐erythro‐pent‐1‐enitol (3)**: Compound **2** (1 equiv, 67.07 g, 0.142 mol) was placed into a dried Schlenk flask under an argon atmosphere. Hexamethyldisilazane (HMDS) (744 mL, 3.60 mol) and ammonium sulfate (0.25 equiv, 4.7 g, 0.036 mol) was added, and the solution was stirred until dissolved. The solution was refluxed at 140 °C for 3.5 hours. HMDS was evaporated under reduced pressure. MeOH (600 mL) was slowly added at room temperature. Potassium carbonate (K_2_CO_3_) (1.1 equiv, 21.59 g, 0.156 mol) was added in portions and stirred for 45 min at 0 °C. This solution was filtered over a pad celite and the solvent was evaporated under reduced pressure. The brown residue was purified by column chromatography on silica gel, elution with dichloromethane/ethyl acetate (EE) 1:2 afforded **2** a yellow oil. TLC (ethylacetate/dichloromethane 2:1) *R*_f_=0.91. Yield: 42.9 g (88 %) (Figure S8–S10). ^1^H‐NMR (500 MHz, CDCl_3_): *δ*=6.46 (d, *J*_H2_=2.5 Hz, 1 H, H‐1′), 5.00 (t, *J=*2.66 Hz, 1 H, H‐2′), 4.89 (m, 1 H, H‐3′), 4.28 (dt, J_H3_=2.7 Hz, *J*_H5_=6.2 Hz, 1 H, H‐4′), 3.69 (dd, *J*_H4_=5.7 Hz, *J*_H5_=10.7 Hz, 1 H, H‐5′), 3.50 (dd, *J*_H4_=6.3 Hz, *J*_H5_=10.7 Hz, 1 H, H‐5′), 0.89 (2 s, 18 H, C(CH_3_)_3_), 0.09, 0.08, 0.07, 0.06 ppm (4 s, 12 H, Si(CH_3_)_2_). ^13^C‐NMR (100 MHz, CDCl_3_): *δ*=149.1 (C‐1′), 103.5 (C‐2′), 89.0 (C‐3′), 76.1 (C‐4′), 62.9 (C‐5′), 26.0, 25.9 (C(*C*H_3_)_3_), 18.5, 18.2 (*C*(CH_3_)_3_), −4.2 (SiCH_3_), −4.3 (SiCH_3_), −5.2 (SiCH_3_), −5.3 ppm (SiCH_3_). MALDI‐MS: *m*/*z* calcd for C_17_H_36_O_3_Si_2_ [*M*+H^+^]^+^: 344.64; found: 344.91.

**Synthesis of 3‐*O*‐(*tert*‐butyldimethylsilyl)‐1,2‐dideoxy‐2,3‐didehydro‐d‐ribofuranose (4)**: To a solution of compound **3** (1 equiv, 42.89 g, 0.124 mol) in THF (445 mL) at 0 °C was added in portions a solution of TBAF 1 m in THF (1 equiv, 124.45 mL, 0.124 mol), and the reaction mixture was stirred for 2 hours. After two hours the reaction mixture was diluted with distilled water (500 mL) and with dichloromethane (400 mL). The organic phase was washed with distilled water (200 mL), brine (200 mL) and dried over sodium sulfate (Na_2_SO_4)_. The solvent was evaporated under reduced pressure. The residue was purified by column chromatography on silica gel, elution with *n*‐hexane/ethyl acetate 4:1 afforded **4** as a pale yellow oil. TLC (*n*‐hexane/ethylacetate 4:1) *R*_f=_0.39. Yield: 12.8 g (45 %) (Figure S11–S13). ^1^H‐NMR (500 MHz, [D_6_]DMSO): *δ*=6.61 (dd, *J*_H2_=2.7 Hz, *J*_H3_=0.8 Hz, 1 H, H‐1′), 5.02 (t, *J*_H1_=2.8 Hz, *J*_H3_=2.8 Hz, 1 H, H‐2′), 4.93 (t, *J*_H5_=5.8 Hz, 1 H, OH), 4.80 (td, *J*_H3_=*J*_H4_=2.7 Hz, *J*_H1_=0.93 Hz, 1 H, H‐3′), 4.11 (td, *J*_H5_=6.2 Hz, *J*_H3_=2.8 Hz, 1 H, H‐4′), 3.43 and 3.28 (2×td, *J*_gem_=11.6 Hz, *J*_H4_=6 Hz, *J*_OH_=5.8 Hz, 2×1 H, H‐5′), 0.85 (s, 9 H, C(CH_3_)_3_), 0.06 und 0.05 ppm (2×s, 2×3 H, (Si(CH_3_)_2_). ^13^C‐NMR (125 MHz, [D_6_]DMSO): *δ*=149.2 (C‐1′), 103.2 (C‐2′), 89.0 (C‐3′), 75.7 (C‐4′), 60.9 (C‐5′), 25.8 (C(*C*H_3_)_3_), 17.7 (*C*(CH_3_)_3_), −4.4 (SiCH_3_), −4.5 ppm (SiCH_3_). MALDI‐MS: *m*/*z* calcd for C_11_H_22_O_3_Si_2_ [*M*+H^+^]^+^: 230.38; found: 230.98.

**Synthesis of 1β‐(2,4‐dichloropyrimidin‐5‐yl)‐1,2,3‐trideoxy‐3‐oxo‐d‐ribofuranose (6)**: Palladium(II)acetate (0.1 equiv, 1.26 g, 0.0056 mol) and tris(pentafluorophenyl)phosphine (0.2 equiv, 5.96 g, 0.011 mol) was dissolved in a dried Schlenk flask in dry chloroform (194 mL). The mixture was stirred for one hour at room temperature under an argon atmosphere. After 1 h compound **4** (1 equiv, 12.8 g, 0.056 mol), 2,4‐dichloro‐5‐iodopyrimidine (1.2 equiv, 18.4 g, 0.067 mol) and silver carbonate (1 equiv, 15.4 g, 0.056 mol) were added. The reaction mixture was stirred for 10 h at 70 °C. After being cooled to room temperature, the reaction mixture was filtered on pad of celite and eluted with chloroform. The solvent was removed under reduced pressure, and the remaining brown oil was dissolved in THF (357 mL). At 0 °C, 1.09 mL acetic acid and 4.35 mL of 1 m TBAF solution in THF were added. After 2 h, the solvent was removed under reduced pressure. The residue was purified by column chromatography on silica gel, elution with *n*‐hexane/ethyl acetate 1:1 afforded **6** as a yellow oil. TLC (*n*‐hexane/ethyl acetate 1:1): *R*_f_=0.44. Yield: 5.5 g (37 %) (Figure S14–S16). ^1^H‐NMR (500 MHz, CDCl_3_): *δ*=8.97 (d, *J=*0.6 Hz, 1 H, H‐6), 5.46 (ddd, *J=*10.4, 6.3, 0.5 Hz, 1 H, H‐1′), 4.12 (t, *J=*3.1 Hz, 1 H, H‐4′), 4.04 (dd, *J=*12.1, 3.1 Hz, 1 H, H‐5′), 4.00 (dd, *J=*12.1, 3.3 Hz, 1 H, H‐5′), 3.17 (dd, *J=*18.2, 6.4 Hz, 1 H, H‐2′), 2.37 ppm (dd, *J=*18.2, 10.6 Hz, 1 H, H‐2′). ^13^C‐NMR (125 MHz, CDCl_3_): *δ*=211.5 (C‐3′), 160.1 (C‐2/C‐4), 159.9 (C‐2/C‐4), 158.5 (C‐4), 158.4, 132.0 (C‐5′), 82.2 (C‐4′), 72.3 (C‐1′), 61.6 (C‐5′), 43.5 ppm (C‐2′). MALDI‐MS: *m*/*z* calcd for C_9_H_8_Cl_2_N_2_O_3_ [*M*+2 H^+^]^+^: 263.07; found: 264.92.

**Synthesis of 1β‐(2,4‐dichloropyrimidin‐5‐yl)‐1,2‐dideoxy‐d‐ribofuranose (7)**: Compound **6** (1 equiv, 5.46 g, 0.013 mol) was dissolved in acetonitrile (438 mL) and acetic acid (72 mL). At 0 °C, sodium triacetoxyborohydride (2.52 equiv, 11.1 g, 0.052 mol) was slowly added. The reaction mixture was stirred at 0 °C for 15 min. 72 mL of 50 % aqueous ethanol was added. All solvents were removed under reduced pressure, and the residue was purified by column chromatography on silica gel, elution with dichloromethane/methanol 95:5 afforded **7** as a white foam. TLC (dichloromethane/methanol: 95/5): *R*_f_=0.21. Yield: 3.9 g (70 %) (Figure S17–S19). ^1^H‐NMR (500 MHz, MeOD‐d_4_): *δ*=8.85 (d, *J=*0.6 Hz, 1 H, H‐6), 5.30 (dd, *J=*10.0, 5.9 Hz, 1 H, H‐1′), 4.32 (ddd, *J=*5.9, 3.0, 2.2 Hz, 1 H, H‐3′), 3.95 (td, *J=*4.5, 2.7 Hz, 1 H, H‐4′), 3.67 (dd, *J=*11.9, 4.3 Hz, 2 H, 2×H‐5′),2.44 (ddd, *J=*13.0, 11.0, 5.8, 1 H, H‐2′), 1.85 ppm (ddd, *J=*13.0, 10.0, 5.8 Hz, 1 H, H‐2′). ^13^C‐NMR (125 MHz, MeOD‐d_4_): *δ*=160.8 (C‐2/C‐4), 159.96 (C‐6), 159.94 (C‐2/C‐4), 135.1 (C‐5), 89.4 (C‐4′), 76.0 (C‐1′), 74.0 (C‐3′), 63.5 (C‐5′), 42.7 ppm (C‐2′). MALDI‐MS: *m*/*z* calcd for C_9_H_10_Cl_2_N_2_O_3_ [*M*+H^+^]^+^: 265.09; found: 266.74.

**Synthesis of 1β‐(2,4‐dichloropyrimidin‐5‐yl)‐1,2‐dideoxy‐5‐*O*‐(tert‐butyl‐dimethylsilyl)‐d‐ribofuranose (8)**: In a dried Schlenk flask compound **7** (1 equiv, 3.67 g, 0.014 mol) and imidazole (2.5 equiv, 2.36 g, 0.035 mol) were dissolved in dry DMF. *Tert*‐butyl‐dimethylsilyl chloride (1.2 equiv, 2.5 g, 0.017 mol) was slowly added to the reaction mixture. The mixture was stirred for 16 h at room temperature, and 150 mL of water were added. The aqueous phase was washed three times with 100 mL of ethyl acetate and dried over magnesium sulfate. The solvent was removed under reduced pressure. The remaining yellowish oil was purified by column chromatography on silica gel. Elution with *n*‐hexane/ethyl acetate 3:1 afforded **8** as a colorless oil. TLC (*n*‐hexane/ethyl acetate 3:1): *R*_f_=0.30. Yield: 3.99 g (76 %) (Figure S20–S22). ^1^H‐NMR (500 MHz, CDCl_3_): *δ*=8.79 (d, 1 H, H‐6), 5.35 (dd, *J=*5.9 Hz, 1 H, H‐1′), 4.46 (ddt, *J=*5.6, 2.2, 1.8 Hz, 1 H, H‐3′), 4.09 (ddd, *J=*4.2, 3.1, 2.3 Hz, 1 H, H‐4′), 3.79 (dd, *J=*10.8, 3.4 Hz, 1 H, H‐5′), 3.75 (dd, *J=*10.7, 4.3 Hz, 1 H, H‐5′), 2.50 (ddd, *J=*12.9, 5.9, 2.0 Hz, 1 H, H‐2′), 1.90 (ddd, *J=*13.0, 9.9, 5.7 Hz, 1 H, H‐2′), 0.82 (s, 9 H, C(CH_3_)_3_), 0.04 ppm (s, 6 H, Si(CH_3_)_2_). ^13^C‐NMR (125 MHz, CDCl_3_): *δ*=159.6 (C‐2/C‐4), 159.3 (C‐2/C‐4), 158.7 (C‐6), 133.6 (C‐5), 87.7 (C‐4′), 75.0 (C‐1′), 74.4 (C‐3′), 63.8 (C‐5′), 42.2 (C‐2′), 26.0 (*C*CH_3_), 18.4 (C*C*H_3_), −5.28 (SiCH_3_), −5.41 ppm (SiCH_3_). MALDI‐MS: *m*/*z* calcd for C_15_H_24_Cl_2_N_2_O_3_Si [*M*+H^+^]^+^: 379.35; found: 380.58.

**Synthesis of 1β‐[2,4‐bis(benzoylamino)pyrimidin‐5‐yl]‐1,2‐dideoxy‐5‐*O*‐(tert‐butyl‐dimethylsilyl)‐d‐ribofuranose (9)**: In an argon purged dry Schlenk flask were dissolved in dry 1,4‐dioxane Pd_2_(dba)_3_ (0.04 equiv, 0.39 g, 0.42 mmol), xantphos (0.12 equiv, 0.73 g, 1.26 mmol), benzamide (3 equiv, 3.83 g, 0.032 mol), Cs_2_CO_3_ (3 equiv, 10.3 g, 0.032 mol) and compound **8** (1 equiv, 3.99 g, 0.011 mol). The reaction mixture was stirred for 24 hours at 100 °C. After being cooled to room temperature, the mixture was filtered over pad of celite and eluted with MeOH. The solvent was removed under reduced pressure and the remaining crude product was purified by column chromatography on silica gel. Elution with *n*‐hexane/ethyl acetate 1:3 afforded **9** as a colorless solid. TLC (ethyl acetate/*n*‐hexane 3:1): *R*_f_=0.08. Yield: 2.31 g (37 %) (Figure S23–S25). ^1^H‐NMR (500 MHz, MeOD‐d_4_): *δ*=8.56 (s, 1 H, H‐6), 8.07–8.05 (m, 2 H), 8.02–8.00 (m, 2 H), 7.69–7.62 (m, 2 H), 7.60–7.53 (m, 4 H), 5.40 (dd, *J=*10.6, 5.4 Hz, 1 H, H‐1′), 4.39 (dt, *J=*5.4, 1.4 Hz, 1 H, H‐3′), 4.11 (td, *J=*3.7, 2.1 Hz, 1 H, H‐4′), 3.75 (s, 1 H, H‐5′), 3.74 (s, 1 H, H‐5′), 2.43 (ddd, *J=*12.8, 5.4, 1.2 Hz, 1 H, H‐2′), 2.24–2.19 (m, 1 H, H‐2′), 0.80 (s, 9 H, C(CH_3_)_3_), −0.02 (s, 3 H, Si(CH_3_)_2_), −0.01 ppm (s, 3 H, Si(CH_3_)_2_). ^13^C‐NMR (125 MHz, MeOD‐d_4_): *δ*=167.5 (C_amide_), 167.2 (C_amide_), 158.2 (C‐2/C‐4), 158.1 (C‐2/C‐4), 157.9 (C‐6), 135.4 (C_quart._), 135.1 (C_quart._), 134.2, 133.7, 130.1, 129.8, 128.9, 128.9 (CH_ar._), 120.3 (C‐5), 89.8 (C‐4′), 77.4 (C‐1′), 73.8 (C‐3′), 64.8 (C‐5′), 42.3 (C‐2′), 26.3 (*C*CH_3_), 19.1 (C*C*H_3_), −5.4 (SiCH_3_), −5.5 ppm (SiCH_3_). MALDI‐MS: *m*/*z* calcd for C_29_H_36_N_4_O_5_Si [*M*+H^+^]^+^: 549.61; found: 548.61.

**Synthesis of 1β‐[2,4‐bis(benzoylamino)pyrimidin‐5‐yl]‐1,2‐dideoxy‐d‐ribofuranose (10)**: Compound **9** (1 equiv, 2.31 g, 4.21 mmol) was dissolved in 59 mL THF. NEt_3_⋅3 HF (2.6 equiv, 1.78 mL, 10.93 mmol) was added dropwise. After two hours, volatiles were removed under reduced pressure, and the remaining solid was purified by column chromatography on silica gel. Elution with dichloromethane/methanol 9:1 afforded **10** as a white solid. TLC (dichloromethane/methanol 9:1): *R*_f_=0.25. Yield: 1.2 g (62 %) (Figure S26–S28). ^1^H‐NMR (500 MHz, [D_6_]DMSO): *δ*=11.05 (s, 1 H, NH), 10.86 (s, 1 H, NH), 8.91 (s, 1 H, H‐6), 8.01–7.96 (m, 4 H), 7.66–7.49 (m, 6 H), 5.13 (dd, *J=*10.1, 5.6 Hz, 1 H, H‐1′), 5.03 (d, *J=*4.1 Hz, 1 H), 4.83 (t, *J=*5.9 Hz, 1 H), 4.18–4.17 (m, 1 H), 3.75 (td, *J=*4.8, 2.5 Hz, 1 H), 3.49 (t, *J=*5.2 Hz, 2 H, H‐5′), 2.21 (ddd, *J=*12.9, 5.6, 1.7 Hz, 1 H, H‐2′), 1.89 ppm (ddd, *J=*12.9, 10.3, 5.9 Hz, 1 H, H‐2′). ^13^C‐NMR (125 MHz, [D_6_]DMSO): *δ*=166.1 (C_amide_), 165.6 (C_amide_), 158.3 (C‐6), 157.1 (C‐2/C‐4), 156.4 (C‐2/C‐4), 134.1 (C_quart._), 133.1 (C_quart._), 132.5, 132.1 (CH_ar._), 128.6, 128.4, 128.2, 128.1 (CH_ar._), 126.2 (CH_quart._), 115.2 (C‐5), 87.6 (C‐4′), 74.0 (C‐1′), 72.1 (C‐3′), 62.1 (C‐5′), 41.6 ppm (C‐2′). MALDI‐MS: *m*/*z* calcd for C_23_H_22_N_4_O_5_ [*M*+H^+^]^+^: 434.44; found: 434.72.

**Synthesis of 1β‐[2,4‐bis(benzoylamino)pyrimidin‐5‐yl]‐1,2‐dideoxy‐5‐*O*‐(4,4′‐dimethoxytriphenylmethyl)‐d‐ribofuranose (11)**: In an argon purged dried Schlenk flask compound **10** (1 equiv, 1.15 g, 2.65 mmol) and DMAP (0.05 equiv, 16 mg, 0.133 mmol) were dissolved in 32 dry pyridine. 4,4′‐dimethoxytrityl chloride (1.5 equiv, 1.35 g, 4.0 mmol) was added slowly and the mixture was stirred at room temperature for 12 h. The reaction was stopped with 13 mL of MeOH and all solvents were removed under reduced pressure. The remaining crude product was purified by column chromatography on silica gel. Elution with *n*‐hexane/acetone 2:3 +0.1 % NEt_3_ afforded **11** as a white foam. TLC (*n*‐hexane/acetone 2:3): *R*_f_=0.23. Yield: 1.49 g (77 %) (Figure S29–S31). ^1^H‐NMR (500 MHz, CDCl_3_): *δ*=10.49 (s, 1 H, NH), 9.74 (s, 1 H, NH), 8.43 (s, 1 H, H‐6), 7.99 (d, *J=*7.4 Hz, 2 H), 7.82 (d, *J=*7.8 Hz, 2 H), 7.52–7.26 (m, 8 H), 7.18–7.14 (m, 4 H), 6.70–6.68 (m, 4 H), 5.28 (dd, *J=*10.3, 4.9 Hz, 1 H, H‐1′), 4.55 (br s, 1 H, OH), 4.30 (br s, 1 H, H‐3′), 3.79 (t, 1 H, H‐4′), 3.70 (s, 6 H, 2 *x* OCH_3_), 3.27 (dd, *J=*10.3, 5.1 Hz, 1 H, H‐5′), 3.19 (dd, *J=*10.1, 4.5 Hz, 1 H, H‐5′), 2.51–2.49 (m, 1 H, H‐2′), 2.30 ppm (dd, *J=*12.4, 5.6 Hz, 1 H, H‐2′). ^13^C‐NMR (125 MHz, CDCl_3_): *δ*=165.3 (C_amide_), 164.9 (C_amide_), 158.6 (C_ar._‐O), 157.5 (C‐2/C‐4), 157.2 (C‐2/C‐4), 156.4 (C‐6), 144.5, 135.7, 135.7, 133.8 (C_quart._), 132.9, 132.4, 130.0, 129.1, 128.7, 128.1, 127.9, 127.8, 127.6, 113.2 (CH_ar._), 87.4 (C‐4′), 86.5 (C_quart._), 76.1 (C‐1′), 73.6 (C‐3′), 64.3 (C‐5′), 55.3 (O*C*H_3_), 40.0 ppm (C‐2′). MALDI‐MS: *m*/*z* calcd for C_44_H_40_N_4_O_7_ [*M*‐H^+^]^+^: 736.81; found: 736.45.

**Synthesis of 1β‐[2,4‐Bis(benzoylamino)pyrimidin‐5‐yl]‐1,2‐dideoxy‐5‐*O*‐(4,4′‐dimethoxytriphenylmethyl)‐d‐ribofuranose‐3‐[(2‐cyanoethyl)(*N***,***N*** **‐diisopropyl)]phosphoramidite (12)**: In an argon purged dried Schlenk flask compound **11** (1 equiv, 253 mg, 0.343 mmol) and *N*,*N*‐diisopropylethylamine (3 equiv, 0.18 mL, 1.03 mmol) were dissolved in 8 mL of dry dichloromethane. 2‐cyanoethyl *N*,*N*‐diisopropylethylamine (1.5 equiv, 0.115 mL, 0.515 mmol) was added and the reaction mixture was stirred for two hours at room temperature. All volatiles were removed under reduced pressure and crude product was purified by column chromatography on silica gel. Elution with cyclohexane/acetone 2:1 +0.1 % triethylamine afforded **12** as a colorless solid. TLC (cyclohexane/acetone 2:1): *R*_f_=0.31. Yield: 238 mg (74 %) (Figure S32–S34). ^31^P‐NMR (202 MHz, [D_6_]DMSO): *δ*=147.1, 146.5 ppm. ^1^H‐NMR (400 MHz, [D_6_]DMSO): *δ*=11.10 (s, 1 H, NH), 11,01 (s, 1 H, NH), 8.88 (d, *J=*2.4 Hz, 1 H, H‐6), 8.04–7.97 (m, 4 H, Phenyl), 7.67–7.50 (m, 7 H, Phenyl), 7.34–7.21 (m, 8 H, Phenyl), 6.89 (dd, *J=*3.8, 8.8 Hz, 4 H, Phenyl), 5.14 (m, 1 H, H‐1′), 4.40 (m, 1 H, H‐4′), 4.02 (m, 1 H, H‐3′), 3.73 (s, 6 H, 2×OCH_3_), 3.57 (m, 1 H, H‐5′), 3.46 (m, 1 H, H‐5′), 3.42 (m, 2 H, Alkan), 3.19 (m, 2 H, H‐2′), 2.56 (m, 2 H, Isopropyl), 2.48 (m, 2 H, Alkan), 1.04 (m, 6 H, 2xCH_3_), 0.90 ppm (m, 6 H, 2×CH_3_). MALDI‐MS: *m*/*z* calcd for C_53_H_57_N_6_O_8_P [*M*‐H^+^]: 937.03; found: 936.36.

**Synthesis of** ***N*** **‐(4‐chloro‐7*H*‐pyrrolo[2,3‐d]pyrimidin‐2‐yl)‐2,2‐dimethyl‐propionamide (14)**: Compound **13** (1 equiv., 14 g, 83 mmol) was dissolved in 196 mL of dry pyridine under an argon atmosphere. Pivaloyl chloride (1.8 equiv., 18.5 mL, 151 mmol) was then slowly added dropwise to this solution at room temperature. The reaction mixture was stirred for one hour at room temperature. During the reaction, a colorless solid precipitated (pyridine hydrochloride). By adding 100 mL of methanol, the reaction was stopped. The solvent was removed in an oil pump vacuum leaving a yellow solid. The yellow solid was taken up in 200 mL of ethyl acetate and washed three times with 100 mL of a 0.1 m hydrochloric acid solution. The organic phase was dried over magnesium sulfate (MgSO_4_) and the solvent removed in vacuo. The crude product was purified by column chromatography. The eluent used was dichloromethane/methanol in a ratio of 10:1. Compound **14** was obtained as a yellowish solid. TLC (dichloromethane/methanol 98:2): *R*_f_=0.37. Yield: 19 g (90 %) (Figure S35–S37). ^1^H‐NMR (500 MHz, [D_6_]DMSO): *δ*=12.33 (s, 1 H, NH), 10.03 (s, 1 H, NH), 7.54 (d, *J=*3.6 Hz, 1 H, H‐7), 6.52 (d, *J=*3.5 Hz, 1 H, H‐8), 1.23 ppm (s, 9 H, *tert*Butyl). ^13^C‐NMR (126 MHz, [D_6_]DMSO): *δ*=175.9, 152.8, 151.4, 150.2, 127.4, 113.2, 98.9, 39.4, 26.9 ppm. ESI‐MS: *m*/*z* calcd for C_11_H_13_ClN_4_O [*M*+H^+^]^+^ 252.70; found: 253.11.

**Synthesis of** ***N*** **‐(4‐chloro‐5‐iodo‐7*H*‐pyrrolo[2,3‐d]pyrimidin‐2‐yl)‐2,2‐dimethyl‐propionamide (15)**: Compound **14** (1 equiv., 18 g, 71.2 mmol) was dissolved under argon atmosphere in 360 mL dry tetrahydrofuran. To this solution was then added *N*‐iodosuccinimide (1.1 equiv., 17.6 g, 78.4 mmol). The reaction mixture was stirred for one hour at room temperature. Then the solvent was removed in vacuo and the residue was taken up in 1.5 L dichloromethane. It was then washed first with a saturated sodium sulfate solution (Na_2_S_4_), then with a saturated sodium chloride solution and then dried over magnesium sulfate. The solvent was removed in vacuo and the residue was purified by column chromatography. The eluent used was dichloromethane/methanol in the ratio 20:1. Compound **15** was isolated as a colorless solid. TLC (dichloromethane/methanol 20:1): *R*_f_=0.77. Yield: 25,1 g (93 %) (Figure S38–S40). ^1^H‐NMR (600 MHz, [D_6_]DMSO): *δ*=12.67 (s, 1 H, NH), 10.09 (s, 1 H, NH), 7.76 (d, *J=*2.2 Hz, 1 H, H‐8), 1.22 ppm (s, 9 H, *tertButyl*). ^13^C‐NMR (151 MHz, [D_6_]DMSO): *δ*=175.8, 152.5, 151.4, 150.7, 132.7, 112.3, 51.5, 26.8 ppm. ESI‐MS: *m*/*z* calcd for C_11_H_12_ClIN_4_O [*M*+Na^+^]^+^ 378.60; found: 400.90.

**Synthesis of 4‐chloro‐5‐iodo‐2‐pivaloylamino‐7‐[(2,3,5‐tri‐*O*‐benzoyl)‐β‐d‐ribofuranosyl]‐7*H*‐pyrrolo[2,3‐*d*]pyrimidine (16)**: Compound **15** (1 equiv., 15 g, 39.6 mmol) was suspended under an argon atmosphere in 350 mL of dry acetonitrile. To this was added dropwise *N*,*O*‐bis(trimethylsilyl) acetamide (1.24 equiv., 12 mL, 49.1 mmol) and the solid went completely into solution. The reaction mixture was stirred for ten minutes at room temperature. Then, trimethylsilyl trifluoro‐methanesulfonate (1.4 equiv., 10 mL, 55.5 mmol) and 1′‐acetate‐2′,3′,5′‐tribenzoate‐β‐d‐ribofuranose (0.67 equiv., 13, 4 g, 26.6 mmol) were added and heated to 50 °C. 1′‐acetate‐2′,3′,5′‐tribenzoate‐β‐d‐ribofuranose (0.67 equiv., 13.4 g, 26.6 mmol) was then added again after six and twelve hours. After stirring for 24 hours, 800 mL of dichloromethane were added. The organic phase was washed with 150 mL of a saturated aqueous sodium bicarbonate solution, with 150 mL of a saturated sodium chloride solution (evolution of gas) and dried over magnesium sulfate. The solvent was removed in vacuo and a column chromatographic purification was performed. The eluent used was cyclohexane/ ethyl acetate in the ratio 3:1. The product **16** was isolated as a yellow/colorless foam. TLC (cyclohexane/ethyl acetate 3:1): *R*_f_=0.33. Yield: 19 g (58 %) (Figure S41–S43). ^1^H‐NMR (600 MHz, [D_6_]DMSO): *δ*=10.32 (s, 1 H, NH), 8.06 (s, 1 H, H‐8), 7.93–7.89 (m, 6 H, Aromat), 7.66–7.62 (m, 4 H, Aromat), 7.48–7.43 (m, 6 H, Aromat), 6.52 (d, *J=*3.8 Hz, 1 H, H‐1′), 6.44 (t, *J=*5.9, 6.2 Hz, 1 H, H‐3′), 6.36 (dd, *J=*3.8, 6.0 Hz, 1 H, H‐2′), 4.84 (dd, *J=*5.3, 10.5 Hz, 1 H, H‐4′), 4.79 (dd, *J=*4.3, 11.7 Hz, 1 H, H‐5′), 4.66 ppm (dd, *J=*5.6, 11.5 Hz, H‐5′). ^13^C‐NMR (151 MHz, [D_6_]DMSO): *δ*=175.8, 165.3, 164.5, 164.4, 151.8, 151.5, 151.3, 133.9, 133.7, 133.67, 133.4, 129.4, 129.24, 129.22, 129.1, 128.8, 128.72, 128.70, 128.6, 128.4, 113.2, 87.6, 79.1, 73.9, 71.2, 63.7, 53.97, 26.6 ppm. ESI‐MS: *m*/*z* calcd for C_37_H3_2_ClIN_4_O_8_ [*M*+H^+^]^+^ 823.03; found: 823.19.

**Synthesis of 2‐amino‐5‐iodo‐3,7‐dihydro‐7‐(β‐d‐ribofuranosyl)‐4*H*‐pyrrolo‐[2,3‐d]pyrimidin‐4‐one (17)**: Compound **16** (1 equiv., 18.95 g, 23.02 mmol) was dissolved in 345 mL of a 0.5 m sodium methoxide solution and heated at reflux for 12 hours. It was then neutralized with glacial acetic acid and the solvent was removed in vacuo. The remaining residue was purified by column chromatography. The eluent used was dichloromethane/methanol in the ratio 9:1. The product **17** was isolated as a colorless solid. TLC (dichloromethane/methanol 9:1): *R*_f_=0.29. Yield: 9,7 g (61 %) (Figure S44–S46). ^1^H‐NMR (600 MHz, [D_6_]DMSO): *δ*=7.31 (s, 1 H, H‐8), 6.36 (s, 2 H, NH_2_), 5.94 (d, *J=*6.6 Hz, 1 H, H‐1′), 5.25 (d, *J=*4.7 Hz, 1 H, OH‐2′), 5.05 (s, 1 H, OH‐3′), 4.99 (t, *J=*4.9, 5.2 Hz, OH‐5′), 4.27 (dd, *J=*4.6, 6.2 Hz, 1 H, H‐2′), 4.02 (s, 1 H, H‐3′), 3.92 (s, 3 H, CH_3_), 3.81 (dd, *J=*3.9, 6.8 Hz, 1 H, H‐4′), 3.58–3.56 (m, 1 H, H‐5′), 3.50–3.49 ppm (m, 1 H, H‐5′). ^13^C‐NMR (151 MHz, [D_6_]DMSO): *δ*=162.8, 159.4, 154.7, 124.4, 98.7, 85.7, 84.7, 73.5, 70.5, 61.5, 52.96, 51.59 ppm. ESI‐MS: *m*/*z* calcd for C_12_H_15_IN_4_O_5_ [*M*+H^+^]^+^ 422.18; found: 423.00.

**Synthesis of 1,7‐dihydro‐5‐iodo‐4‐methoxy‐7‐(β‐d‐ribofuranosyl)‐2*H*‐pyrrolo[2,3‐d]pyrimidin‐2‐amine (18)**: Compound **17** (1 equiv., 2.5 g, 5.92 mmol) was suspended in 505 mL of a 7:1 mixture of water (433 mL) and glacial acetic acid (72 mL). To this was then slowly added dropwise sodium nitrite (2.46 equiv., 1.01 g, 14.57 mmol) dissolved in 11.2 mL of water. After about ten minutes, a yellow‐orange solution formed. The reaction mixture was stirred for one hour at room temperature. It was then neutralized with a 25 % aqueous ammonia solution. The solvent was removed in vacuo and the residue was purified by column chromatography. The eluent used was dichloromethane/ methanol in the ratio 5:1. Compound **18** was isolated as a yellowish solid. TLC (dichloromethane/methanol 5:1): *R*_f_=0.52. Yield: 1,5 g (61 %) (Figure S47–S49). ^1^H‐NMR (600 MHz, [D_6_]DMSO): *δ*=11.60 (s, 1 H, NH), 7.48 (s, 1 H, H‐8), 5.94 (d, *J=*6.1 Hz, 1 H, H‐1′), 5.31 (s, 1 H, OH‐2′), 5.10 (s, 2 H, OH‐3′+ OH‐5′), 4.28 (dd, *J=*5.6, 6.0 Hz, 1 H, H‐2′), 4.04 (s, 1 H, H‐3′), 3.97 (s, 3 H, CH_3_), 3.87 (m, 1 H, H‐4′), 3.61–3.58 (ddd, *J=*3.5, 7.8, 11.2 Hz, 1 H, H‐5′), 3.54–3.52 ppm (ddd, *J=*3.5, 8.2, 11.7 Hz, 1 H, H‐5′): ^13^C‐NMR (151 MHz, [D_6_]DMSO): *δ*=172.2, 163.9, 160.1, 126.0, 100.5, 86.6, 85.2, 73.8, 70.5, 61.5, 53.6, 52.1 ppm. ESI‐MS: *m*/*z* calcd for C_12_H_14_IN_3_O_6_ [*M*+H^+^]^+^ 423.16; found: 423.98

**Synthesis of 1,7‐dihydro‐5‐(octa‐1,7‐diynyl)‐4‐methoxy‐7‐(β‐d‐ribofuranosyl)‐2*H*‐pyrrolo[2,3‐d]pyrimidin‐2‐amine (19)**: Compound **18** (1 equiv., 1.2 g, 2.84 mmol) was first dried in vacuo at 65 °C for one hour. Then, tetrakis(triphenylphosphine)palladium(0) (Pd (PPh3) 4, 0.1 Eq., 328 mg, 284 μmol) and copper iodide (CuI, 0.2 Eq., 108 mg, 567 μmol) were added under argon atmosphere. The solids were again dried in vacuo for 20 minutes. To this was then added 10 mL of *N*,*N*‐dimethylformamide and triethylamine (NEt3, 2.84 equiv., 1.12 mL, 8.05 mmol). The reaction solution was then placed under argon and vacuum 20 times to remove any residual oxygen. Finally, 1,7‐octadiyne (10 equiv., 3.76 mL, 28.4 mmol) was added. The reaction was stirred at room temperature overnight. Thereafter, the solvent was removed in an oil pump vacuum at 65 °C to 90 %. Subsequently, a column chromatographic purification was carried out. The eluent used was dichloromethane/ methanol in the ratio 9:1. The product **19** was isolated as an orange solid. Yield: 1.09 g (96 %) (Figure S50–S52). The product still contains residues of triethylamine, which could not be removed by the column‐chromatographic purification. TLC (dichloromethane/methanol 9:1): *R*_f_=0.39. ^1^H‐NMR (600 MHz, [D_6_]DMSO): *δ*=11.45 (s, 1 H, NH), 7.53 (s, 1 H, H‐8), 5.97 (s, 1 H, OH‐2′), 5.32 (d, *J=*6.2 Hz, 1 H, H‐1′), 5.10 (s, 1 H, OH‐3′), 5.03 (s, 1 H, OH‐5′), 4.29 (s, 1 H, H‐2′), 4.05 (dd, *J=*4.5, 7.6, 12.1 Hz, 1 H, H‐3′), 3.97 (s, 3 H, CH_3_), 3.86 (s, 1 H, H‐4′), 3.61–3.59 (m, 1 H, H‐5′), 3.54–3.51 (m, 1 H, H‐5′), 2.79 (t, *J=*2.6 Hz, 1 H, H‐a), 2.43 (t, *J=*6.5 Hz, 2 H, H‐b), 2.25–2.22 (m, 2 H, H‐e), 1.68–1.60 ppm (m, 4 H, H‐c,d). ^13^C‐NMR (126 MHz, [D_6_]DMSO): *δ*=164.2, 134.9, 133.8, 133.7, 130.1, 130.0, 125.2, 90.3, 86.3, 85.1, 84.4, 73.8, 71.3, 70.5, 61.4, 53.6, 27.3, 27.0, 18.4, 17.3 ppm. ESI‐MS: *m*/*z* calcd for C_20_H_23_N_3_O_6_ [*M*+Na^+^]^+^ 401.41; found: 424.13.

**Synthesis of 5‐(octa‐1,7‐diynyl)‐7‐(β‐d‐ribofuranosyl)‐1,3,7‐trihydro‐2*H*,4*H*‐pyrrolo‐[2,3‐d]pyrimidin‐2,4‐dione (20)**: Compound **19** (1 equiv., 750 mg, 1.87 mmol) and sodium iodide (1.97 equiv., 197 mg, 1.32 mmol) were suspended under an argon atmosphere in 28 mL of dry acetonitrile. To this suspension trimethylsilyl chloride (TMS‐Cl, 2.28 equiv., 193 μL, 1.52 mmol) was added. A greenish discoloration of the reaction solution was observed. The reaction was stirred for one hour at room temperature. The reaction solution was filtered and washed with acetonitrile. The solvent was removed in vacuo and a column chromatographic purification followed. The eluent used was dichloromethane/ methanol in the ratio 5:1. The product **20** was isolated as a colorless solid. TLC (dichloromethane/methanol 5:1): *R*_f_=0.56. Yield: 453 mg (63 %) (Figure S53–S55). ^1^H‐NMR (500 MHz, [D_6_]DMSO): *δ*=11.52 (s, 1 H, NH), 10.64 (s, 1 H, NH), 6.90 (s, 1 H, H‐8), 5.77 (m, 1 H, OH), 5.66 (d, *J=*6.9 Hz, 1 H, H‐1′), 5.37 (d, *J=*6.6 Hz, OH), 5.21 (d, *J=*3.9 Hz, 1 H, OH), 4.16–4.15 (m, 1 H, H‐2′), 4.01 (m, 1 H, H‐3′), 3.96–3.95 (m, 1 H, H‐4′), 3.62 (m, 2 H, H‐5′), 2.70 (t, *J=*2.6, 5.3 Hz, 1 H, H‐a), 2.08–2.06 (m, 2 H, H‐b), 1.95–1.93 (m, 2 H, H‐e), 1.53–137 ppm (m, 4 H, H‐c,d). ^13^C‐NMR (126 MHz, [D_6_]DMSO): *δ*=158.9, 151.1, 138.9, 120.7, 117.4, 97.8, 94.9, 89.7, 86.0, 84.9, 84.8, 74.5, 74.4, 71.8, 71.2, 61.8, 27.9, 27.7, 27.5, 18.1, 17.9 ppm. ESI‐MS: *m*/*z* calcd for C_19_H_21_N_3_O_6_ [*M*‐H^+^]^−^ 387.39; found: 386.23.

**Synthesis of 5‐(octa‐1,7‐diynyl)‐7‐(β‐d‐ribofuranosyl)‐1,3,7‐trihydro‐2*H*,4*H*‐pyrrolo‐[2,3‐d]pyrimidin‐2,4‐dione TP (21)**: Compound **20** (1 equiv., 50 mg, 129 μmol) and freshly triturated proton sponge (1.5 equiv., 41.5 mg, 194 μmol) were dried in a predried Schlenk flask overnight in an oil pump vacuum. It was the dissolved under an argon atmosphere in 1.6 mL trimethyl phosphate (dried over a 4 Å molecular sieve for 4 days). The reaction solution was cooled to −7.5 °C and freshly distilled phosphorus oxychloride (1.05 equiv., 12.7 μL, 136 μmol) was rapidly added. After five hours, phosphorus oxychloride (0.4 equiv., 4.8 μL, 51.6 μmol) was added again, and the reaction mixture was further stirred for one hour. Then tributylamine (4.4 equiv., 135 μL, 568 μmol) and 2.5 mL of 0.4 m tributylamine pyrophosphate solution in dry DMF were added. The mixture was further stirred at room temperature for 30 minutes. Next, the reaction mixture was slowly added dropwise to a rapidly stirring solution of 0.1 m TEAB buffer (pH 8, 9.2 mL). The solution was hydrolyzed for eight hours and the product **21** was isolated by RP‐HPLC. The fractions containing the desired product were lyophilized. **21** was isolated as a colorless solid. Yield: 13 mg (16 %) (Figure S56–S59). ^1^H‐NMR (600 MHz, D_2_O): *δ*=7.89 (s, 1 H, H‐8), 6.08 (d, *J=*6.5 Hz, 1 H, H‐1′), 4.59 (t, *J=*5.9 Hz, 1 H, H‐2′), 4.48 (dd, *J=*3.3, 5.5 Hz, 1 H, H‐3′), 4.33–4.32 (m, 1 H, H‐4′), 4.28–4.24 (m, 2 H, H‐5′), 2.45 (t, *J=*6.6 Hz, 1 H, H‐a), 2.27–2.26 (m, 2 H, H‐b), 2.19–2.16 (m, 2 H, H‐e), 1.69–1.65 ppm (m, 4 H, H‐c,d). ^31^P‐NMR (162 MHz, D_2_O): *δ*=−5.48 (d, 1P, γ‐PO_3_^2−^), −10.62 (d, 1P, α‐PO_3_^2−^), −18.88 ppm (t, 1P, β‐PO_3_^2−^). MALDI‐MS: *m*/*z* calcd for C_19_H_24_N_3_O_15_P_3_ [*M*+Na^+^]^+^ 627.33; found: 650.0450.

## Conflict of interest

The authors declare no conflict of interest.

## Supporting information

As a service to our authors and readers, this journal provides supporting information supplied by the authors. Such materials are peer reviewed and may be re‐organized for online delivery, but are not copy‐edited or typeset. Technical support issues arising from supporting information (other than missing files) should be addressed to the authors.

Supplementary

## References

[1] Y. Zhang , J. L. Ptacin , E. C. Fischer , H. R. Aerni , C. E. Caffaro , K. S. Jose , A. W. Feldman , C. R. Turner , F. E. Romesberg , Nature 2017, 551, 644–647. 29189780 10.1038/nature24659 PMC5796663

[2] A. W. Feldman , V. T. Dien , R. J. Karadeema , E. C. Fischer , Y. You , B. A. Anderson , R. Krishnamurthy , J. S. Chen , L. Li , F. E. Romesberg , J. Am. Chem. Soc. 2019, 141, 10644–10653. 31241334 10.1021/jacs.9b02075 PMC6693872

[3] V. T. Dien , M. Holcomb , F. E. Romesberg , Biochemistry 2019, 58, 2581–2583. 31117391 10.1021/acs.biochem.9b00274 PMC6676896

[4] J. Piccirilli , T. Krauch , S. E. Moroney , S. Benner , Nature 1990, 343, 33–37. 1688644 10.1038/343033a0

[5] A. Rich in On the Problems of Evolution and Biochemical Information Transfer (Eds.: M. Kasha, B. Pullman), Academic Press, New York 1962, pp. 103–26.

[6] Z. Yang , D. Hutter , P. Sheng , A. M. Sismour , S. A. Benner , Nucleic Acids Res. 2006, 34, 6095–6101. 17074747 10.1093/nar/gkl633 PMC1635279

[7] H. J. Kim , N. A. Leal , S. A. Benner , Bioorg. Med. Chem. 2009, 17, 3728–3732. 19394831 10.1016/j.bmc.2009.03.047 PMC5972679

[8] F. Eggert , S. Kath-Schorr , Chem. Commun. 2016, 52, 7284–7287. 10.1039/c6cc02321e 27181840

[9] C. Domnick , F. Eggert , S. Kath-Schorr , Chem. Commun. 2015, 51, 8253–8256. 10.1039/c5cc01765c 25874847

[10] F. Eggert , K. Kulikov , C. Domnick , P. Leifels , S. Kath-Schorr , Methods 2017, 120, 17–27. 28454775 10.1016/j.ymeth.2017.04.021

[11] C. Domnick , G. Hagelueken , F. Eggert , O. Schiemann , S. Kath-Schorr , Org. Biomol. Chem. 2019, 17, 1805–1808. 30520916 10.1039/c8ob02597e

[12] E. Larsen , P. T. Jorgensen , M. A. Sofan , E. B. Pedersen , Synthesis 1994, 1037–1038.

[13] M. A. Cameron , S. B. Cush , R. P. Hammer , J. Org. Chem. 1997, 62, 9065–9069.

[14] M. Weinberger , F. Berndt , R. Mahrwald , N. P. Ernsting , H. A. Wagenknecht , J. Org. Chem. 2013, 78, 2589–2599. 23383743 10.1021/jo302768f

[15] T. Kubelka , L. Slavětínská , B. Klepetářová , M. Hocek , Eur. J. Org. Chem. 2010, 2666–2669.

[16] N. Joubert , R. Pohl , B. Klepetářová , M. Hocek , J. Org. Chem. 2007, 72, 6797–6805. 17665955 10.1021/jo0709504

[17] A. Häberli , C. J. Leumann , Org. Lett. 2001, 3, 489–492. 11428045 10.1021/ol007029s

[18] W. Saenger , Principles of Nucleic Acid Structure, Springer, New York, 1984, p. 21.

[19] M. Singer , A. Nierth , A. Jäschke , Eur. J. Org. Chem. 2013, 2766–2769.

[20] F. Seela , X. Peng , Collect. Czech. Chem. Commun. 2006, 71, 956–977.

[21] F. Seela , X. Peng , J. Org. Chem. 2005, 81–90. 10.1021/jo0516640 16388621

[22] X. Peng , F. Seela , Nucleosides Nucleotides Nucleic Acids 2007, 26, 603–606. 18066863 10.1080/15257770701490332

[23] C. Weldon , I. Behm-Ansmant , L. H. Hurley , G. A. Burley , C. Branlant , I. C. Eperon , C. Dominguez , Nat. Chem. Biol. 2017, 13, 18–20. 27820800 10.1038/nchembio.2228 PMC5164935

[24] F. Seela , N. Ramzaeva , Helv. Chim. Acta 1995, 78, 1083–1090.

[25] F. Klepper , E. M. Jahn , V. Hickmann , T. Carell , Angew. Chem. Int. Ed. 2007, 46, 2325–2327; 10.1002/anie.200604579 17310487

[26] F. Seela , M. Zulauf , Synthesis 1995, 726–730.

[27] J. Ludwig , F. Eckstein , J. Org. Chem. 1989, 54, 631–635.

[28] M. Yoshikawa , T. Kato , T. Takenishi , Tetrahedron Lett. 1967, 8, 5065–5068. 10.1016/s0040-4039(01)89915-9 6081184

[29] J. Ludwig , Acta Biochim. Biophys. Acad. Sci. Hung. 1981, 16, 131–133. 7347985

[30] M. Merkel , S. Arndt , D. Ploschik , G. B. Cserép , U. Wenge , P. Kele , H. A. Wagenknecht , J. Org. Chem. 2016, 81, 7527–7538. 27513089 10.1021/acs.joc.6b01205

[31] J. F. Milligan , D. R. Groebe , G. W. Whherell , O. C. Uhlenbeck , Nucleic Acids Res. 1987, 15, 8783–8798. 3684574 10.1093/nar/15.21.8783 PMC306405

[32] B. Fürtig , C. Richter , W. Bermel , H. Schwalbe , J. Biomol. NMR 2004, 28, 69–79. 14739640 10.1023/B:JNMR.0000012863.63522.1f

[33] S. Nozinovic , B. Fürtig , H. R. A. Jonker , C. Richter , H. Schwalbe , Nucleic Acids Res. 2010, 38, 683–694. 19906714 10.1093/nar/gkp956 PMC2811024

[34] H. Steinert , F. Sochor , A. Wacker , J. Buck , C. Helmling , F. Hiller , S. Keyhani , J. Noeske , S. Grimm , M. M. Rudolph , H. Keller , R. A. Mooney , R. Landick , B. Suess , B. Fürtig , J. Wöhnert , H. Schwalbe , eLife 2017, 6, 1–18. 10.7554/eLife.21297 PMC5459577 28541183

[35] J. Buck , J. Noeske , J. Wöhnert , H. Schwalbe , Nucleic Acids Res. 2010, 38, 4143–4153. 20200045 10.1093/nar/gkq138 PMC2896527

[36] J. Buck , B. Fürtig , J. Noeske , J. Wönert , H. Schwalbe , Proc. Natl. Acad. Sci. USA 2007, 104, 15699–15704. 17895388 10.1073/pnas.0703182104 PMC2000436

[37] J. Noeske , J. Buck , B. Fürtig , H. R. Nasiri , H. Schwalbe , J. Wöhnert , Nucleic Acids Res. 2007, 35, 572–583. 17175531 10.1093/nar/gkl1094 PMC1802621

[38] J. Singh , N. Steck , D. De , A. Hofer , A. Ripp , I. Captain , M. Keller , P. A. Wender , R. Bhandari , H. J. Jessen , Angew. Chem. Int. Ed. 2019, 58, 3928–3933; 10.1002/anie.201814366 30681761

[39] J. Schindelin , I. Arganda-Carreras , E. Frise , V. Kaynig , M. Longair , T. Pietzsch , S. Preibisch , C. Rueden , S. Saalfeld , B. Schmid , J.-Y. Tinevez , D. J. White , V. Hartenstein , K. Eliceiri , P. Tomancak , A. Cardona , Nat. Methods 2012, 9, 676–682. 22743772 10.1038/nmeth.2019 PMC3855844

[40] C. Helmling , S. Keyhani , F. Sochor , B. Fürtig , M. Hengesbach , H. Schwalbe , J. Biomol. NMR 2015, 63, 67–76. 26188386 10.1007/s10858-015-9967-y

[41] V. Sklenář , A. Bax , J. Magn. Reson. 1987, 74, 469–479.

[42] A. L. Breeze , Prog. Nucl. Magn. Reson. Spectrosc. 2000, 36, 323–372.

[43] J. Noeske , C. Richter , M. A. Grundl , H. R. Nasiri , H. Schwalbe , J. Wöhnert , Proc. Natl. Acad. Sci. USA 2005, 102, 1372–1377. 15665103 10.1073/pnas.0406347102 PMC547832

[44] K. Ogura , H. Terasawa , F. Inagaki , J. Biomol. NMR 1996, 8, 492–498. 20859780 10.1007/BF00228150

[45] C. Zwahlen , P. Legault , S. J. F. Vincent , J. Greenblatt , R. Konrat , L. E. Kay , J. Am. Chem. Soc. 1997, 119, 6711–6721.

[46] J. Iwahara , J. M. Wojciak , R. T. Clubb , J. Biomol. NMR 2001, 19, 231–241. 11330810 10.1023/a:1011296112710

[47] M. Liu , X. Mao , C. Ye , H. Huang , J. K. Nicholson , J. C. Lindon , J. Magn. Reson. 1998, 132, 125–129. 10.1006/jmre.1997.1246 9405217

