# Supplementary material for: Genetic Code Expansion Facilitates Position‐Selective Labeling of RNA for Biophysical Studies
Source: Chemistry. 2020 Jan 21;26(8):1800–10. doi: 10.1002/chem.201904623 (PMC7027469; doi:10.1002/chem.201904623)

CHEMISTRY
A European Journal

Supporting Information

Genetic Code Expansion Facilitates Position-Selective Labeling of RNA for Biophysical Studies

Andreas Hegelein+,[a] Diana Müller+,[a] Sylvester Größl,[b] Michael Göbel,[b]
Martin Hengesbach,[a] and Harald Schwalbe*[a]

chem_201904623_sm_miscellaneous_information.pdf

Supporting Information

Genetic code expansion facilitates position-selective labeling of RNA for biophysical studies

Andreas Hegelein, Diana Müller, Sylvester Größl, Michael Göbel, Martin Hengesbach, and Harald Schwalbe

Content

In vitro transcription and NMR of RNA 4

Figure S1: Denaturing polyacrylamide gel of 14mer and 14mer^X for analysis of the transcription efficiency 4

Table S1: Analysis of the relative yield of 14mer^X to 14mer based on the gel shown in Figure 1 done with ImageJ. 5

Figure S2: 1D 1H Imino region of 14mer and 14mer^X at 278 K. 6

Figure S3: Optimization of XTP concentration for *in vitro* transcription of G79X. 7

Table S2: Analysis of denaturing polyacrylamide gels (Figure 5B) to determine the absolute and relative transcription yield of G79X (73mer) and abortion product G79X abortion (67mer). 8

Figure S4: Optimization of 7-deazaxanthosine concentration for *in vitro* transcription of G79-7dX. 9

Table S3: Analysis of denaturing polyacrylamide gel (Figure 6A) to determine the absolute and relative transcription yield of G79-7dX (73mer) and abortion product G79-7dX abortion (67mer). 10

Analytics of 3′,5′-Bis-*O*-(*tert*-butyldimethylsilyl)thymidine (2) 11

Figure S5: ^{1}H-spectrum 11

Figure S6: ^{13}C-spectrum 12

Figure S7: MALDI-spectrum 13

Analytics of 1,4-anhydro-3,5-bis-*O*-(*tert*-butyldimethylsilyl)-2-deoxy-D-erythro-pent-1-enitol (3) . 14

Figure S8: ^{1}H-spectrum 14

Figure S9: ^{13}C-spectrum 15

Figure S10: MALDI-spectrum 16

Analytics of 3-*O*-(*tert*-butyldimethylsilyl)-1,2-dideoxy-2,3-didehydro-D-ribofuranose (4) 17

Figure S11: ^{1}H-spectrum 17

Figure S12: ^{13}C-spectrum 18

Figure S13: MALDI-spectrum 19

Analytics of 1*β*-(2,4-dichloropyrimidin-5-yl)-1,2,3-trideoxy-3-oxo-D-ribofuranose (6) 20

Figure S14: ^{1}H-spectrum 20

Figure S15: ^{13}C-spectrum 21

Figure S16: MALDI-spectrum 22

Analytics of 1*β*-(2,4-dichloropyrimidin-5-yl)-1,2-dideoxy-D-ribofuranose (7) 23

Figure S17: ^{1}H-spectrum 23

Figure S18: ^{13}C-spectrum 24

Figure S19: MALDI-spectrum 25

Analytics of 1*β*-(2,4-dichloropyrimidin-5-yl)-1,2-dideoxy-5-*O*-(*tert*-butyl-dimethylsilyl)-D-ribofuranose (8) 26

Figure S20: ^{1}H-spectrum 26

Figure S21: ^{13}C-spectrum 27

Figure S22: MALDI-spectrum 28

Analytics of 1*β*-[2,4-bis(benzoylamino)pyrimidin-5-yl]-1,2-dideoxy-5-*O*-(*tert*-butyl-dimethylsilyl)-D-ribofuranose (9) 29

Figure S23: ^{1}H-spectrum 29

Figure S24: ^{13}C-spectrum 30

Figure S25: MALDI-spectrum 31

Analytics of 1*β*-[2,4-bis(benzoylamino)pyrimidin-5-yl]-1,2-dideoxy-D-ribofuranose (10) 32

Figure S26: ^{1}H-spectrum 32

Figure S27: ^{13}C-spectrum 33

Figure S28: MALDI-spectrum 34

Analytics of 1*β*-[2,4-bis(benzoylamino)pyrimidin-5-yl]-1,2-dideoxy-5-*O*-(4,4'-dimethoxytriphenylmethyl)-D-ribofuranose (11) 35

Figure S29: ^{1}H-spectrum 35

Figure S30: ^{13}C-spectrum 36

Figure S31: MALDI-spectrum 37

Analytics of 1*β*-[2,4-bis(benzoylamino)pyrimidin-5-yl]-1,2-dideoxy-5-*O*-(4,4'-dimethoxytriphenylmethyl)-D-ribofuranose-3-[(2-cyanoethyl)(*N,N*-diisopropyl)]phosphoramidite (12) 38

Figure S32: ^{1}H-spectrum 38

Figure S33: ^{31}P-spectrum 39

Figure S34: MALDI-spectrum 40

Analytics of *N*-(4-chloro-7*H*-pyrrolo[2,3-d]pyrimidin-2-yl)-2,2-dimethyl-propionamide (14) 41

Figure S35: ^{1}H-spectrum 41

Figure S36: ^{13}C-spectrum 42

Figure S37: ESI-spectrum 43

Analytics of *N*-(4-chloro-5-iodo-7*H*-pyrrolo[2,3-d]pyrimidin-2-yl)-2,2-dimethyl-propionamide (15) 44

Figure S38: ^{1}H-spectrum 44

Figure S39: ^{13}C-spectrum 45
Figure S40: ESI-spectrum 46
Analytics of 4-chloro-5-iodo-2-pivaloylamino-7-[(2,3,5-tri-*O*-benzoyl)-*β*-D-ribofuranosyl]-7*H*-pyrrolo[2,3-d]pyrimidine (16) 47
Figure S41: ^{1}H-spectrum 47
Figure S42: ^{13}C-spectrum 48
Figure S43: ESI-spectrum 49
Analytics of 2-amino-5-iodo-3,7-dihydro-7-(*β*-D-ribofuranosyl)-4*H*-pyrrolo-[2,3-d]pyrimidin-4-one (17) 50
Figure S44: ^{1}H-spectrum 50
Figure S45: ^{13}C-spectrum 51
Figure S46: ESI-spectrum 52
Analytics of 1,7-dihydro-5-iodo-4-methoxy-7-(*β*-D-ribofuranosyl)-2*H*-pyrrolo[2,3-d]pyrimidin-2-amine (18) 53
Figure S47: ^{1}H-spectrum 53
Figure S48: ^{13}C-spectrum 54
Figure S49: ESI-spectrum 55
Analytics of 1,7-dihydro-5-(octa-1,7-diynyl)-4-methoxy-7-(*β*-D-ribofuranosyl)-2*H*-pyrrolo[2,3-d]pyrimidin-2-amine (19) 56
Figure S50: ^{1}H-spectrum 56
Figure S51: 13C-spectrum 57
Figure S52: ESI-spectrum 58
Analytics of 5-(octa-1,7-diynyl)-7-(*β*-D-ribofuranosyl)-1,3,7-trihydro-2*H*,4*H*-pyrrolo-[2,3-d]pyrimidin-2,4-dione (20) 59
Figure S53: ^{1}H-spectrum 59
Figure S54: ^{13}C-spectrum 60
Figure S55: ESI-spectrum 61
Analytics of 5-(octa-1,7-diynyl)-7-(*β*-D-ribofuranosyl)-1,3,7-trihydro-2*H*,4*H*-pyrrolo-[2,3-d]pyrimidin-2,4-dione TP (21) 62
Figure S56: ^{1}H-spectrum 62
Figure S57: ^{31}P-spectrum 63
Figure S58: ^{31}P-HMBC-spectrum 64
Figure S59: MALDI-spectrum 65

In vitro transcription and NMR of RNA

Figure S1: Denaturing polyacrylamide gel of 14mer and 14mer^{X} for analysis of the transcription efficiency

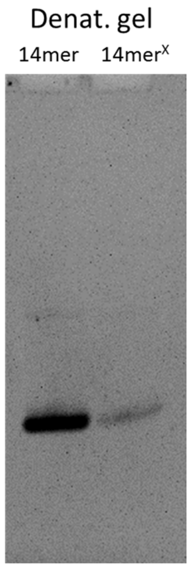

Table S1: Analysis of the relative yield of $14mer^X$ to 14mer based on the gel shown in Figure 1 done with ImageJ.

Lane	Band No.	Rel. Quant.	Band%
14mer	1	1	100
14merX	1	0,13010668	100

Figure S2: 1D 1H Imino region of 14mer and 14mer^{X} at 278 K.

The partial assignment was has been transferred from Fürtig et al.[31]

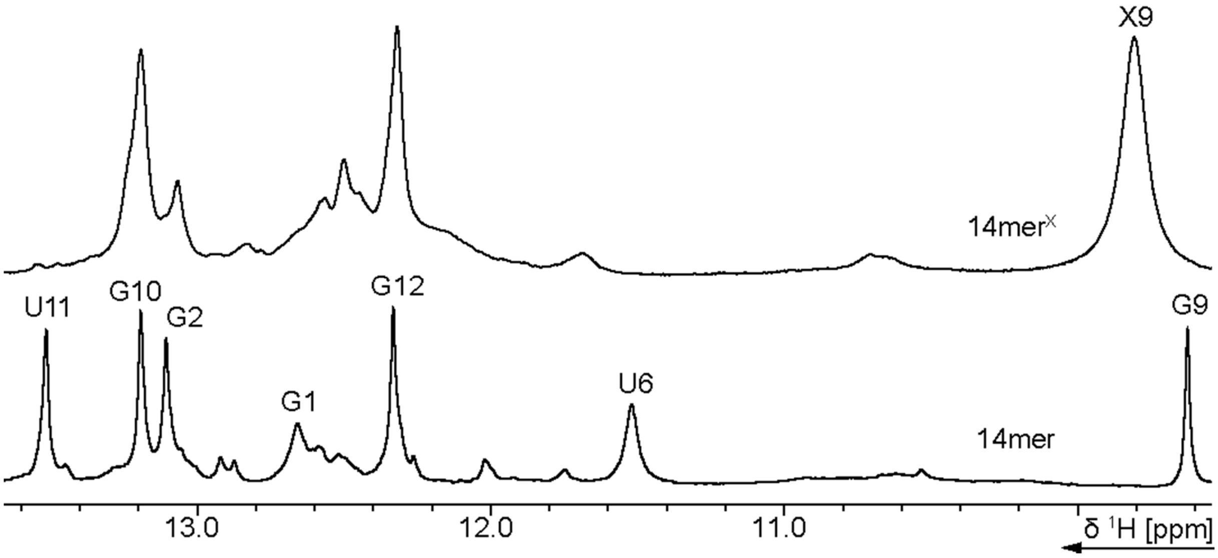

Figure S3: Optimization of XTP concentration for *in vitro* transcription of G79X.

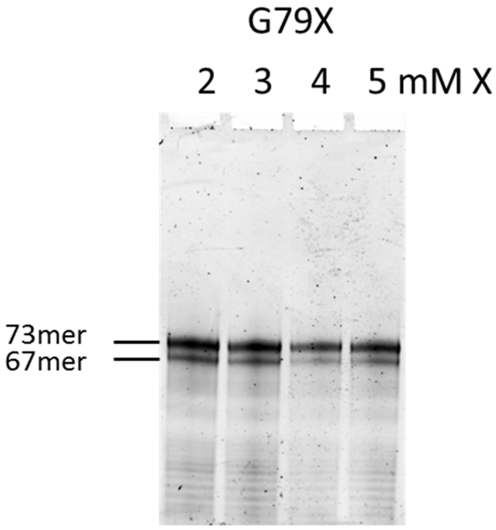

Table S2: Analysis of denaturing polyacrylamide gels (Figure 5B) to determine the absolute and relative transcription yield of G79X (73mer) and abortion product G79X abortion (67mer).

	Lane	Lane #	Band No.	Length	Rel. Quant.	Band %
	Gsw^{73}	1	1	73mer	1	100
-XTP	G79X	2	1	73mer	0,17	21,62
	G79X abortion		2	67mer	0,62	78,38
+XTP	G79X	3	1	73mer	0,71	76,73
	G79X abortion		2	67mer	0,22	23,27

Figure 5B for reference:

B

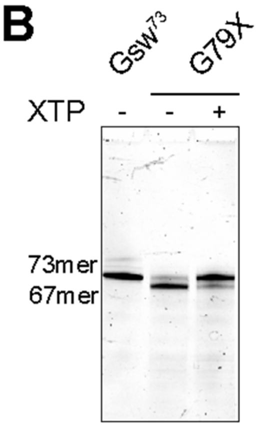

Figure S4: Optimization of 7-deazaxanthosine concentration for *in vitro* transcription of G79-7dX.

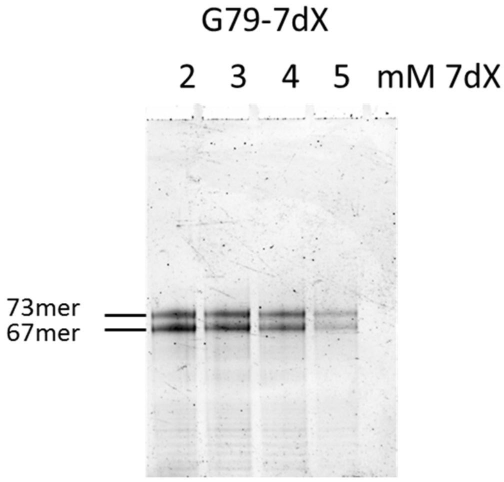

Table S3: Analysis of denaturing polyacrylamide gel (Figure 6A) to determine the absolute and relative transcription yield of G79-7dX (73mer) and abortion product G79-7dX abortion (67mer).

	Lane	Lane #	Band No.	Length	Rel. Quant.	Band %
	Gsw^{73}	1	1	73mer	1	100
-7dXTP	G79-7dX	2	1	73mer	0,16	19,16
	G79-7dX abortion		2	68mer	0,67	80,84
+7dXTP	G79-7dX	3	1	73mer	0,35	45,3
	G79-7dX abortion		2	68mer	0,42	54,7

Figure 6A for reference:

A

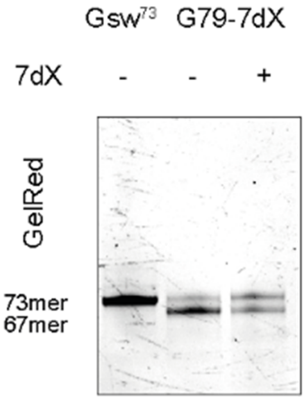

Analytics of 3′,5′-Bis-*O*-(*tert*-butyldimethylsilyl)thymidine (2)

Figure S5: ^{1}H-spectrum

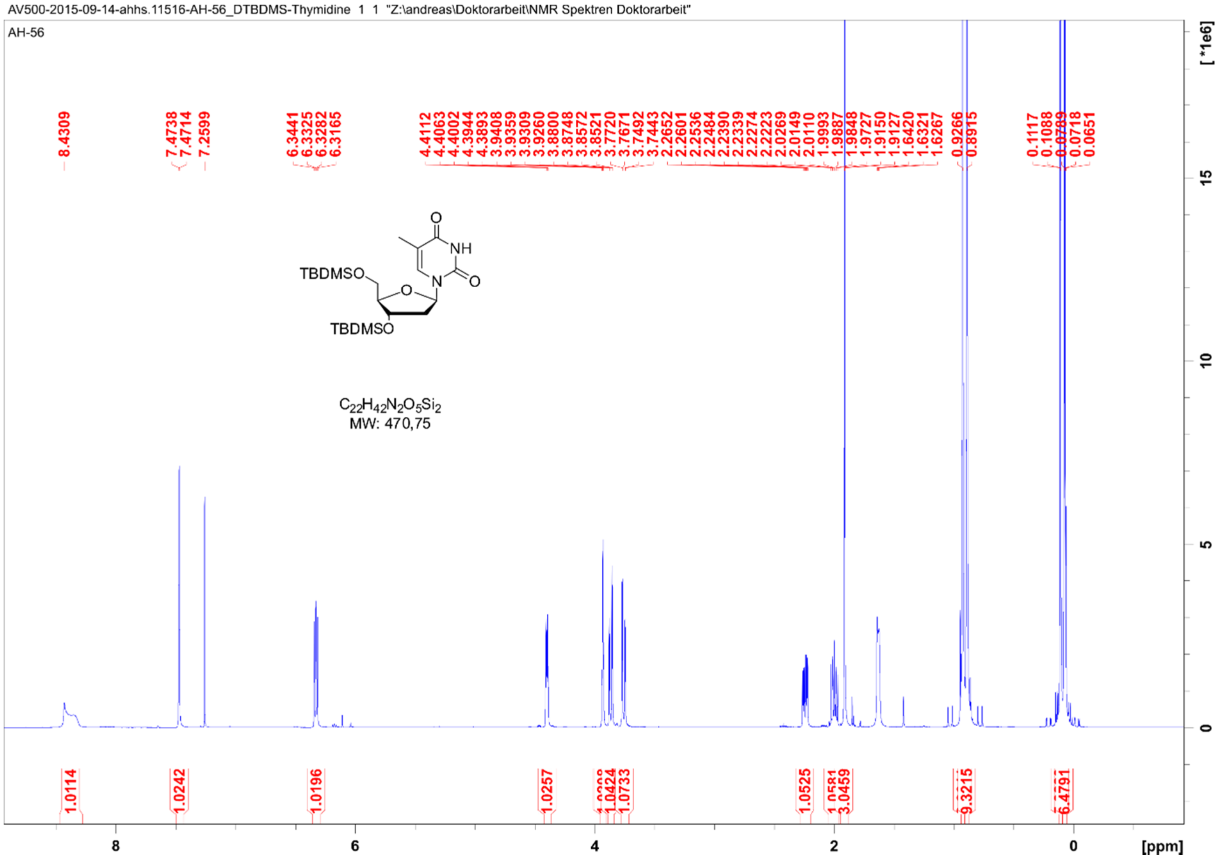

Figure S6: ^{13}C-spectrum

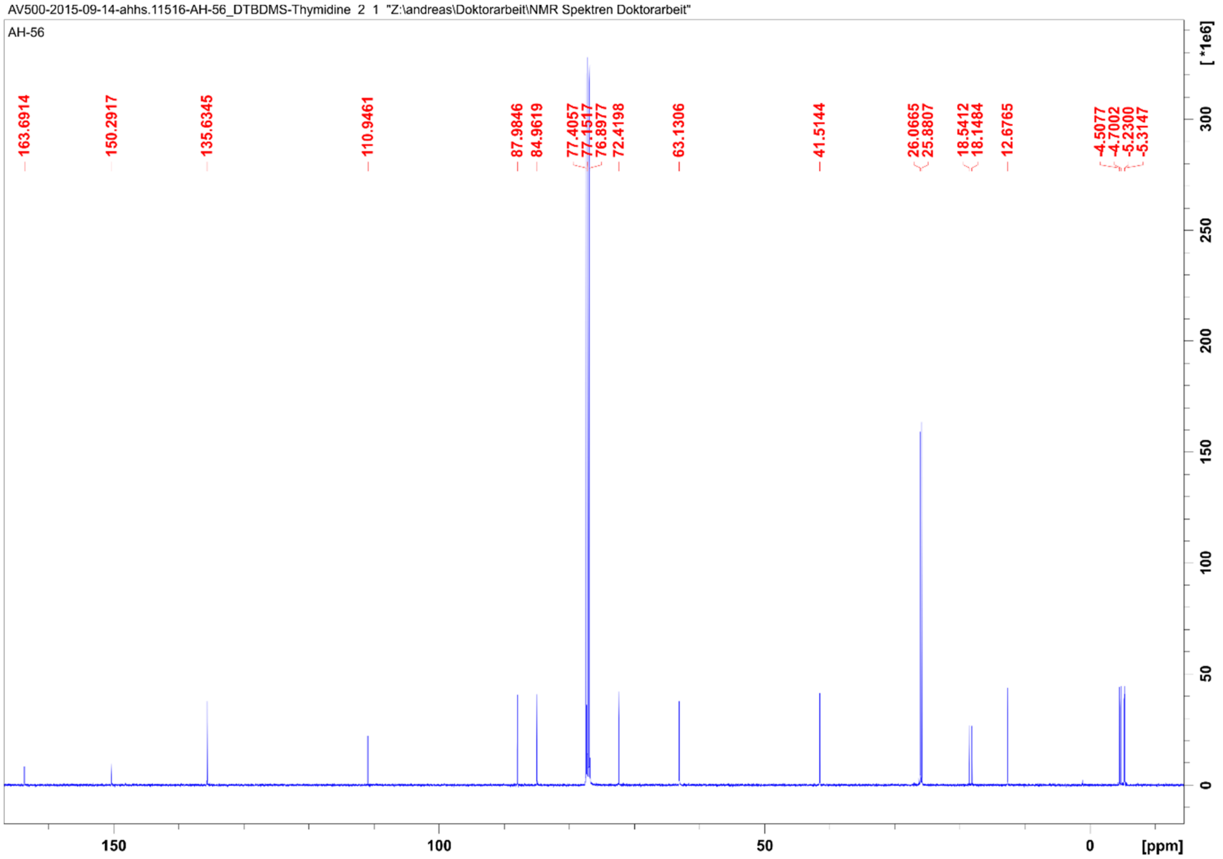

Figure S7: MALDI-spectrum

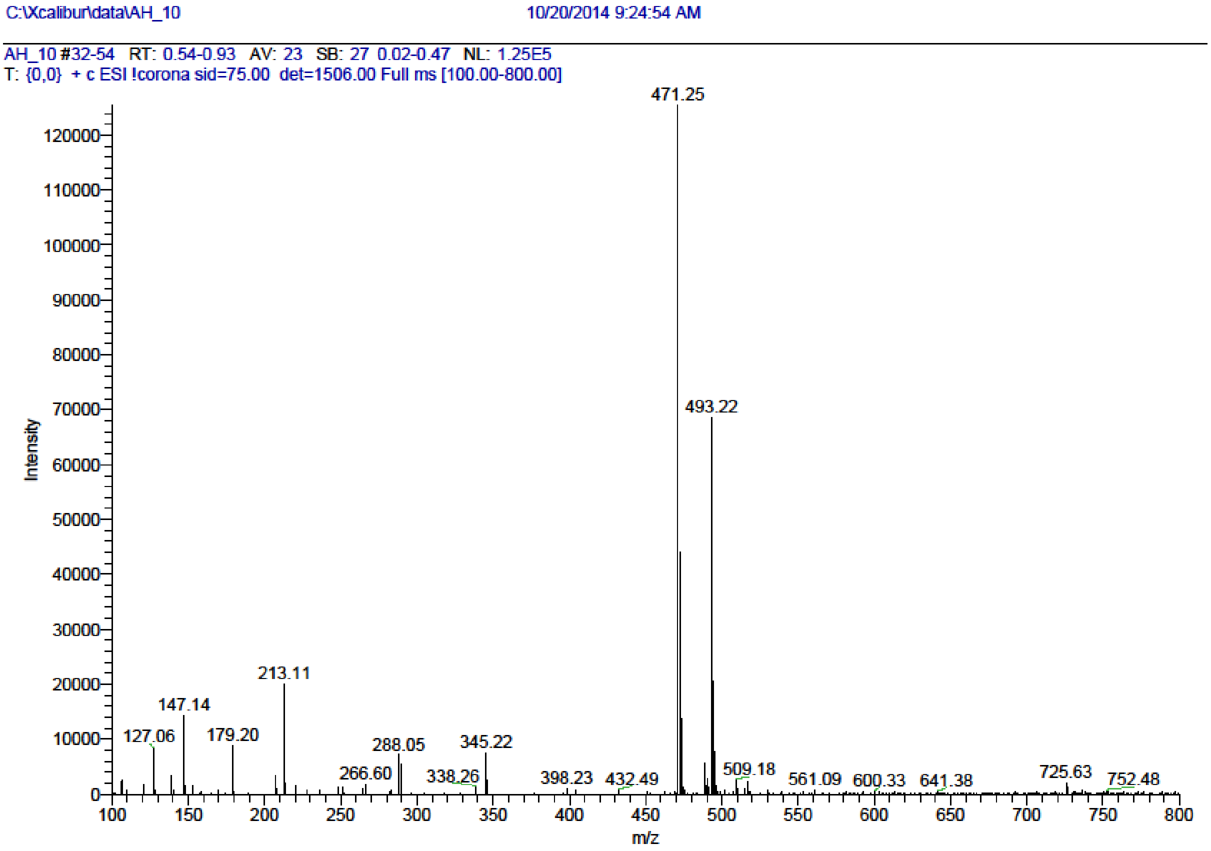

Analytics of 1,4-anhydro-3,5-bis-*O*-(*tert*-butyldimethylsilyl)-2-deoxy-D-erythro-pent-1-enitol (3)

Figure S8: ^{1}H-spectrum

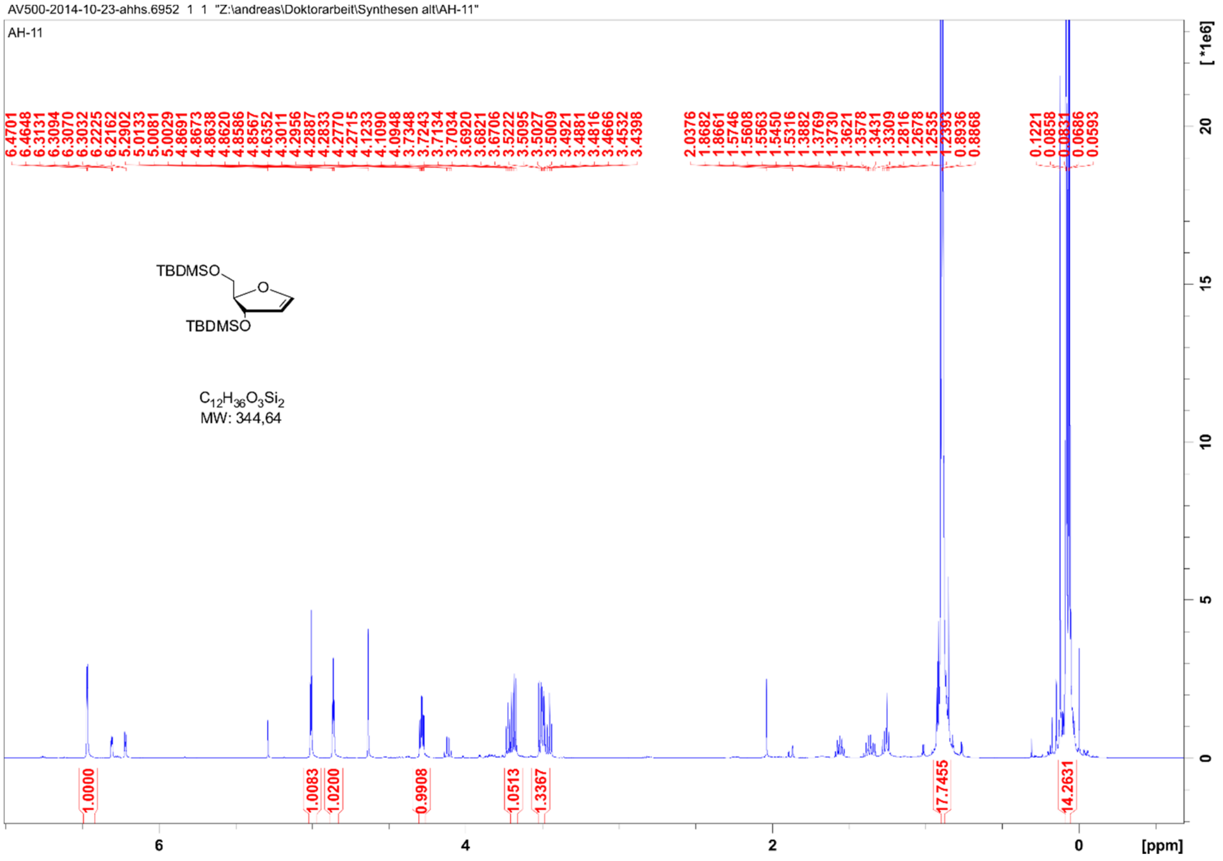

Figure S9: ^{13}C-spectrum

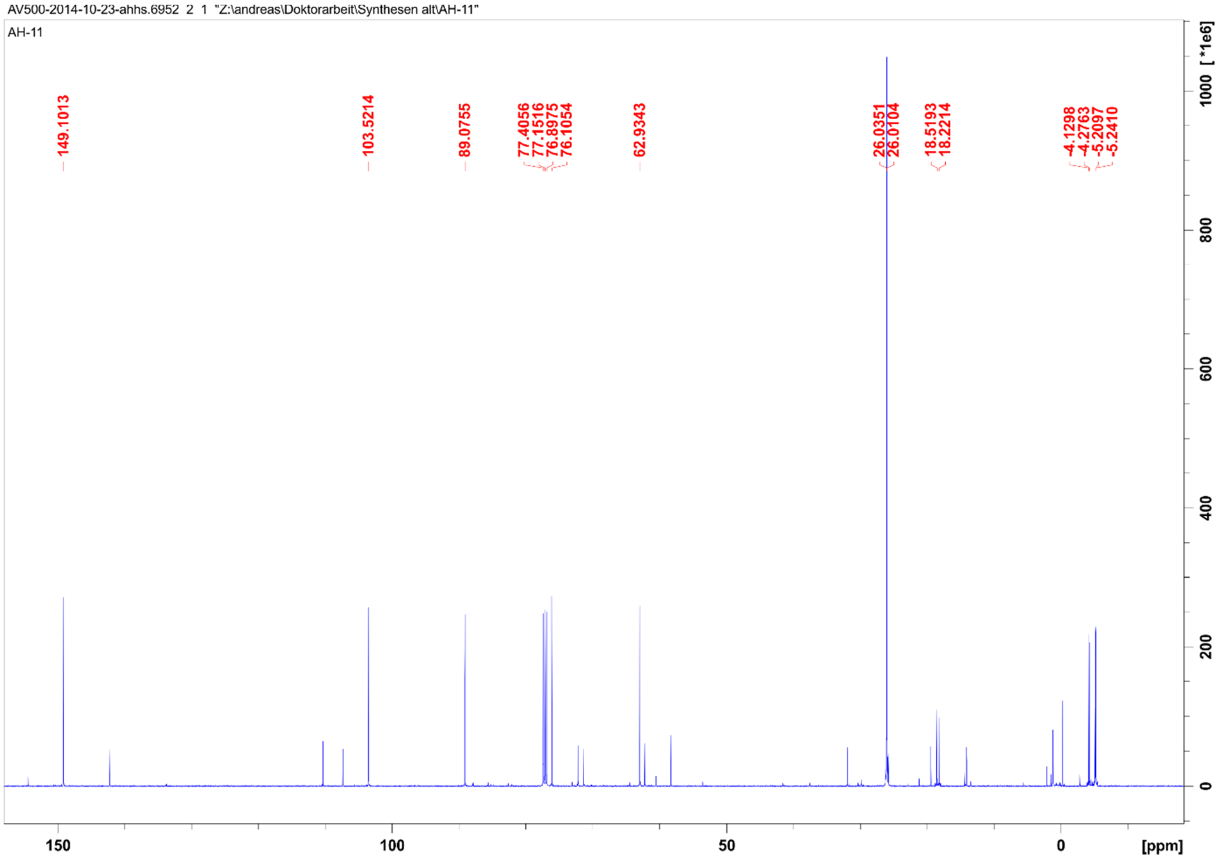

Figure S10: MALDI-spectrum

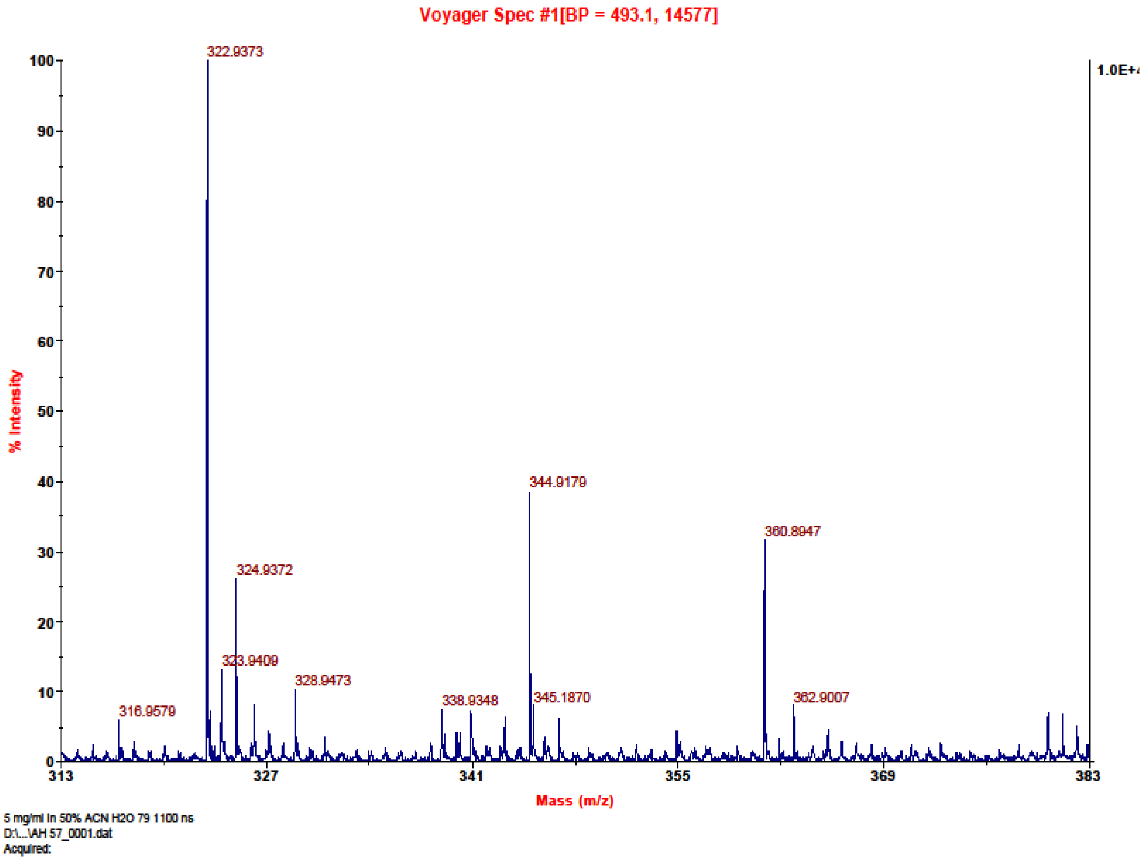

Analytics of 3-*O*-(*tert*-butyldimethylsilyl)-1,2-dideoxy-2,3-didehydro-D-ribofuranose (4)

Figure S11: ^{1}H-spectrum

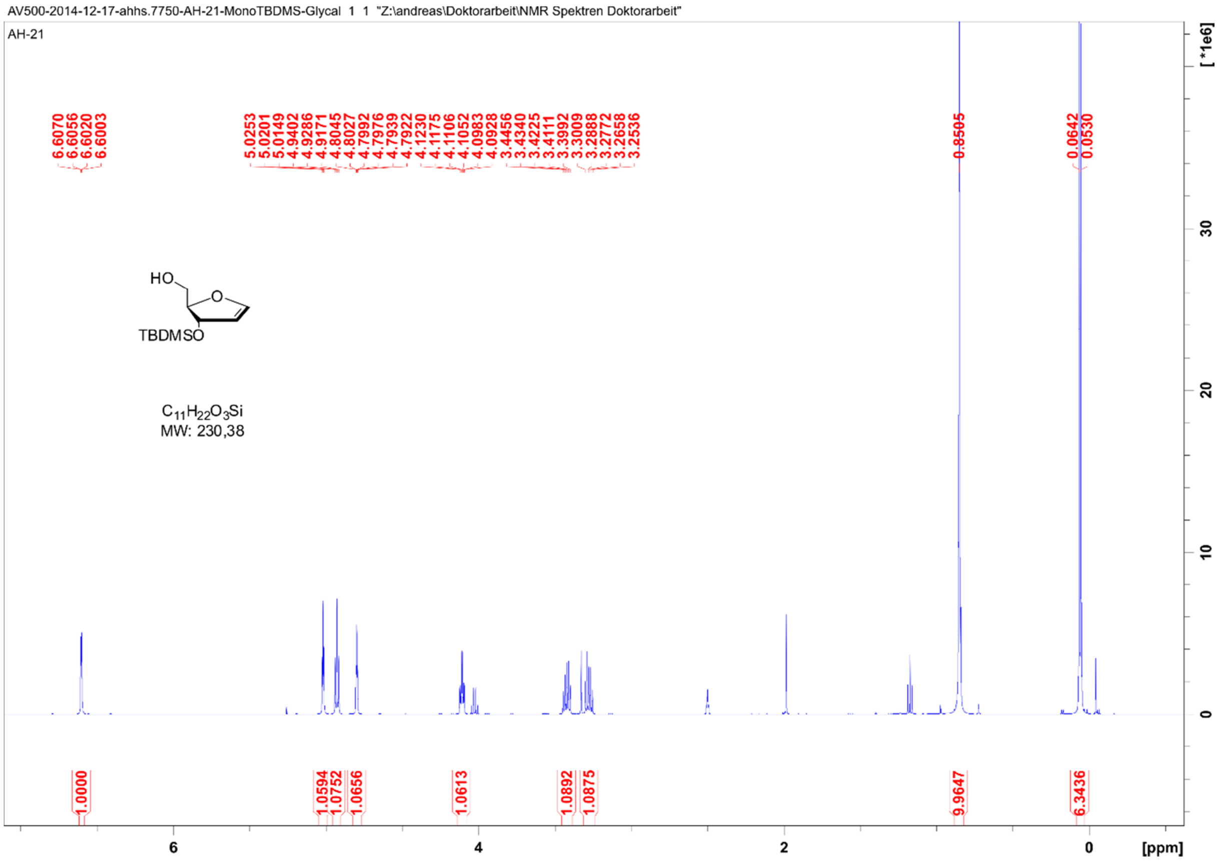

Figure S12: ^{13}C-spectrum

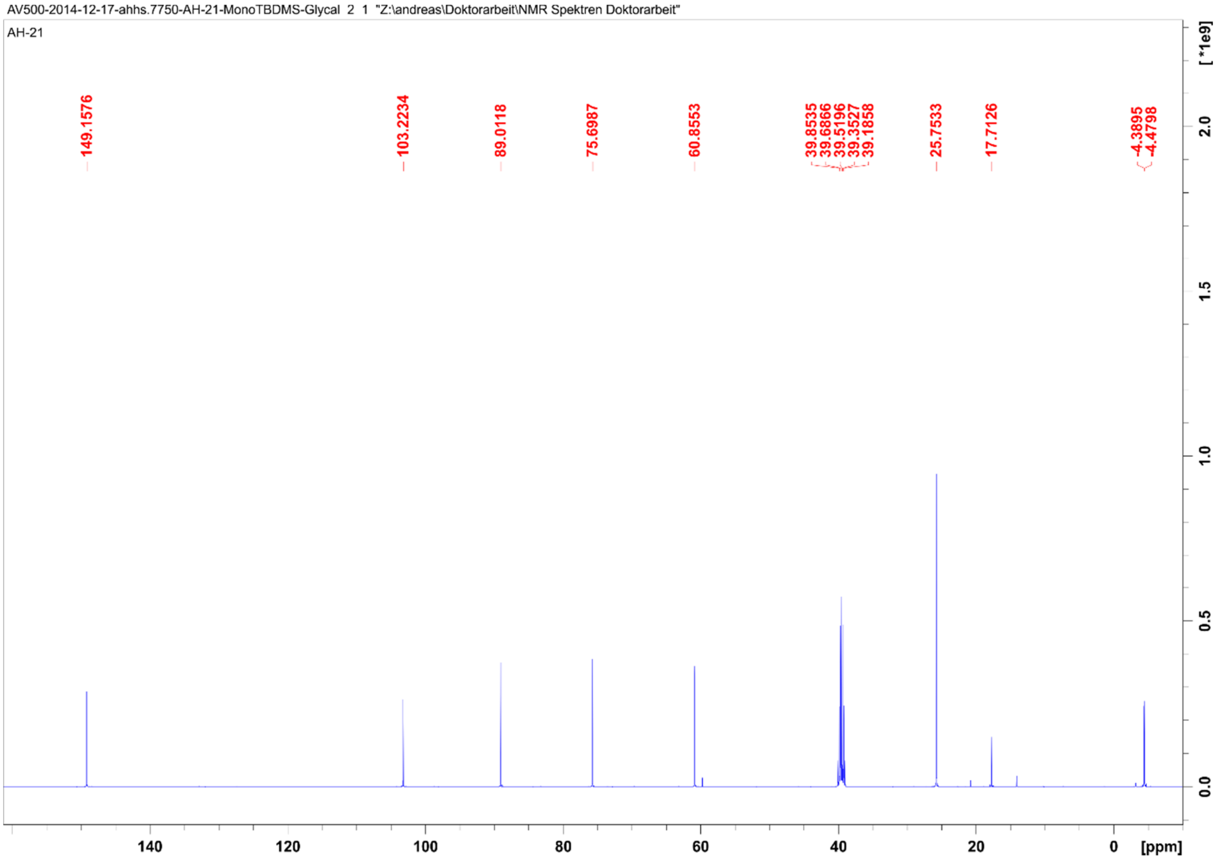

Figure S13: MALDI-spectrum

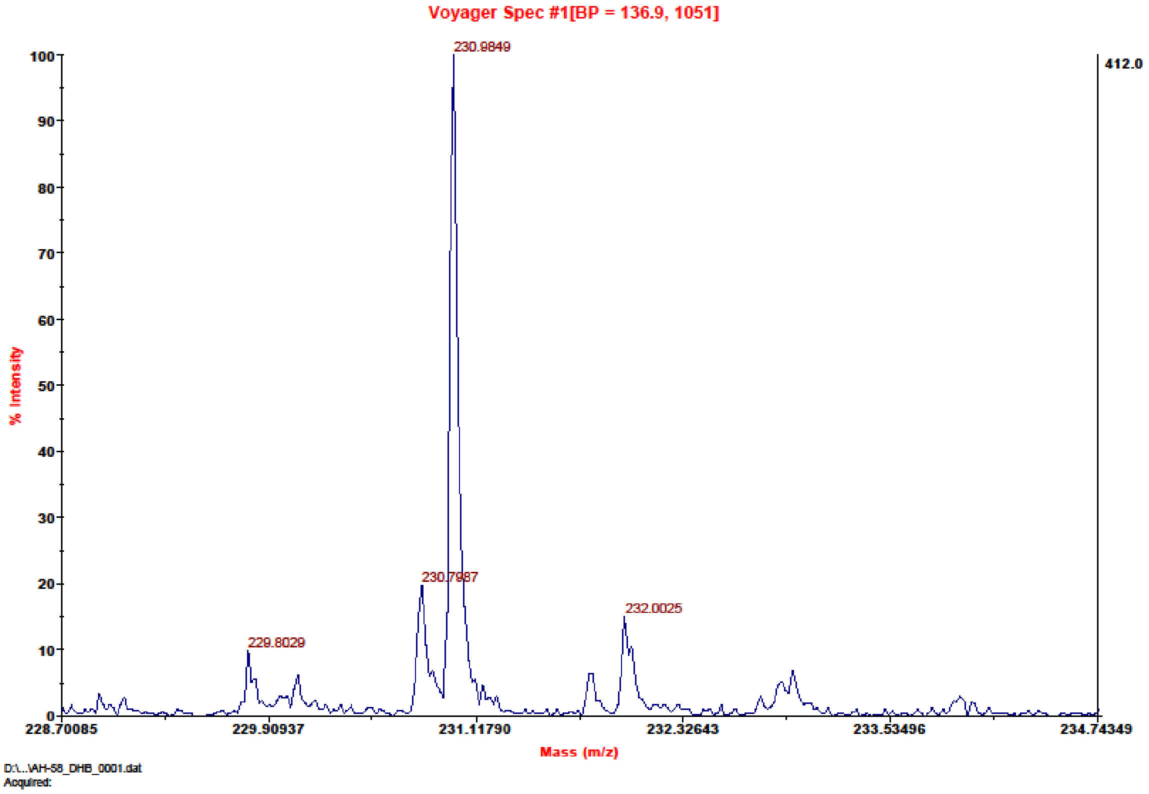

Analytics of 1β-(2,4-dichloropyrimidin-5-yl)-1,2,3-trideoxy-3-oxo-D-ribofuranose (6)

Figure S14: ^{1}H-spectrum

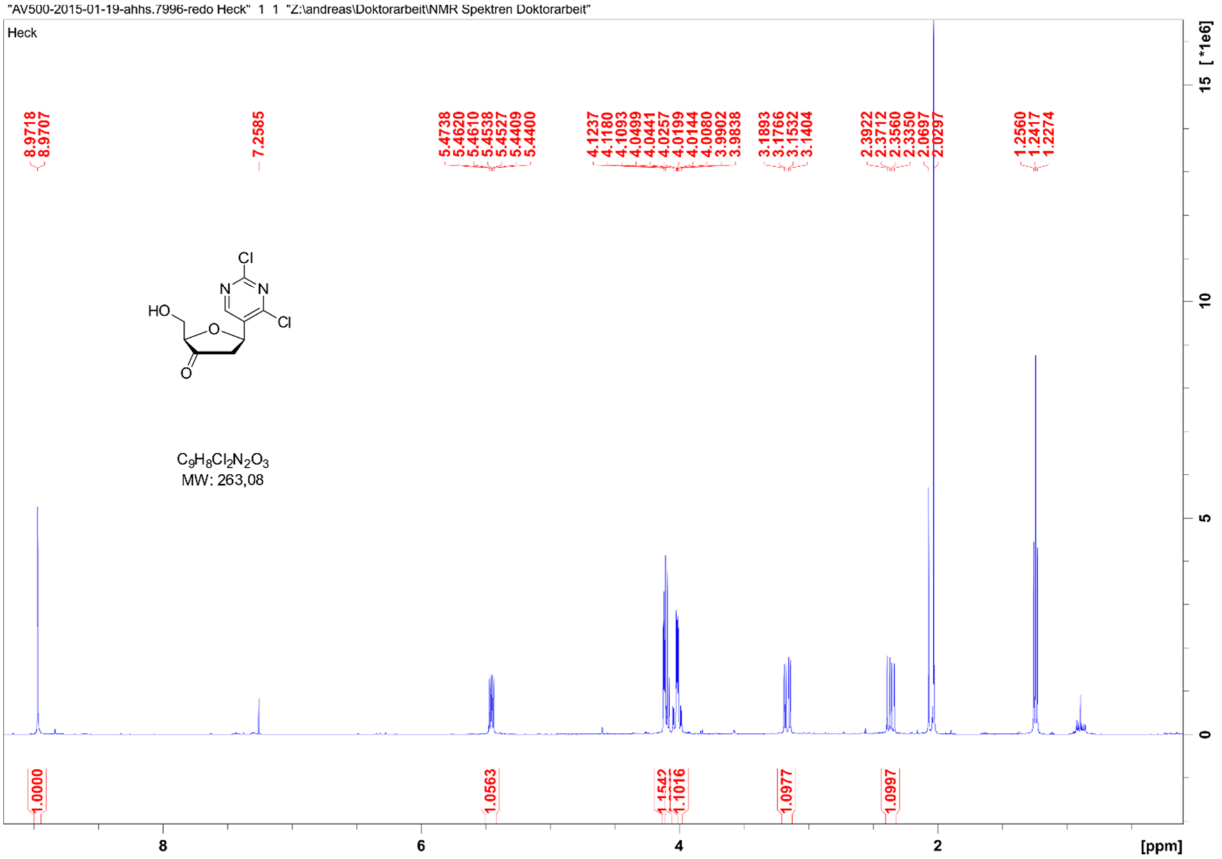

Figure S15: ^{13}C-spectrum

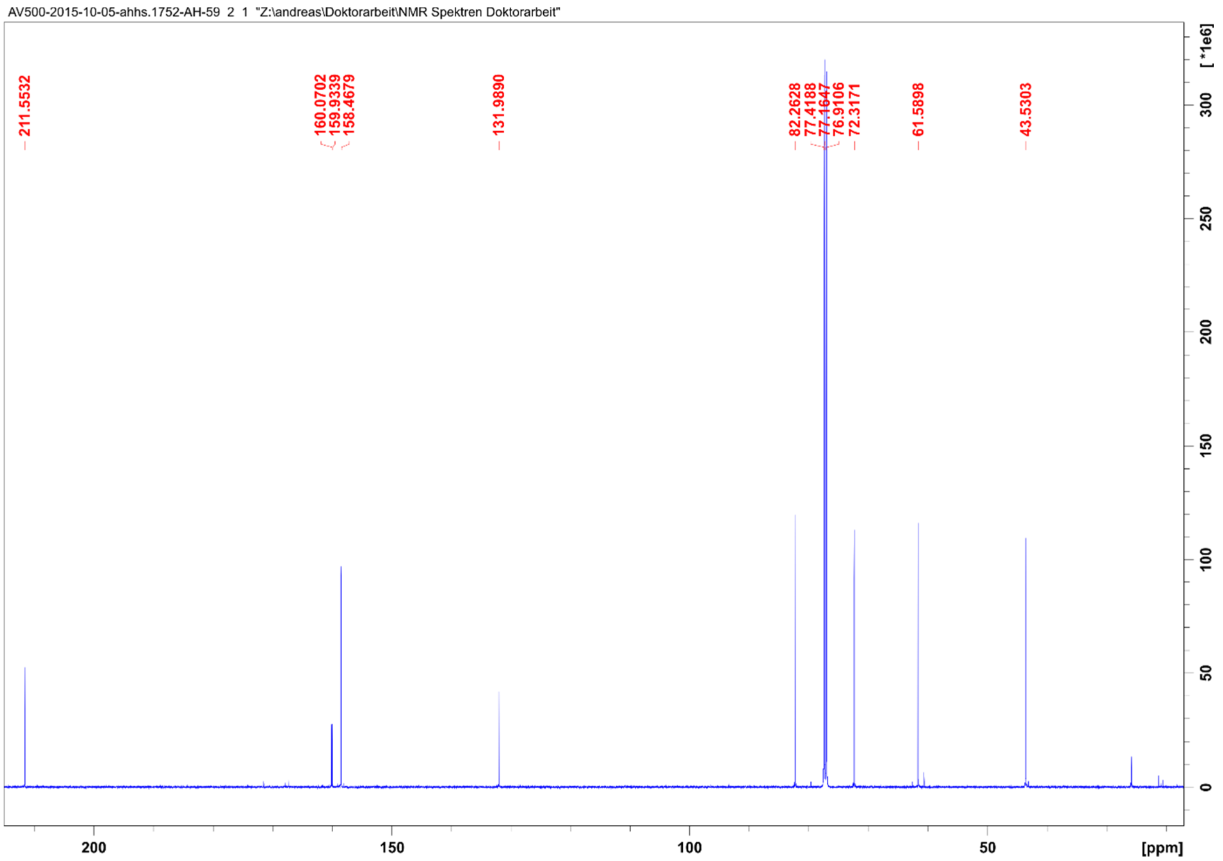

Figure S16: MALDI-spectrum

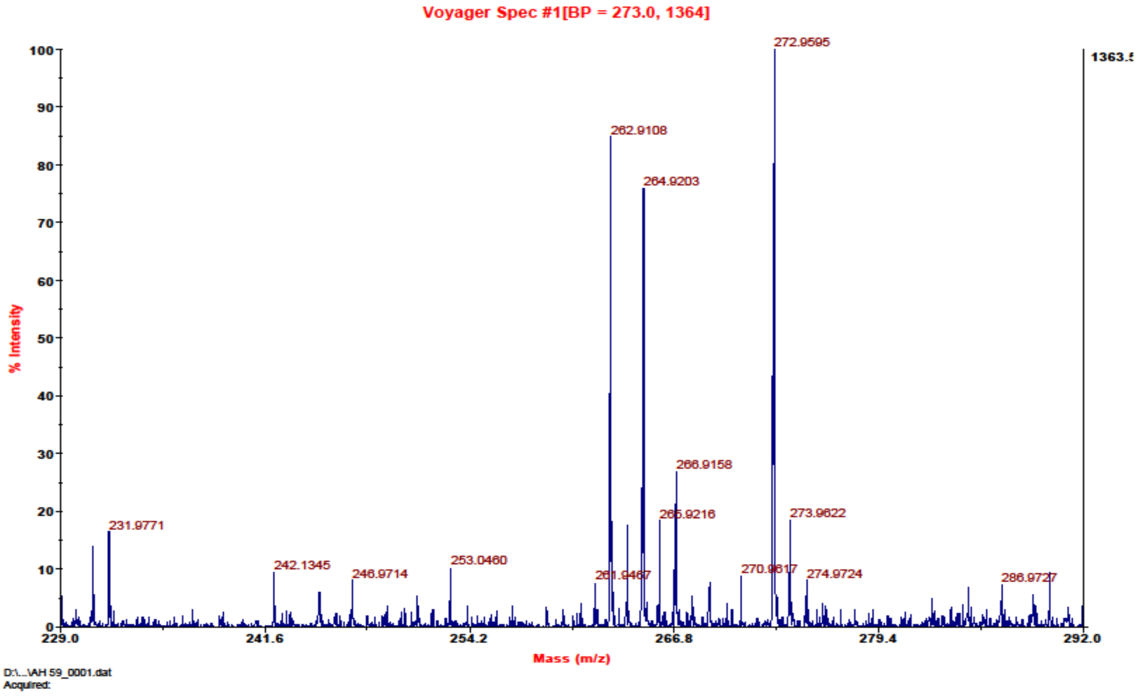

Analytics of 1β-(2,4-dichloropyrimidin-5-yl)-1,2-dideoxy-D-ribofuranose (7)

Figure S17: ^{1}H-spectrum

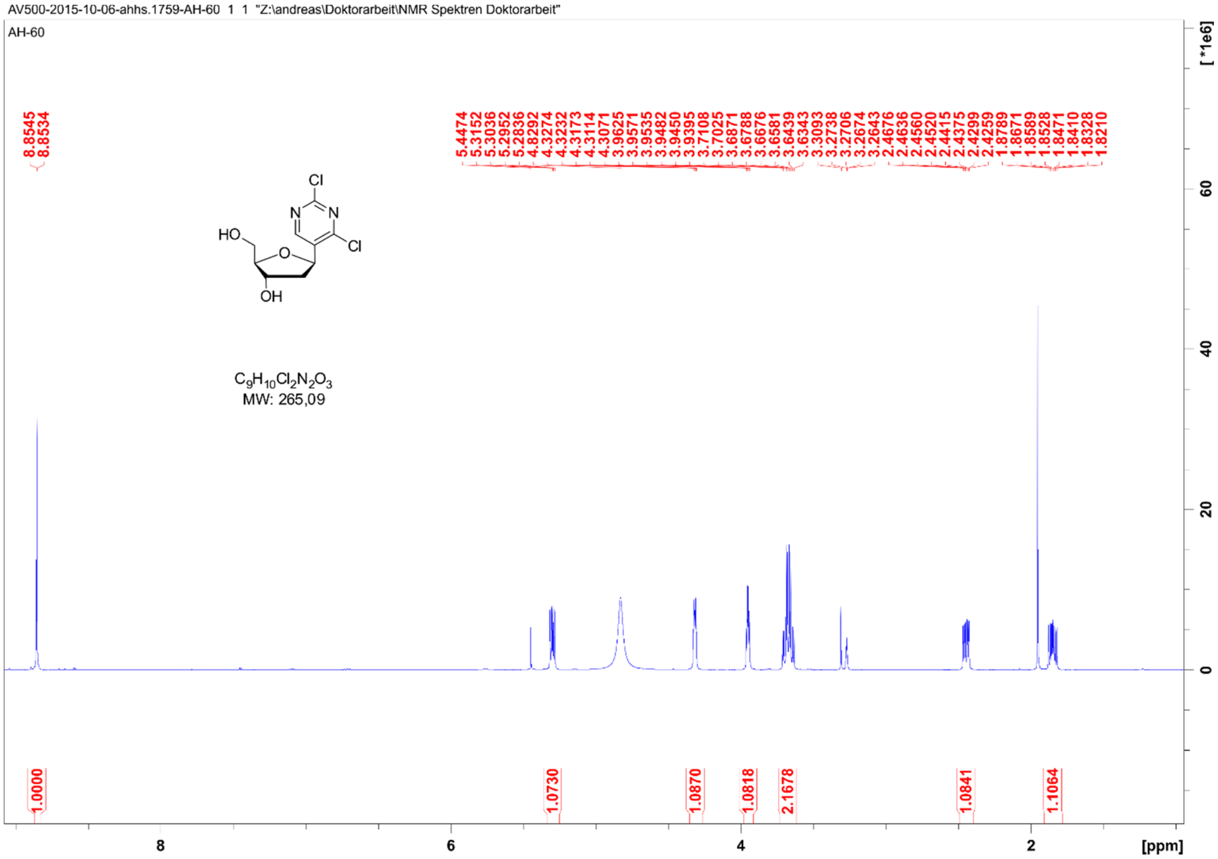

Figure S18: ^{13}C-spectrum

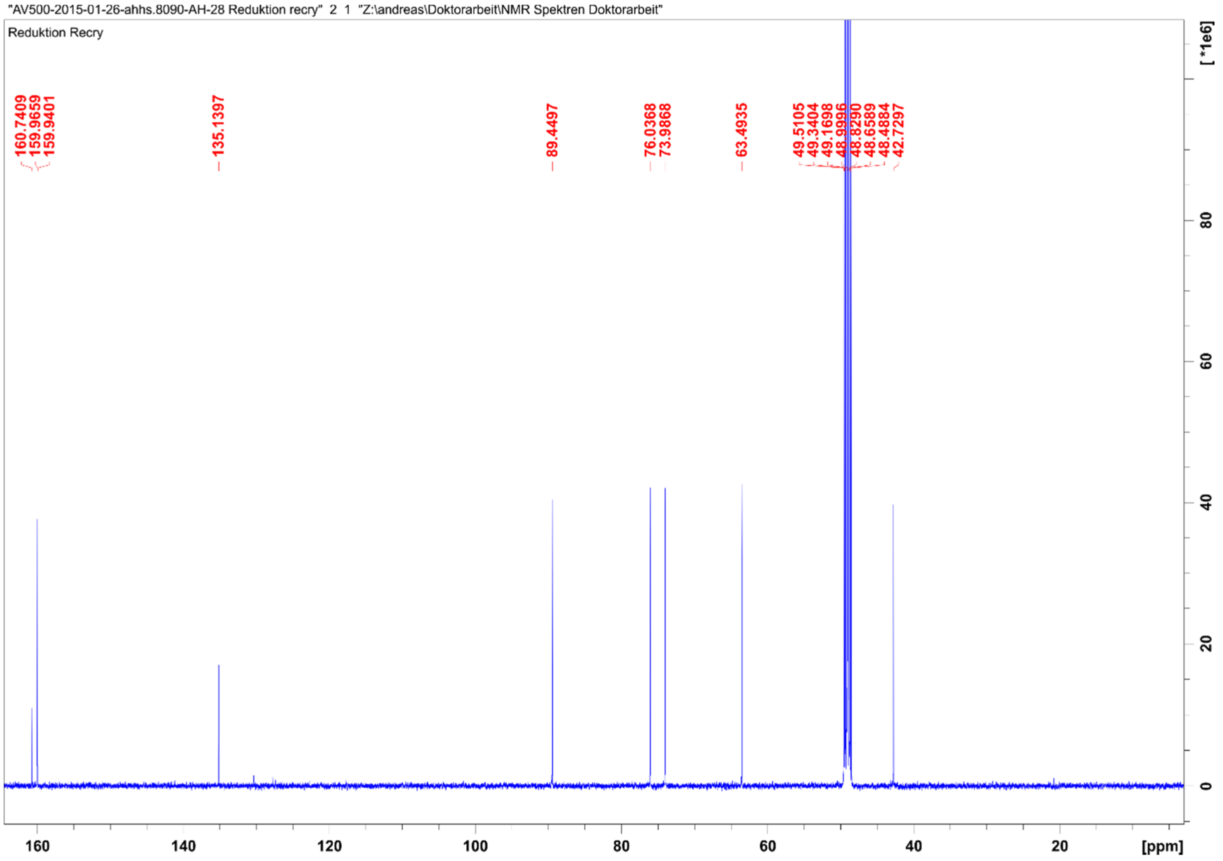

Figure S19: MALDI-spectrum

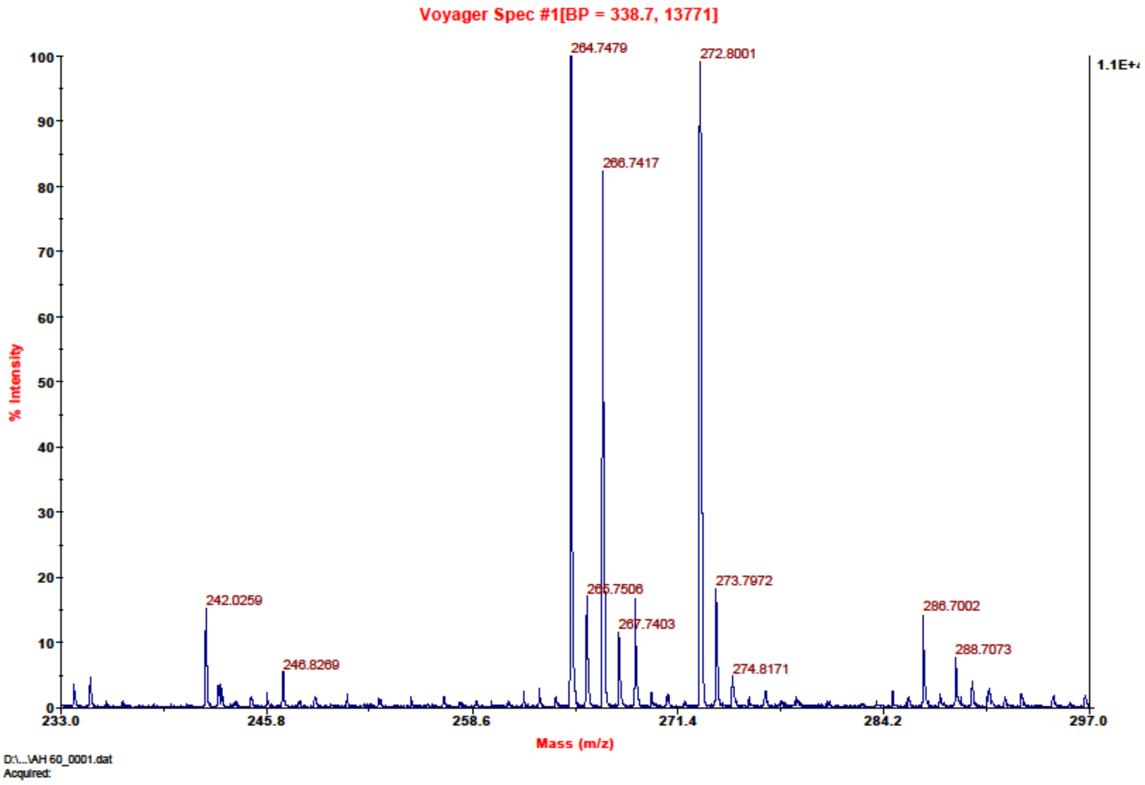

Analytics of 1*β*-(2,4-dichloropyrimidin-5-yl)-1,2-dideoxy-5-*O*-(*tert*-butyl-dimethylsilyl)-D-ribofuranose (8)

Figure S20: ^{1}H-spectrum

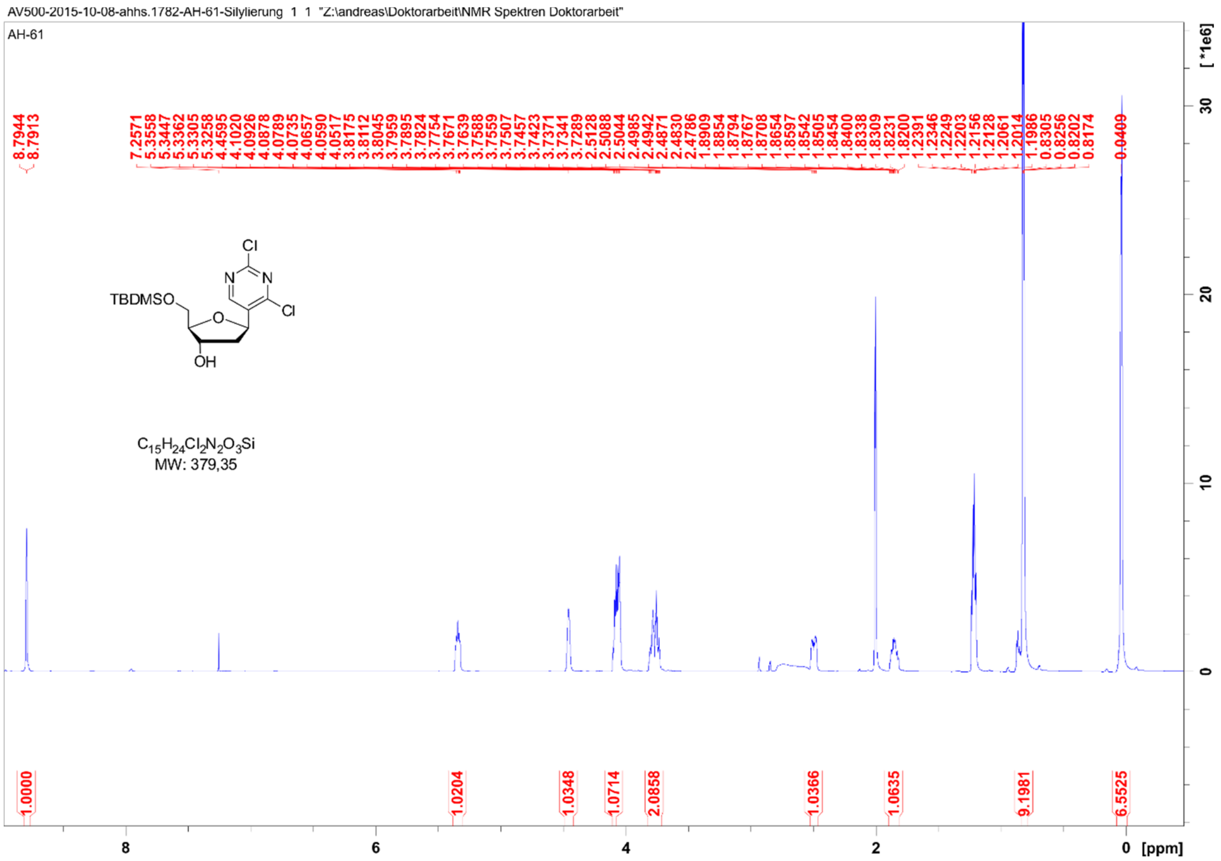

Figure S21: ^{13}C-spectrum

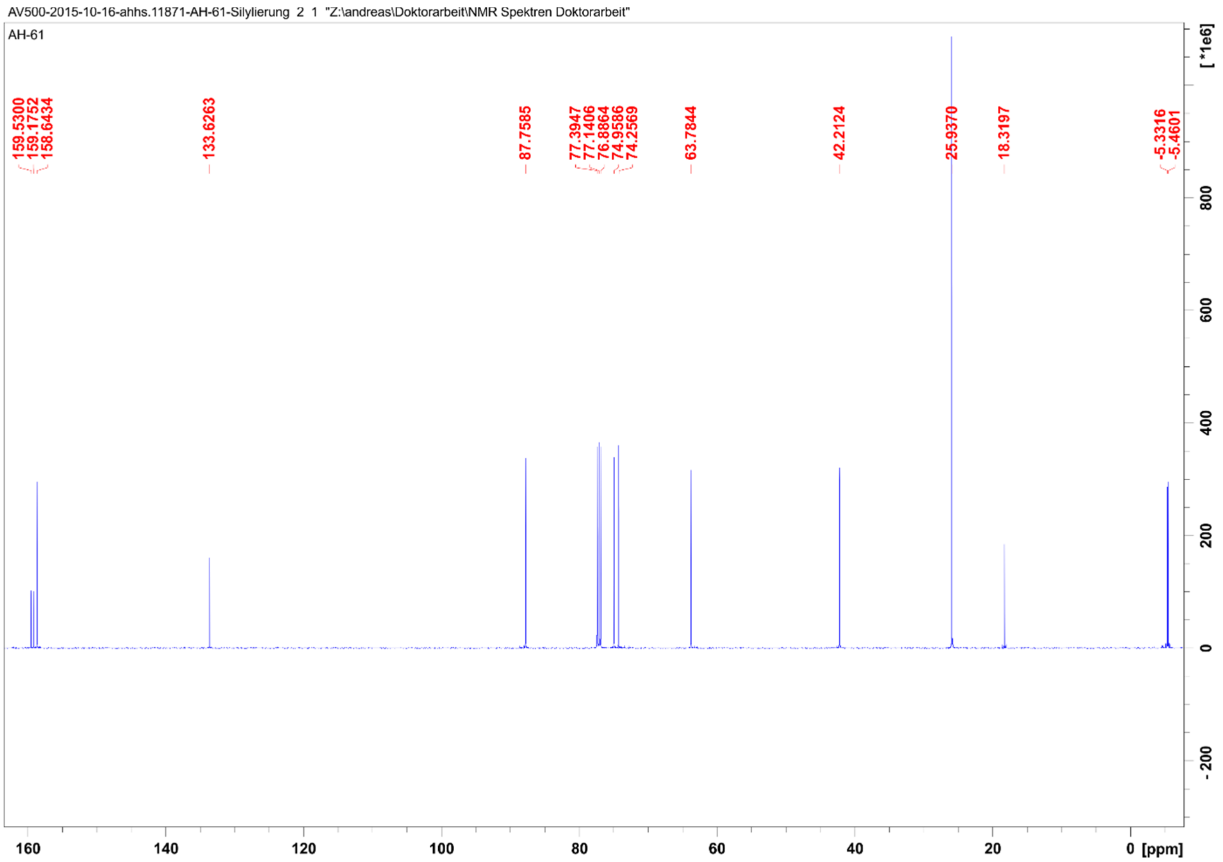

Figure S22: MALDI-spectrum

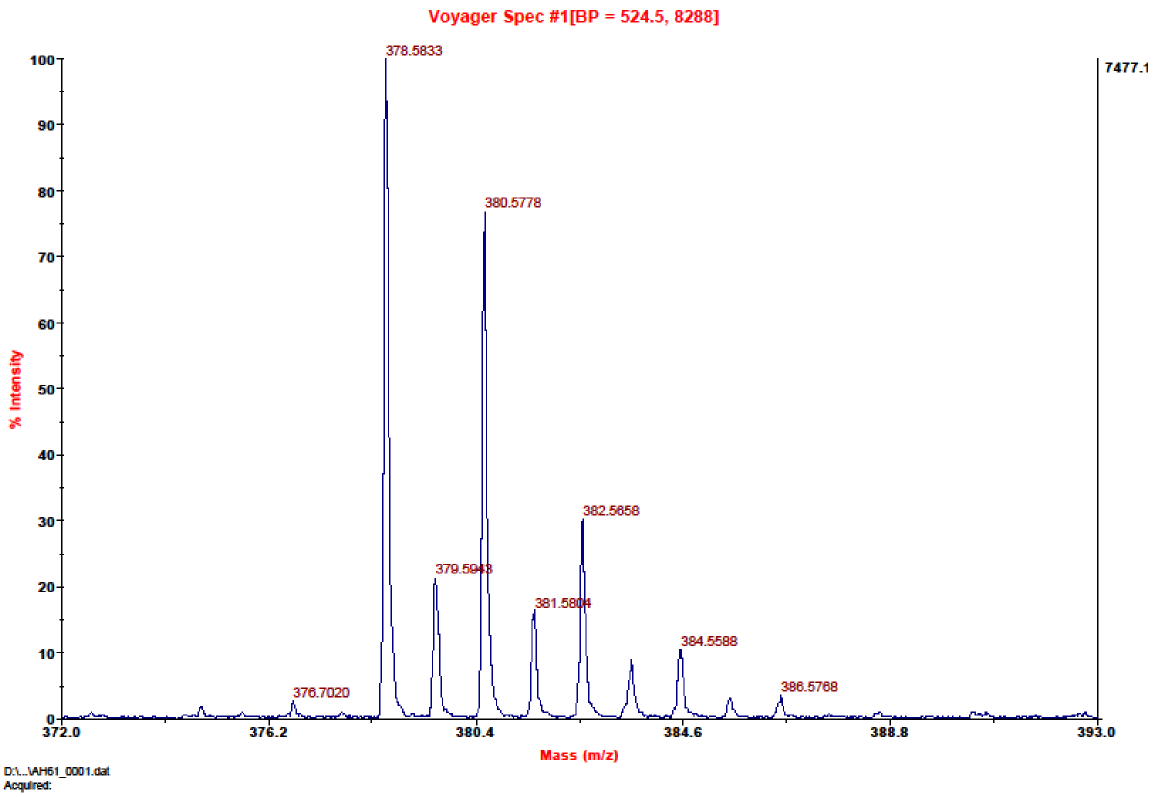

Analytics of 1β-[2,4-bis(benzoylamino)pyrimidin-5-yl]-1,2-dideoxy-5-*O*-(*tert*-butyl-dimethylsilyl)-D-ribofuranose (9)

Figure S23: ^{1}H-spectrum

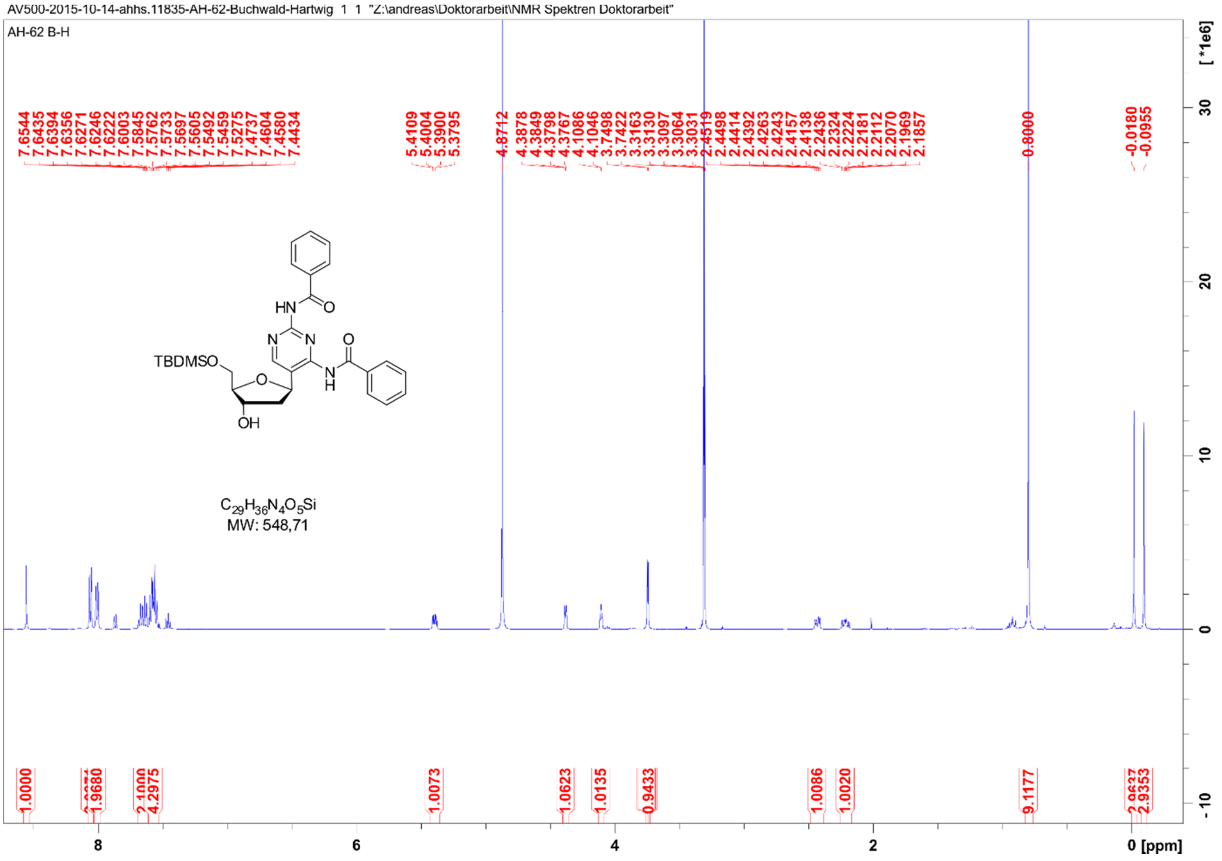

Figure S24: ^{13}C-spectrum

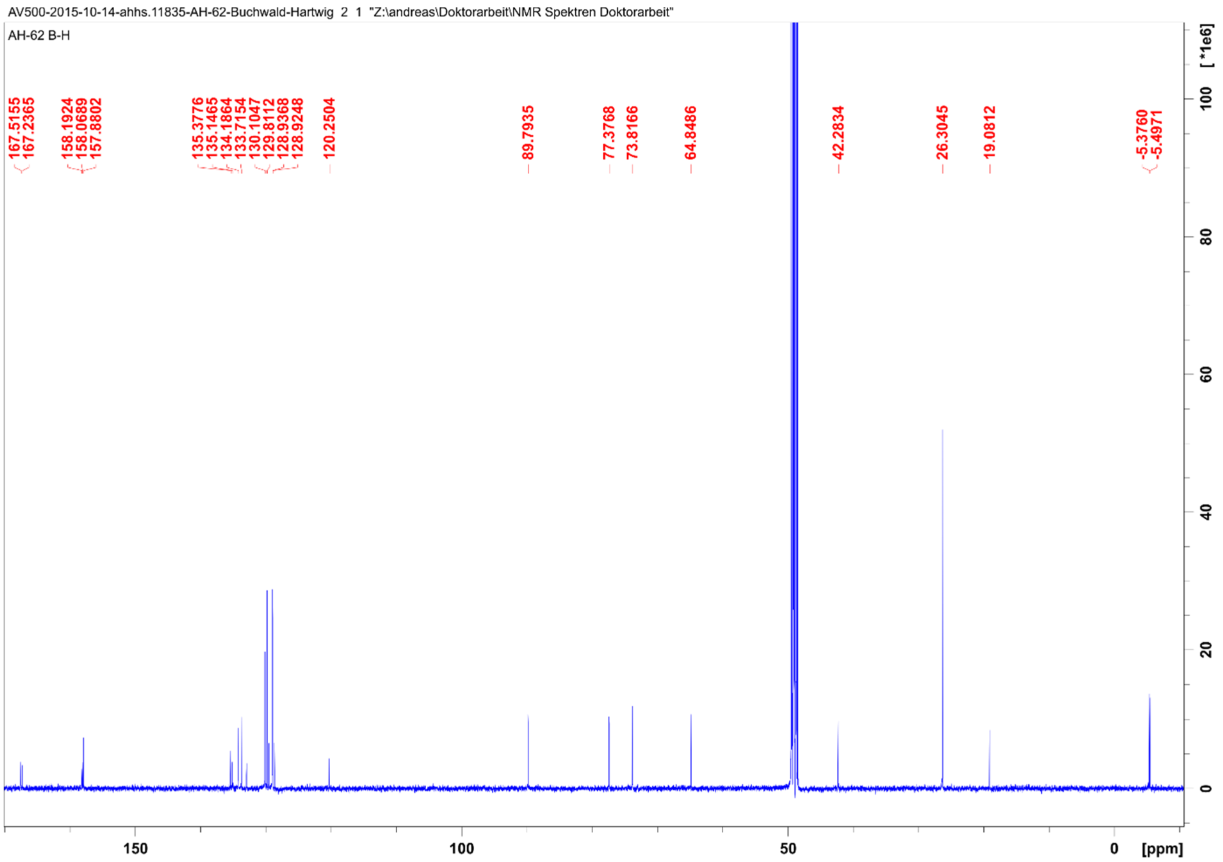

Figure S25: MALDI-spectrum

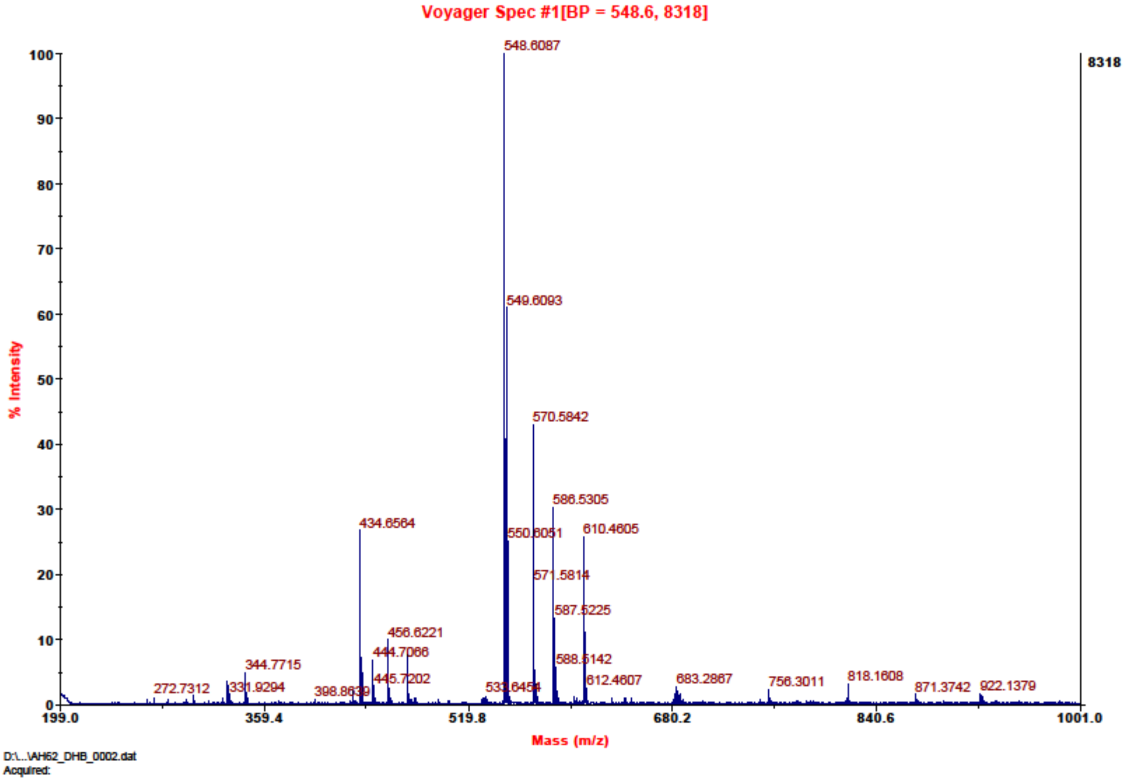

Analytics of 1β-[2,4-bis(benzoylamino)pyrimidin-5-yl]-1,2-dideoxy-D-ribofuranose (10)

Figure S26: ^{1}H-spectrum

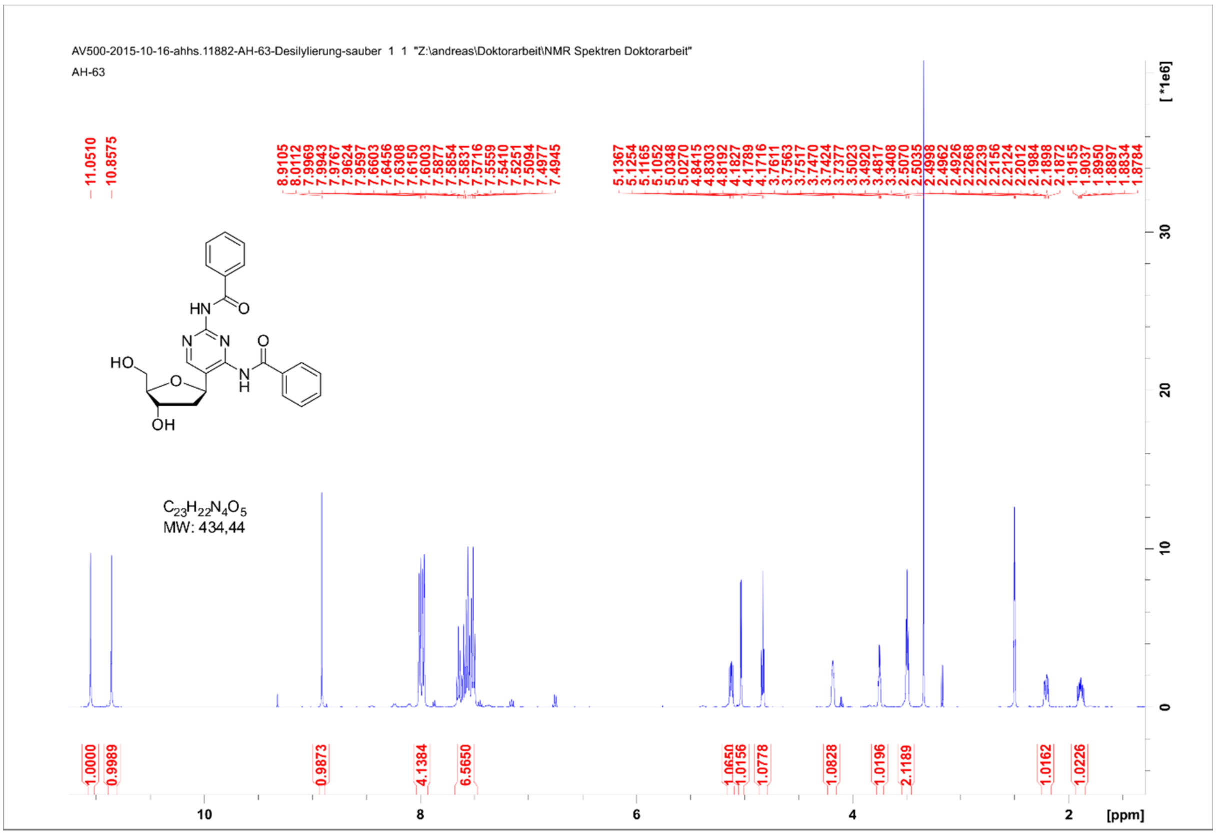

Figure S27: ^{13}C-spectrum

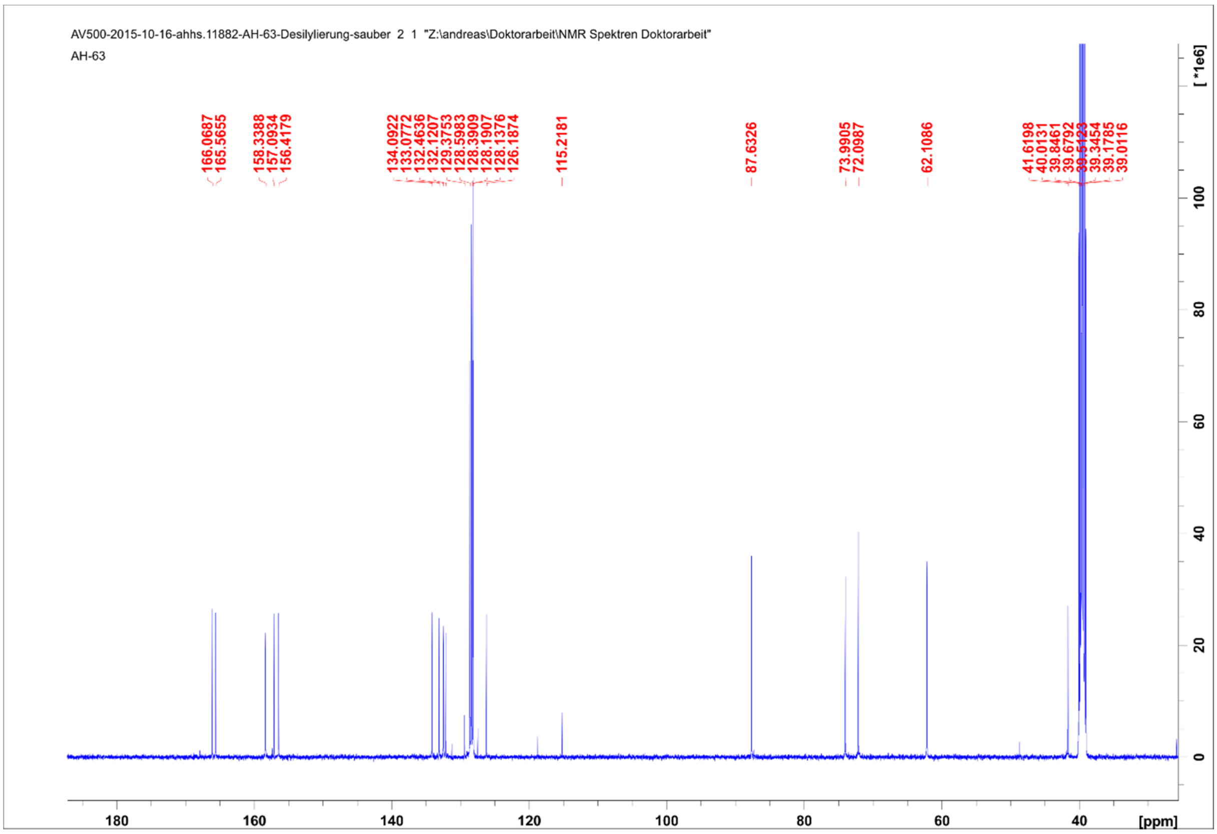

Figure S28: MALDI-spectrum

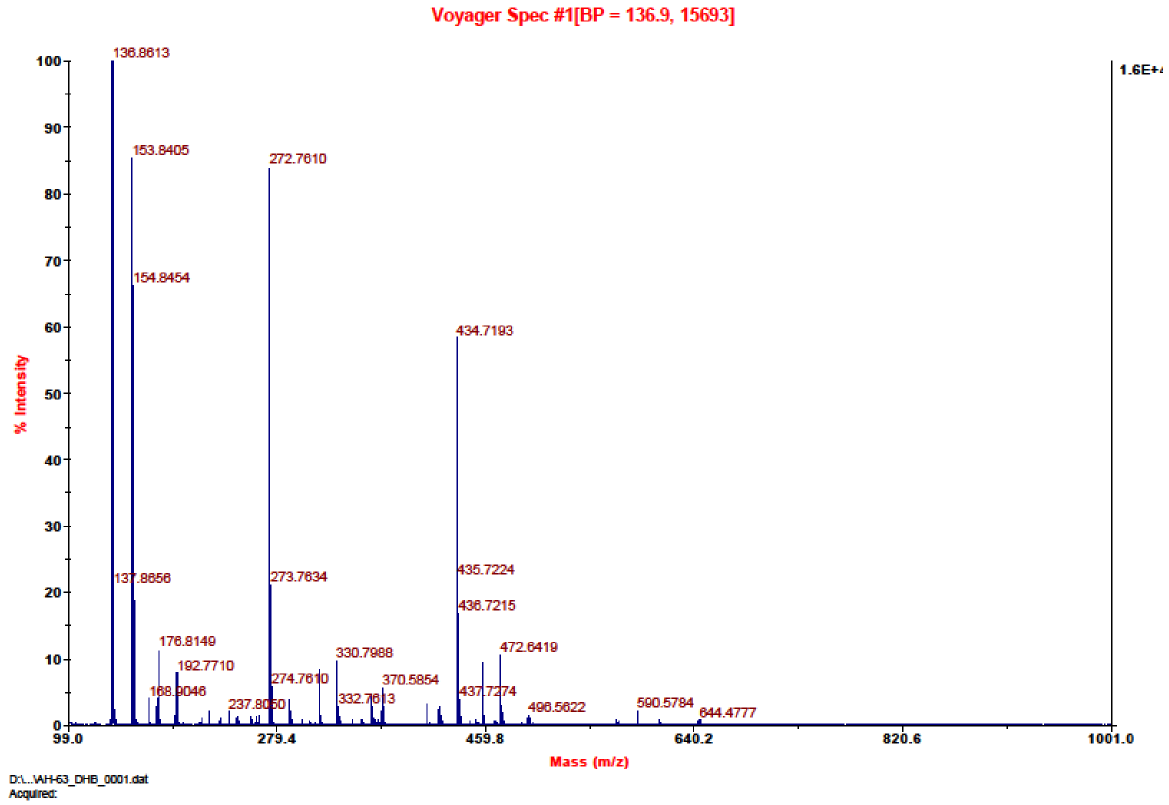

Analytics of 1β-[2,4-bis(benzoylamino)pyrimidin-5-yl]-1,2-dideoxy-5-*O*-(4,4'-dimethoxytriphenylmethyl)-D-ribofuranose (11)

Figure S29: ^{1}H-spectrum

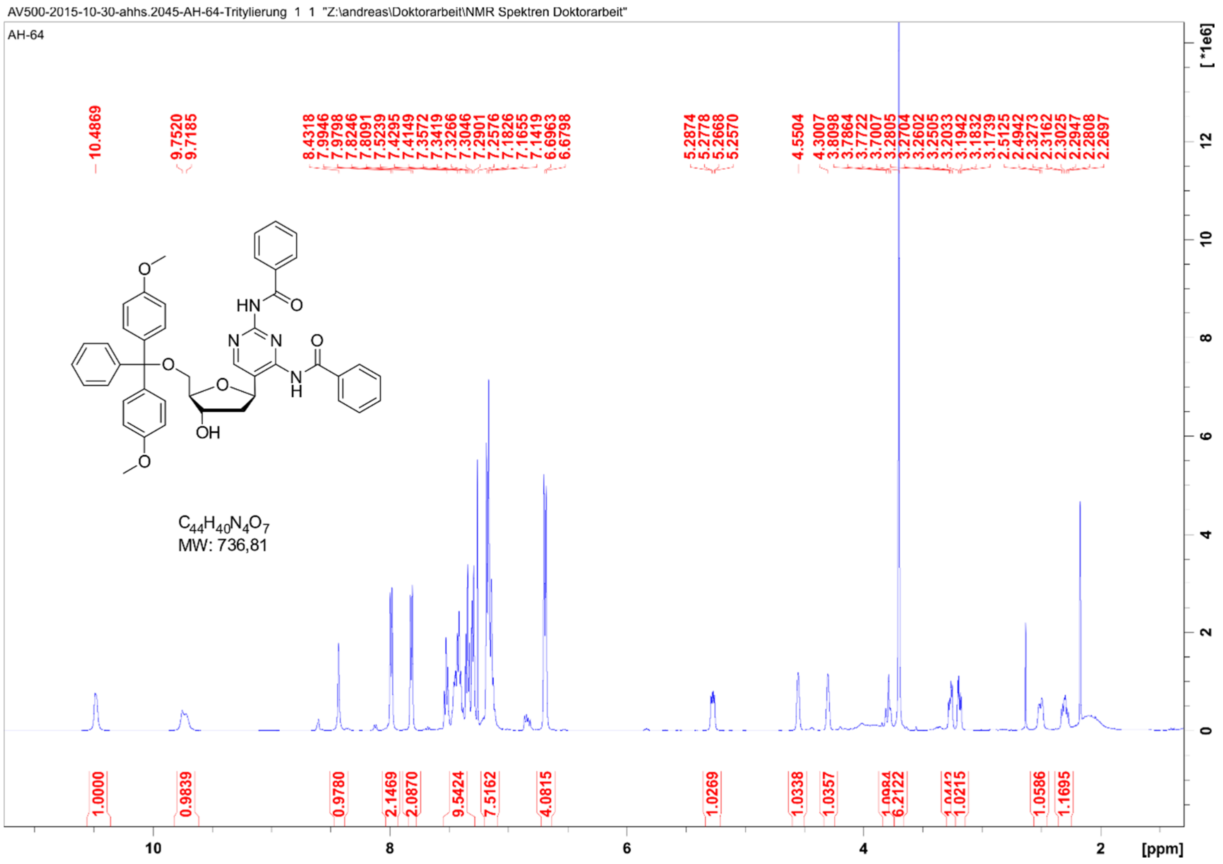

Figure S30: ^{13}C-spectrum

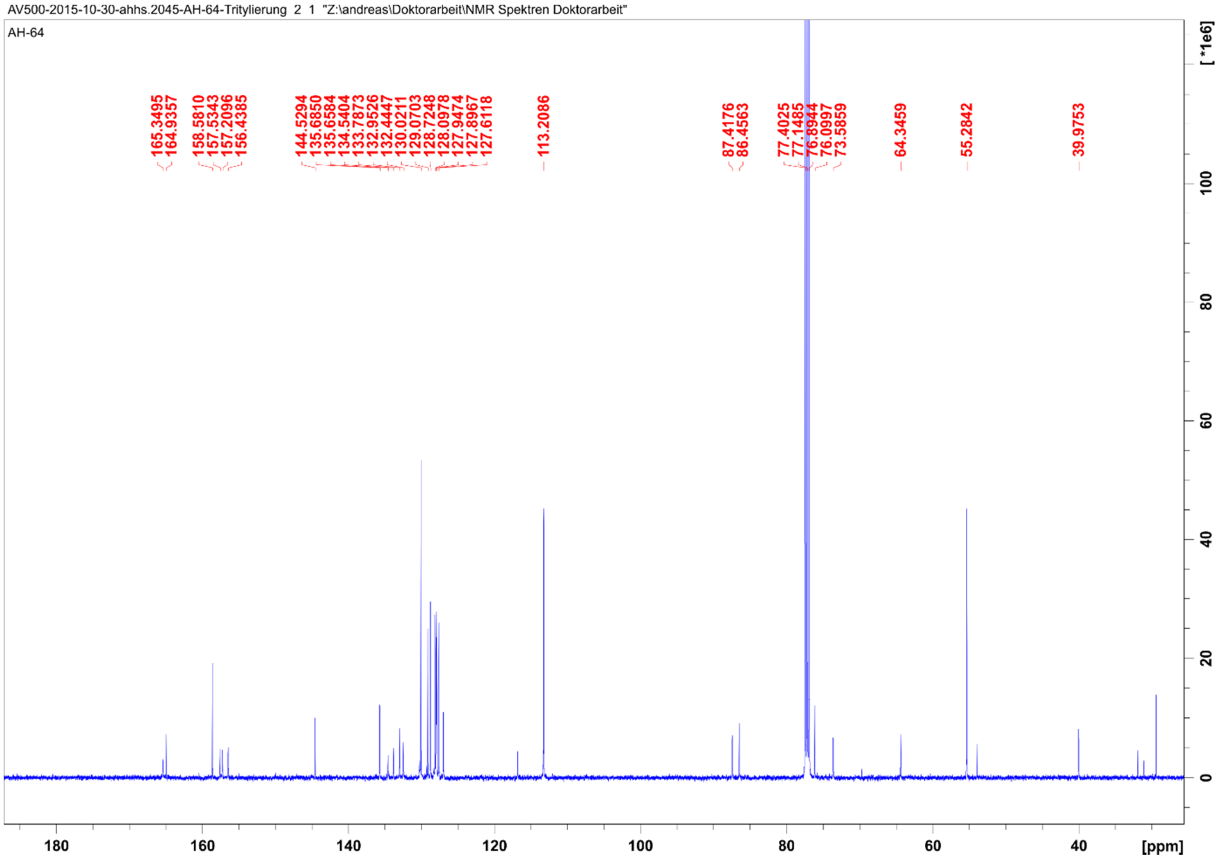

Figure S31: MALDI-spectrum

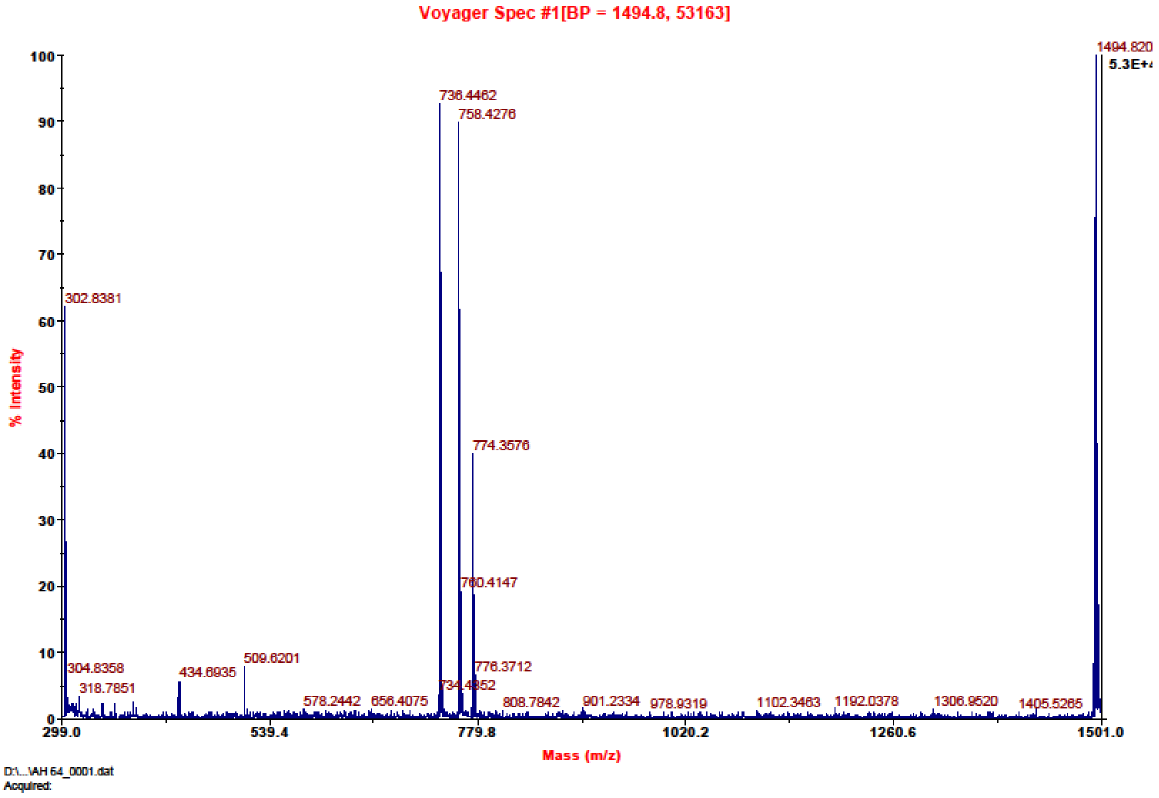

Analytics of 1*β*-[2,4-bis(benzoylamino)pyrimidin-5-yl]-1,2-dideoxy-5-*O*-(4,4'-dimethoxytriphenylmethyl)-D-ribofuranose-3-[(2-cyanoethyl)(*N,N*-diisopropyl)]phosphoramidite (12)

Figure S32: ^{1}H-spectrum

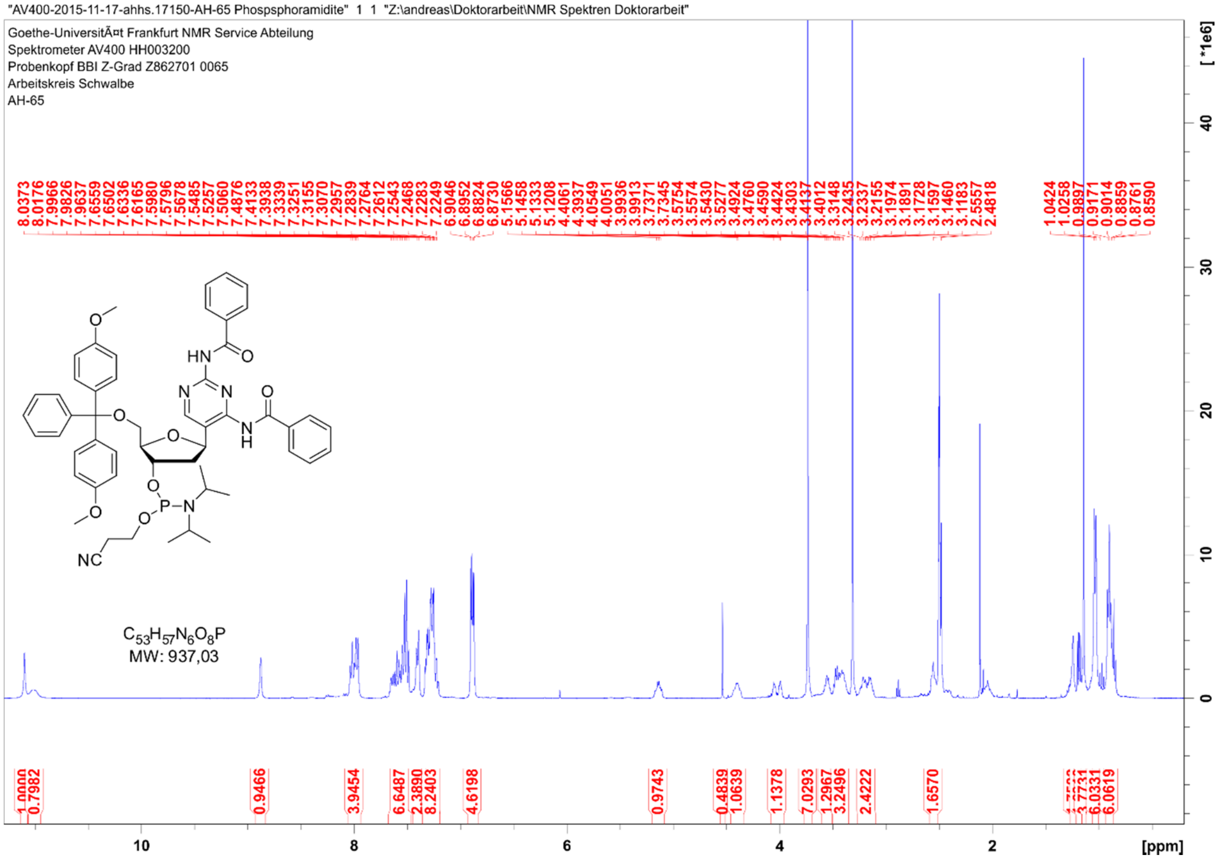

Figure S33: ^{31}P-spectrum

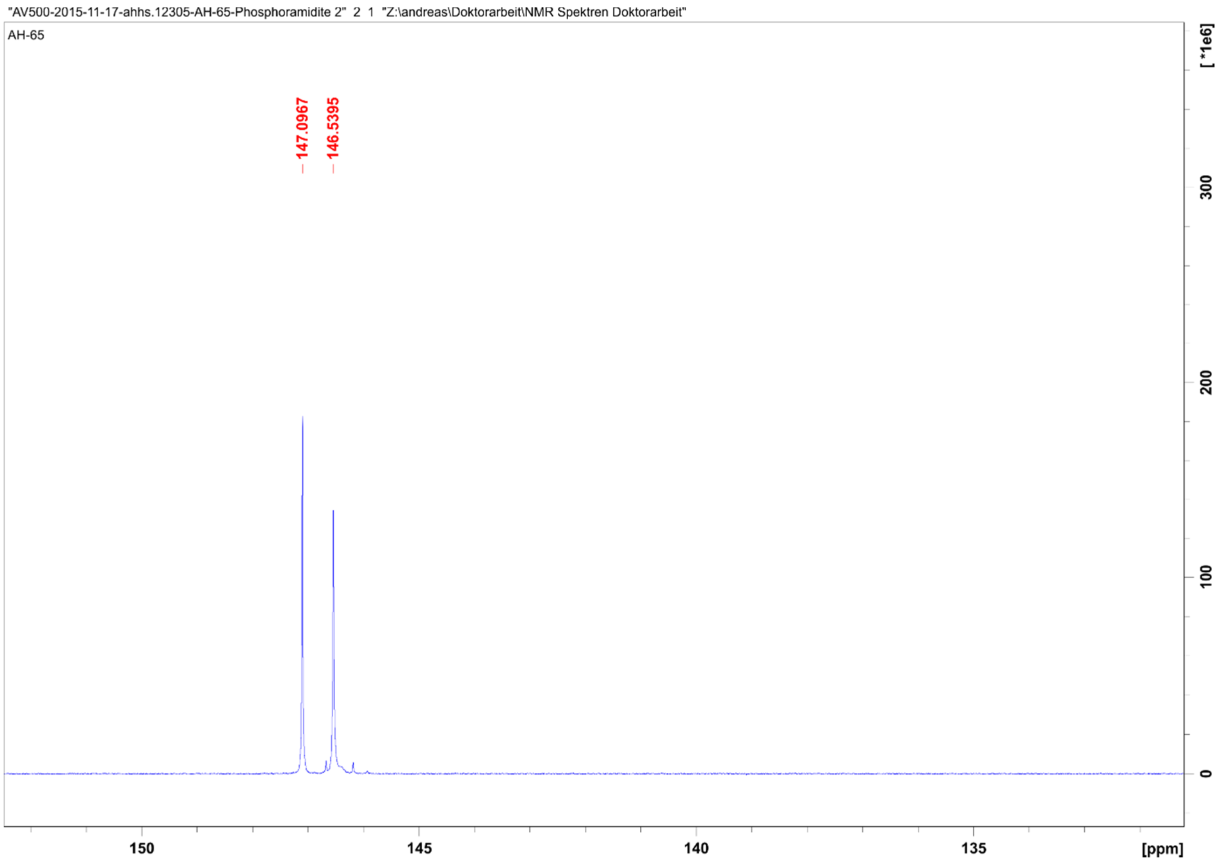

Figure S34: MALDI-spectrum

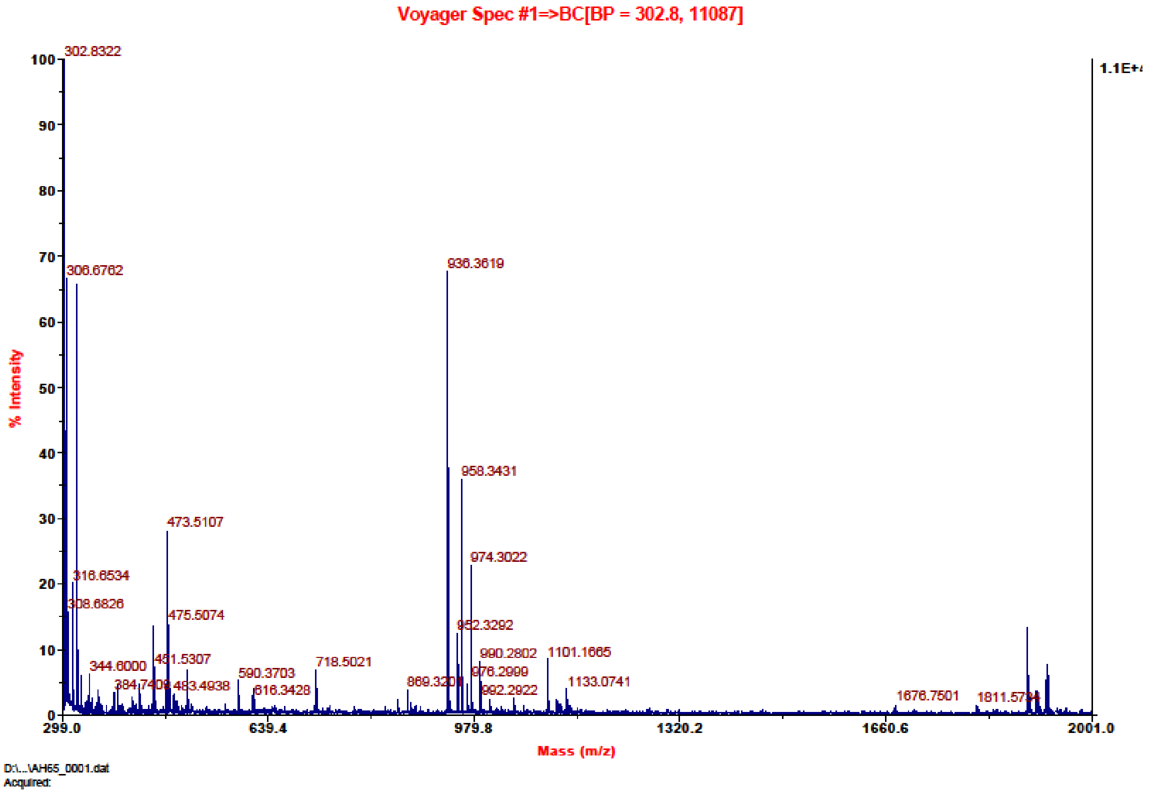

Analytics of *N*-(4-chloro-7*H*-pyrrolo[2,3-d]pyrimidin-2-yl)-2,2-dimethyl-propionamide (14)

Figure S35: ^{1}H-spectrum

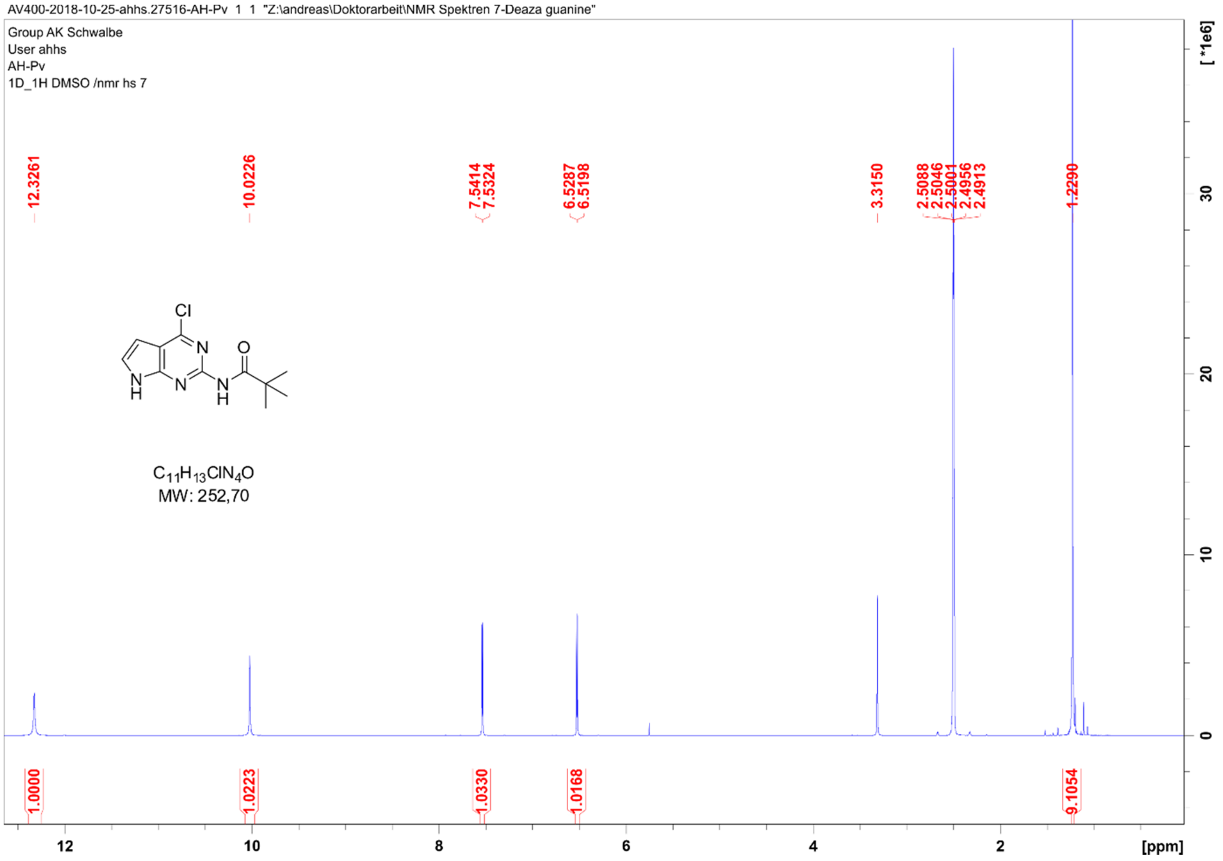

Figure S36: ^{13}C-spectrum

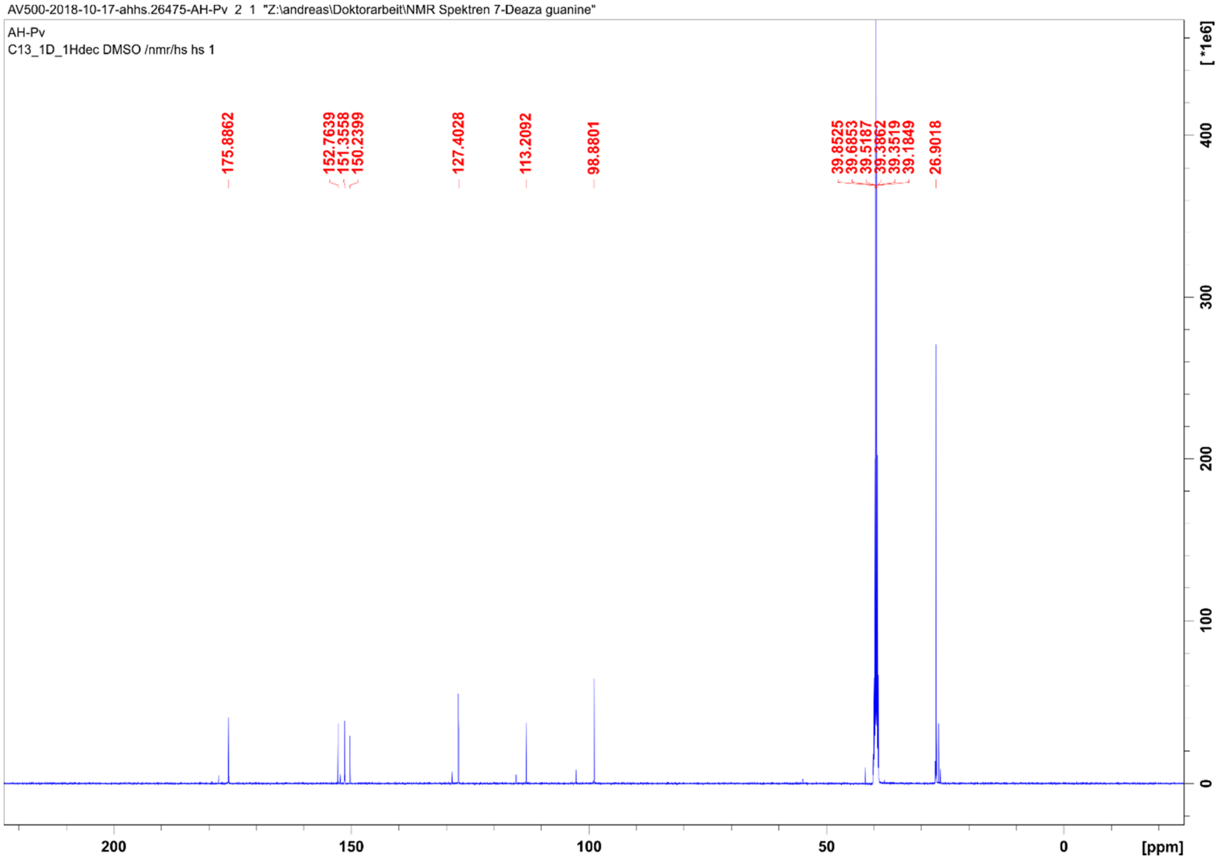

Figure S37: ESI-spectrum

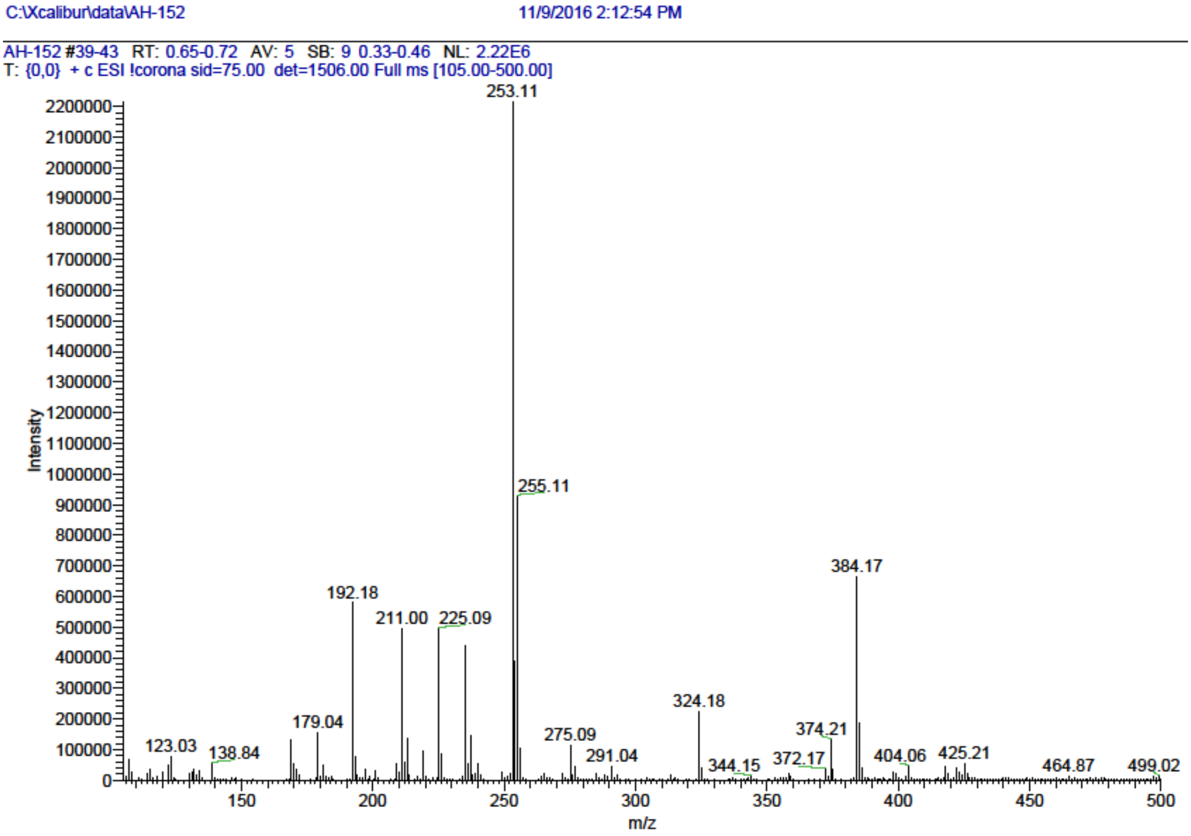

Analytics of *N*-(4-chloro-5-iodo-7*H*-pyrrolo[2,3-d]pyrimidin-2-yl)-2,2-dimethyl-propionamide (15)

Figure S38: ^{1}H-spectrum

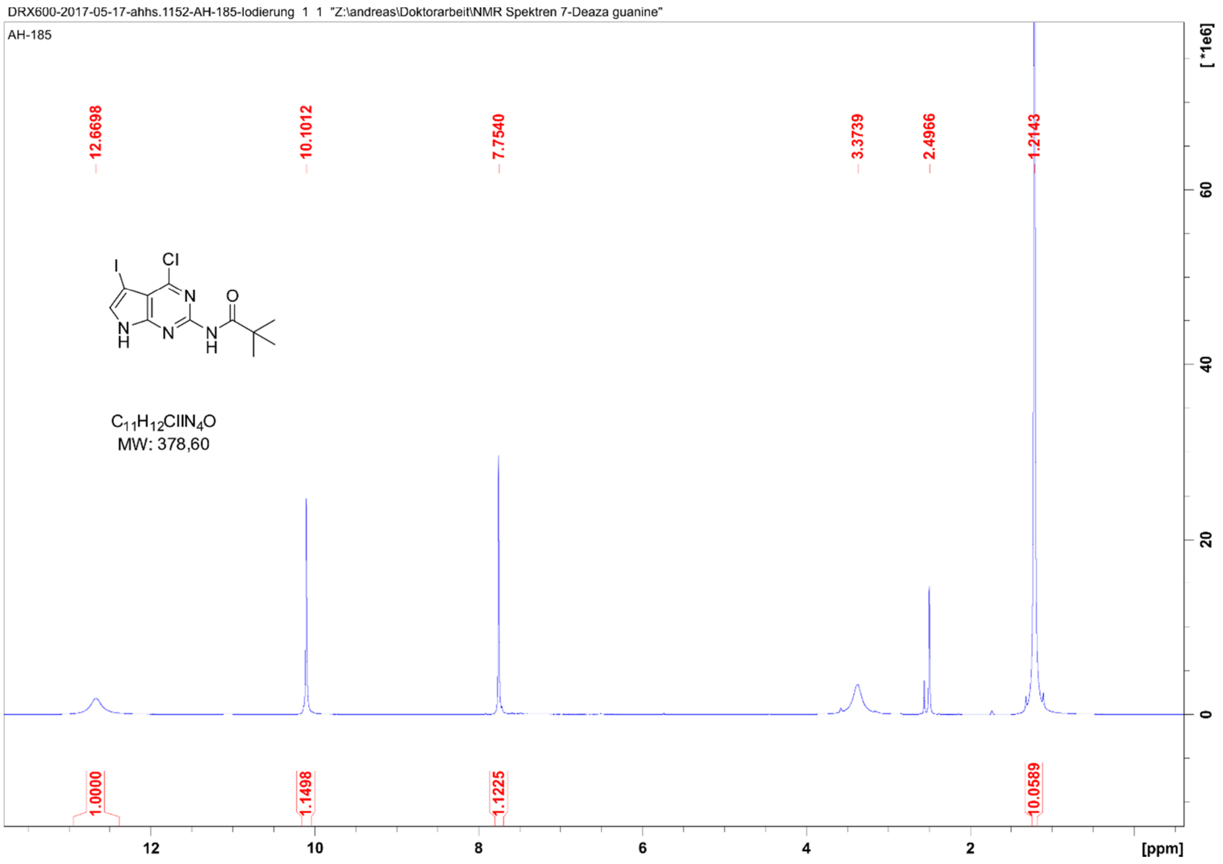

Figure S39: ^{13}C-spectrum

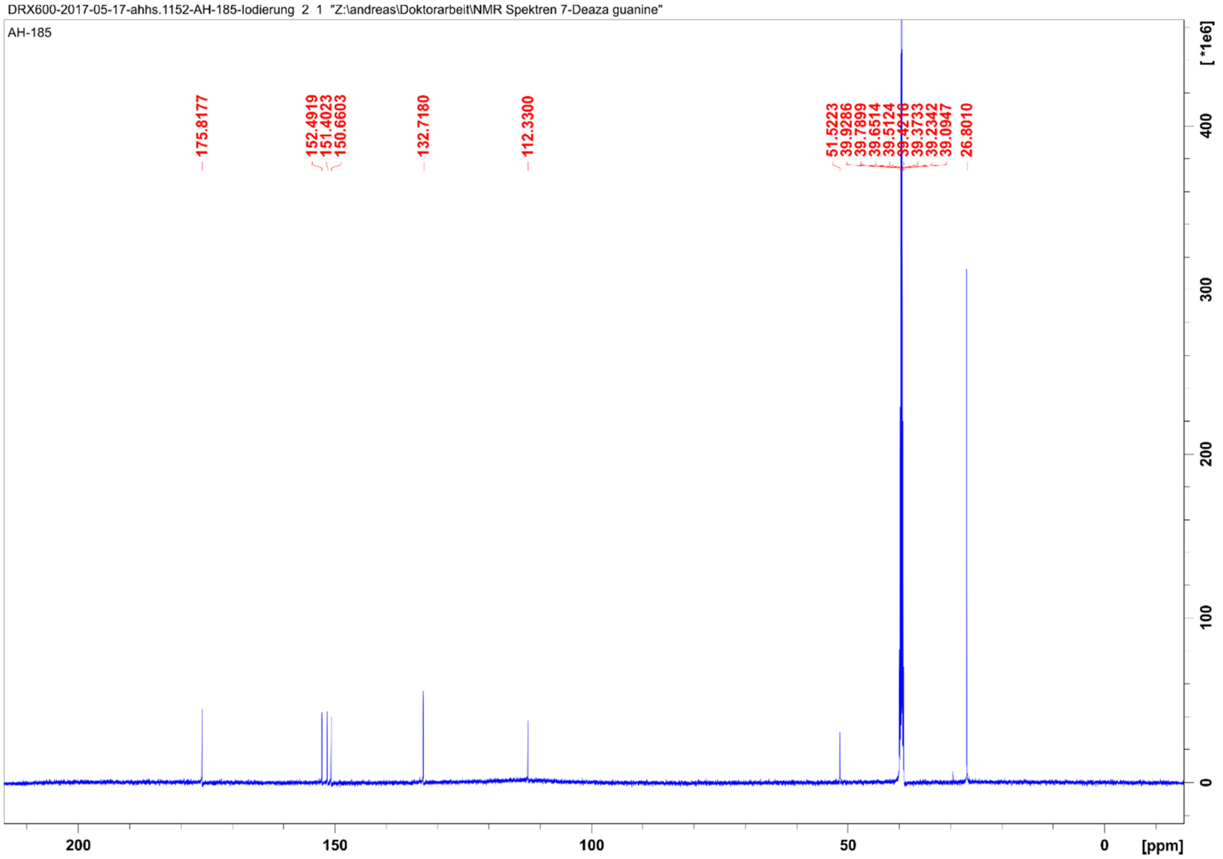

Figure S40: ESI-spectrum

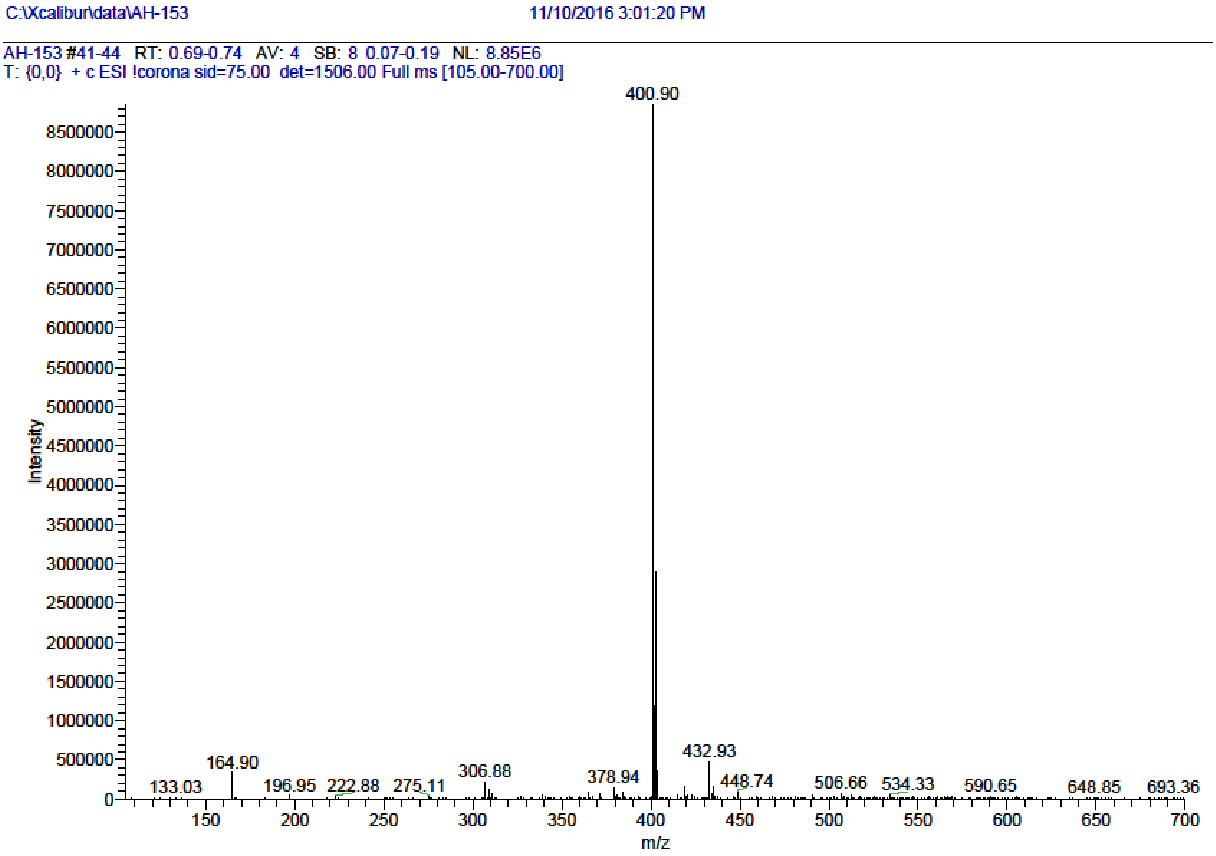

Analytics of 4-chloro-5-iodo-2-pivaloylamino-7-[(2,3,5-tri-*O*-benzoyl)-*β*-D-ribofuranosyl]-7*H*-pyrrolo[2,3-d]pyrimidine (16)

Figure S41: ^{1}H-spectrum

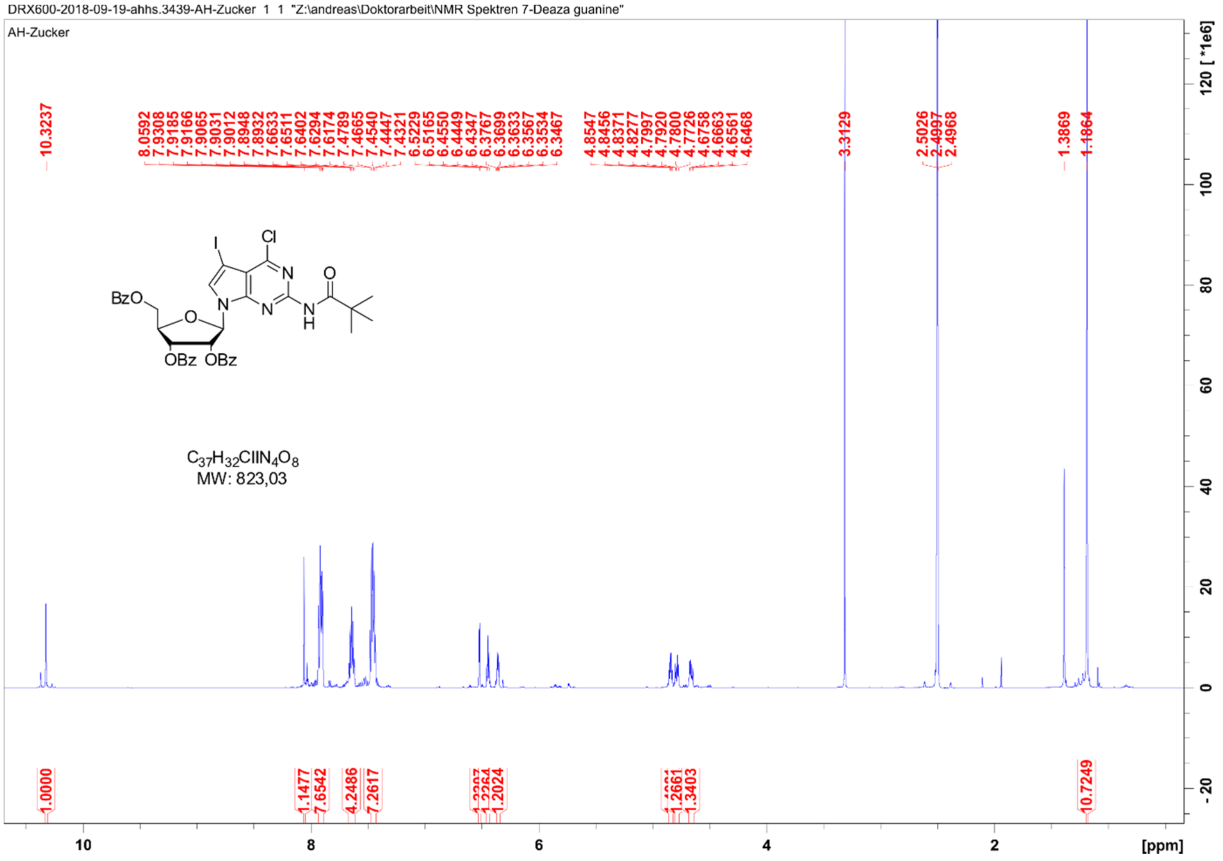

Figure S42: ^{13}C-spectrum

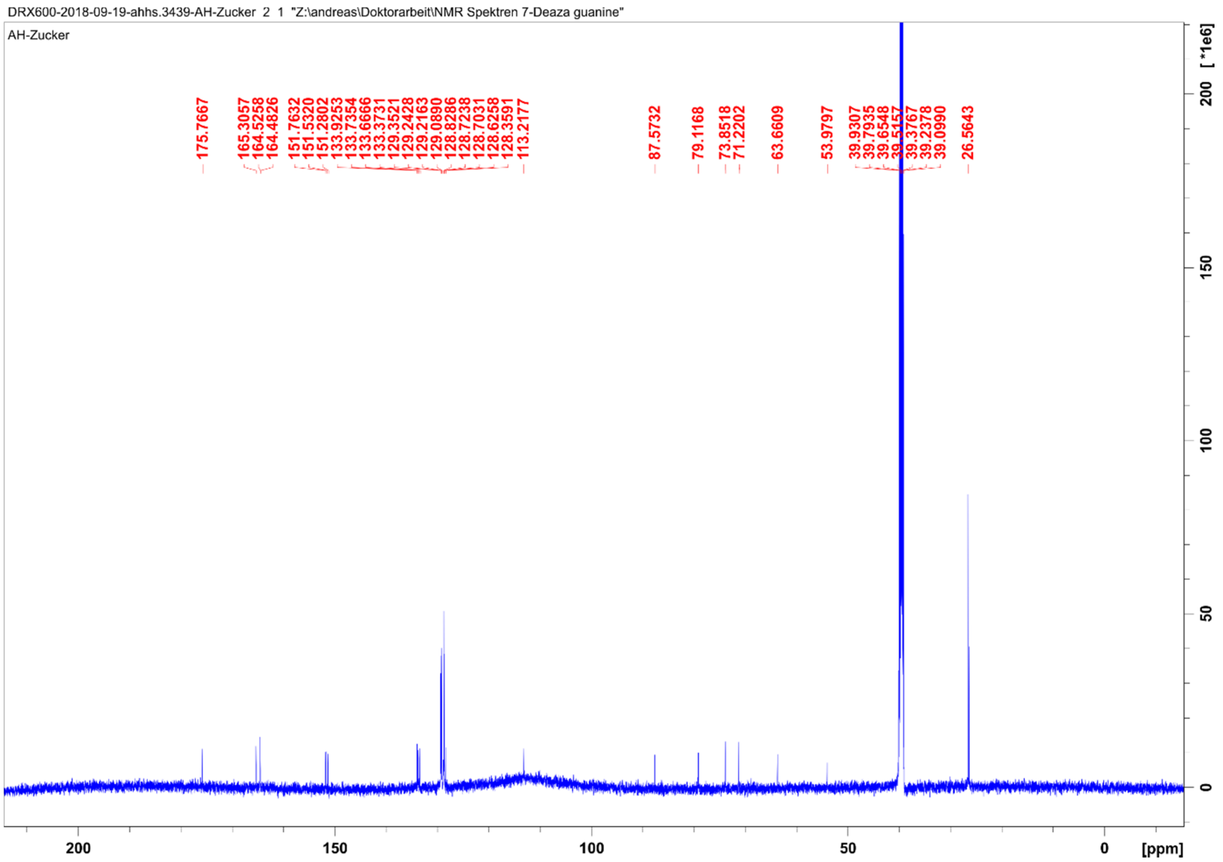

Figure S43: ESI-spectrum

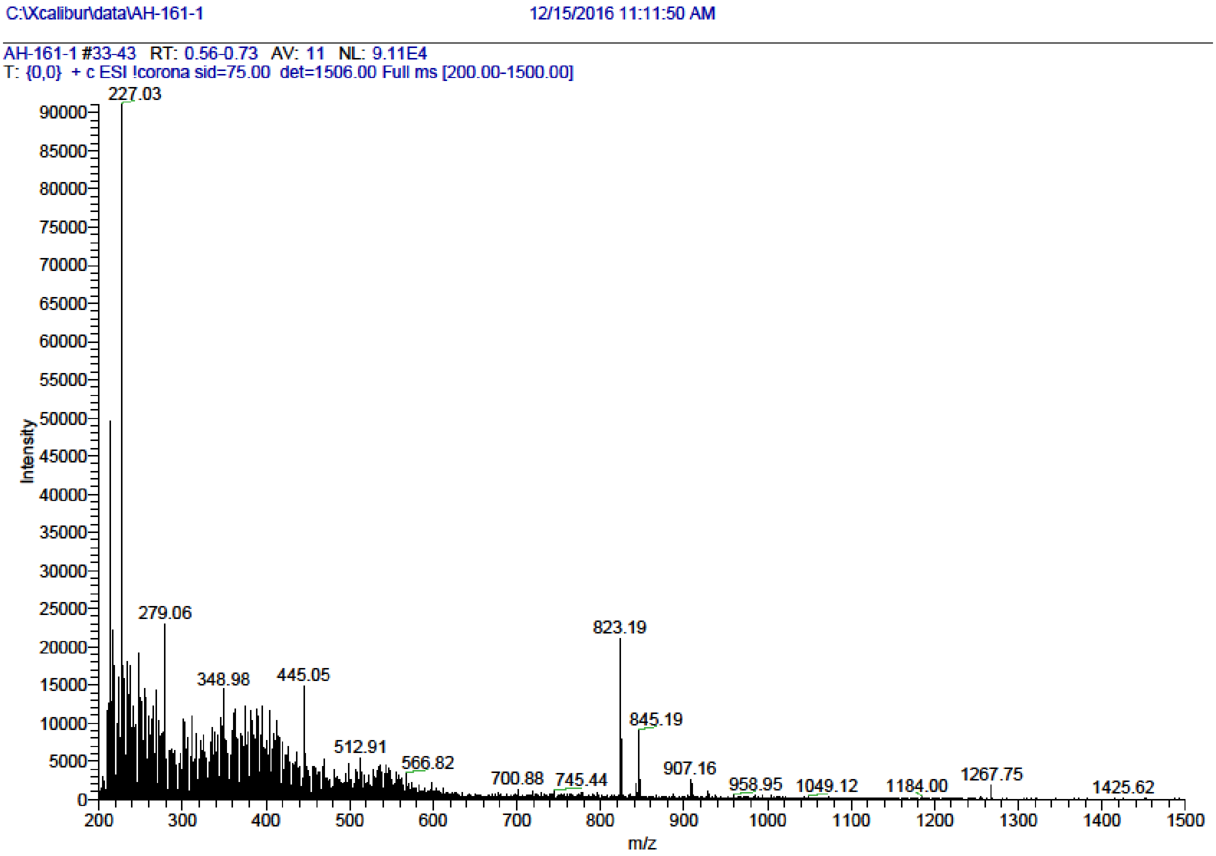

Analytics of 2-amino-5-iodo-3,7-dihydro-7-(*β*-D-ribofuranosyl)-4*H*-pyrrolo-[2,3-d]pyrimidin-4-one (17)

Figure S44: ^{1}H-spectrum

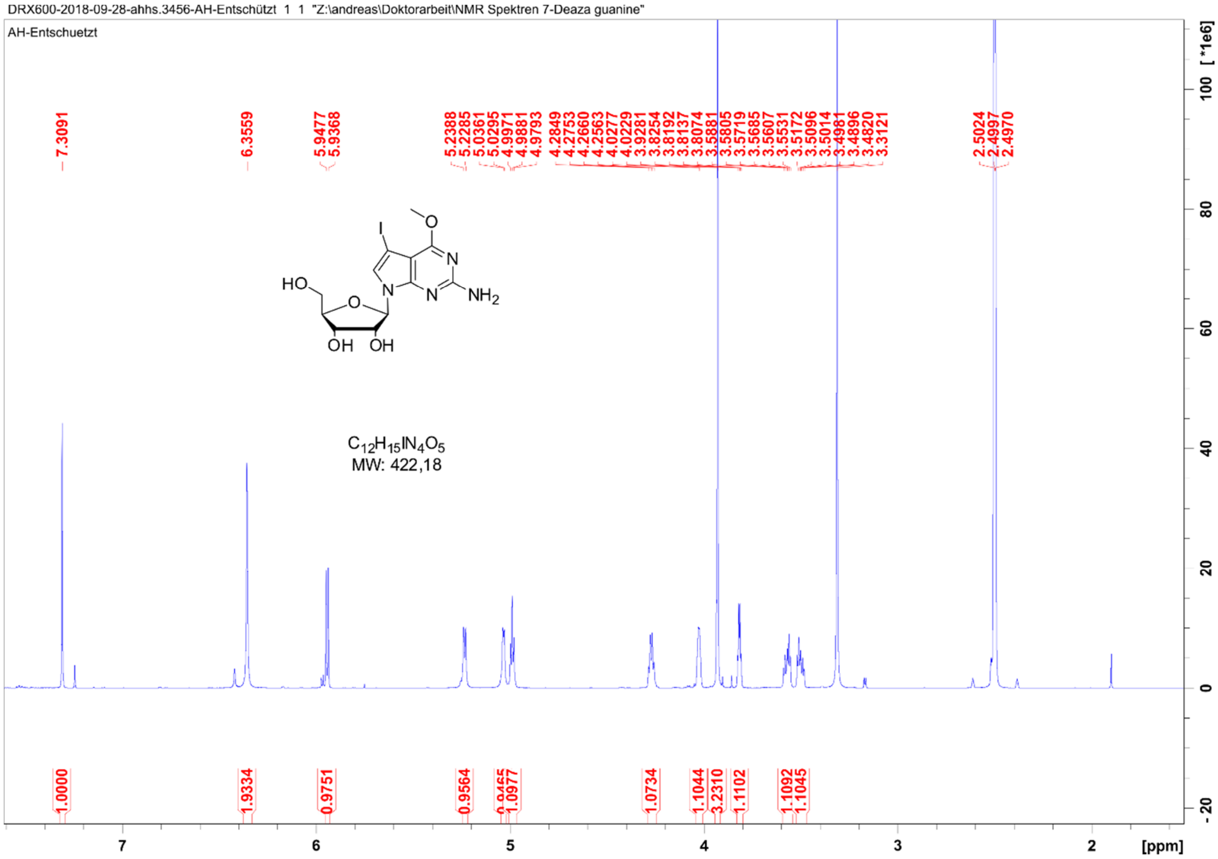

Figure S45: ^{13}C-spectrum

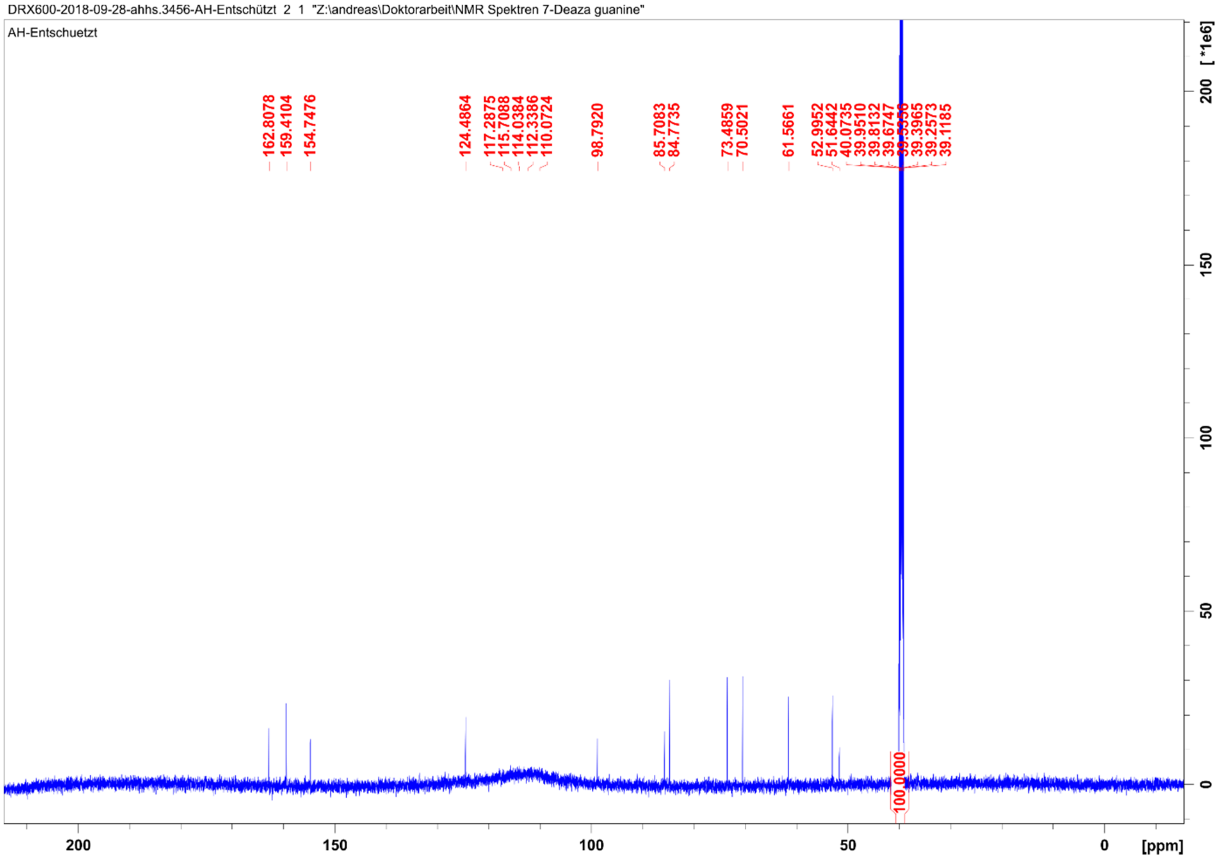

Figure S46: ESI-spectrum

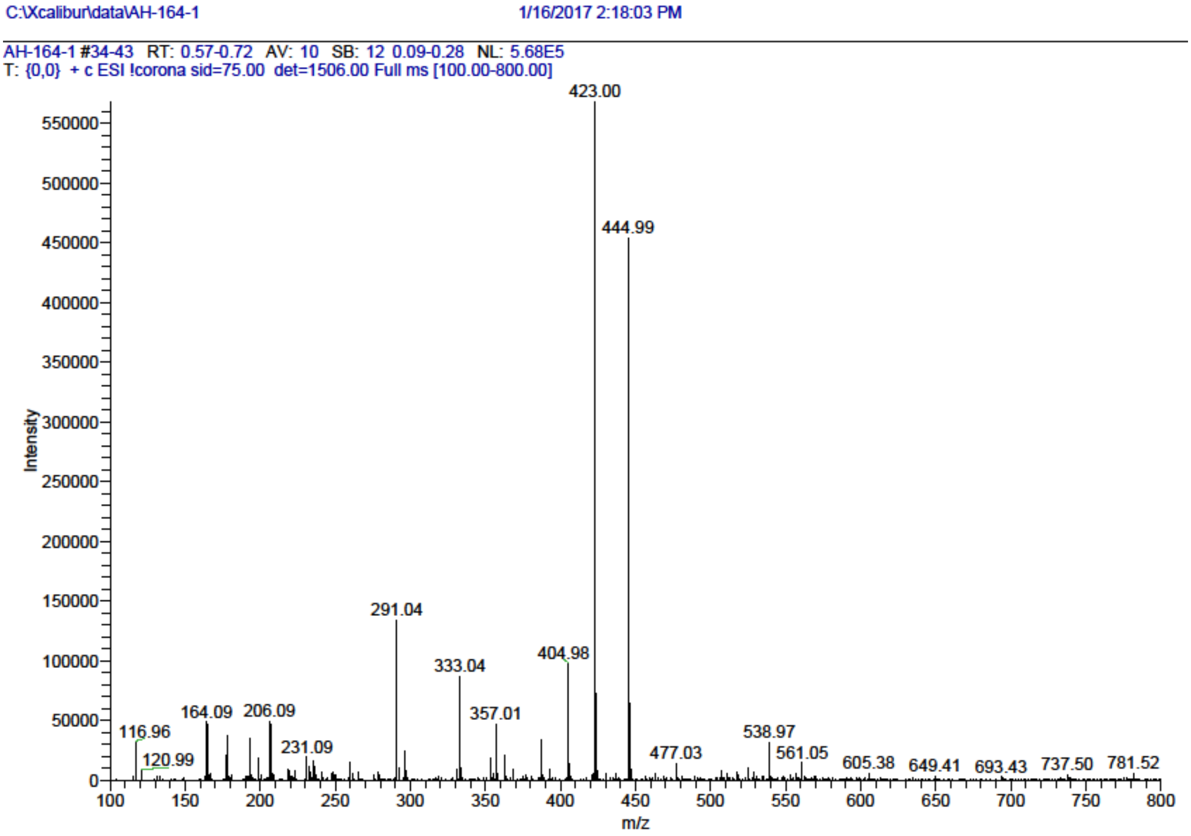

Analytics of 1,7-dihydro-5-iodo-4-methoxy-7-(*β*-D-ribofuranosyl)-2*H*-pyrrolo[2,3-d]pyrimidin-2-amine (18)

Figure S47: ^{1}H-spectrum

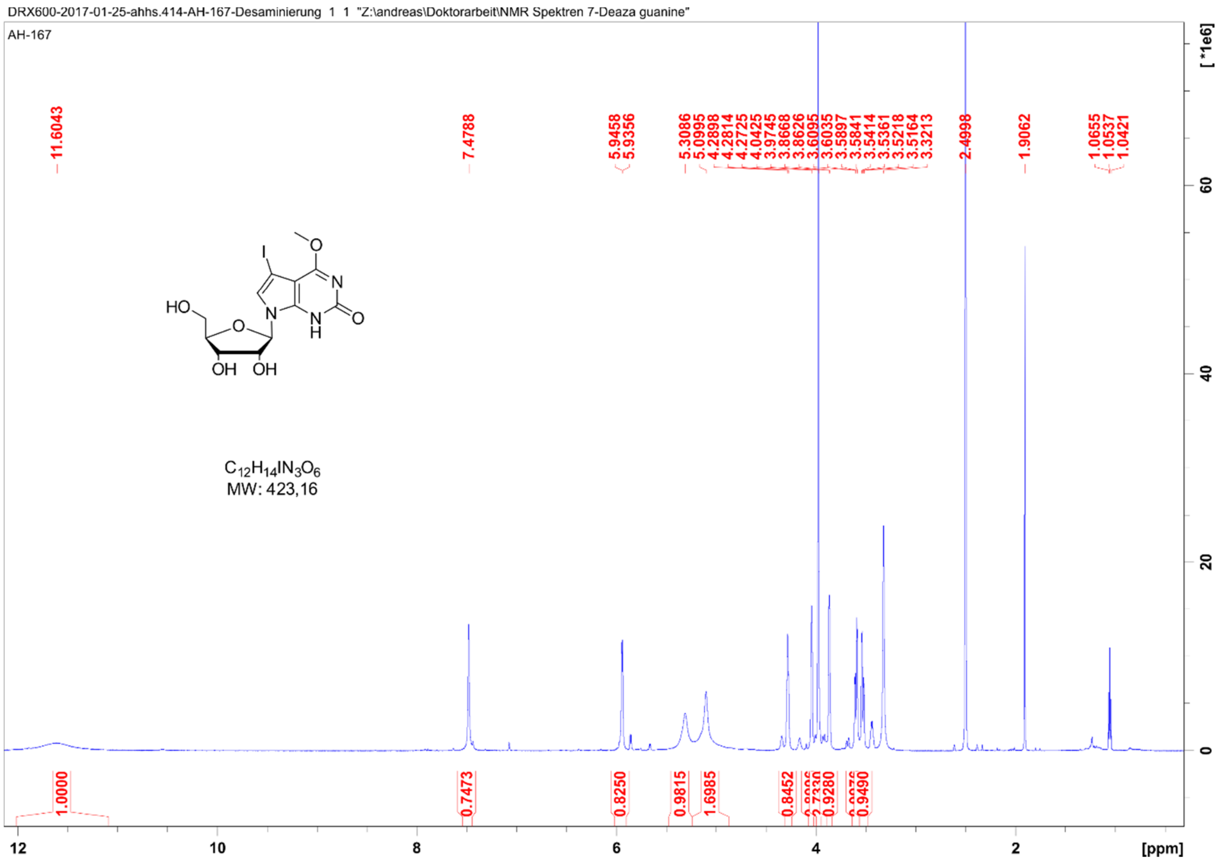

Figure S48: ^{13}C-spectrum

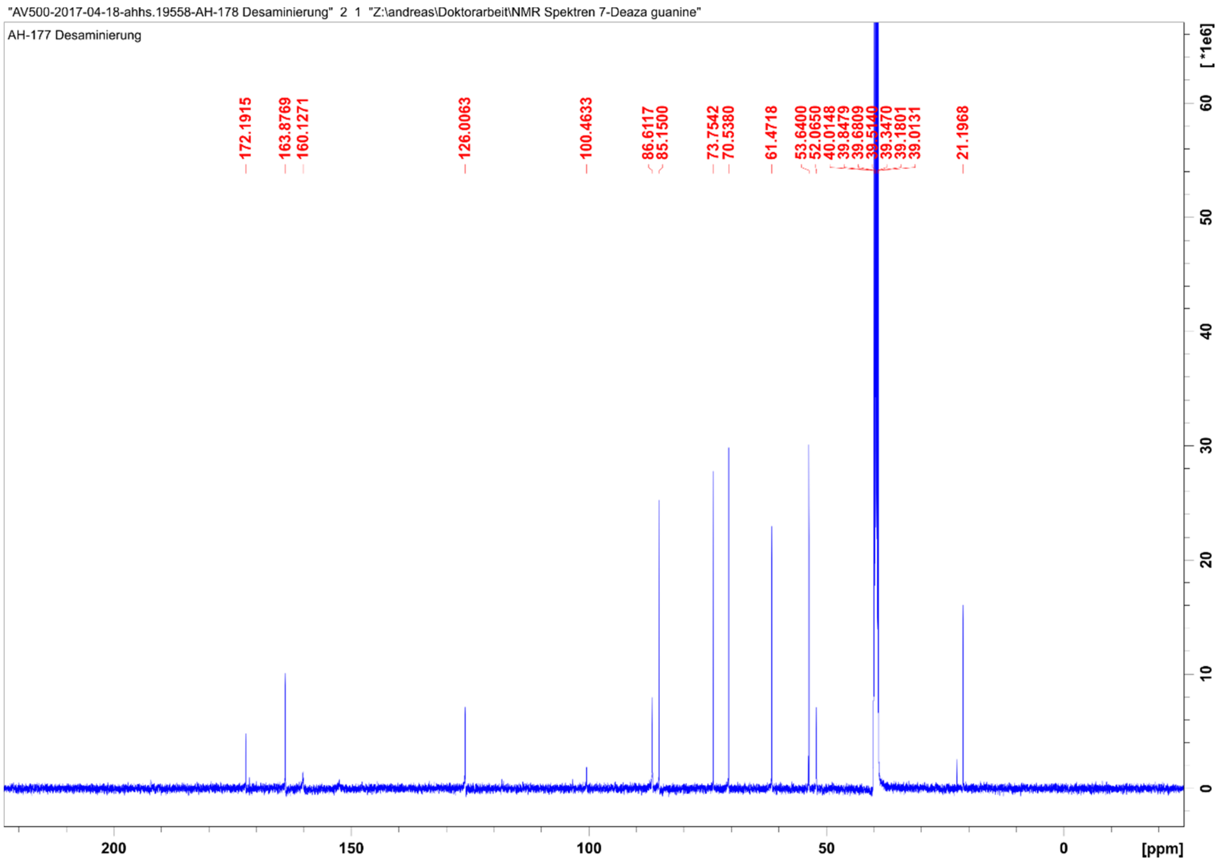

Figure S49: ESI-spectrum

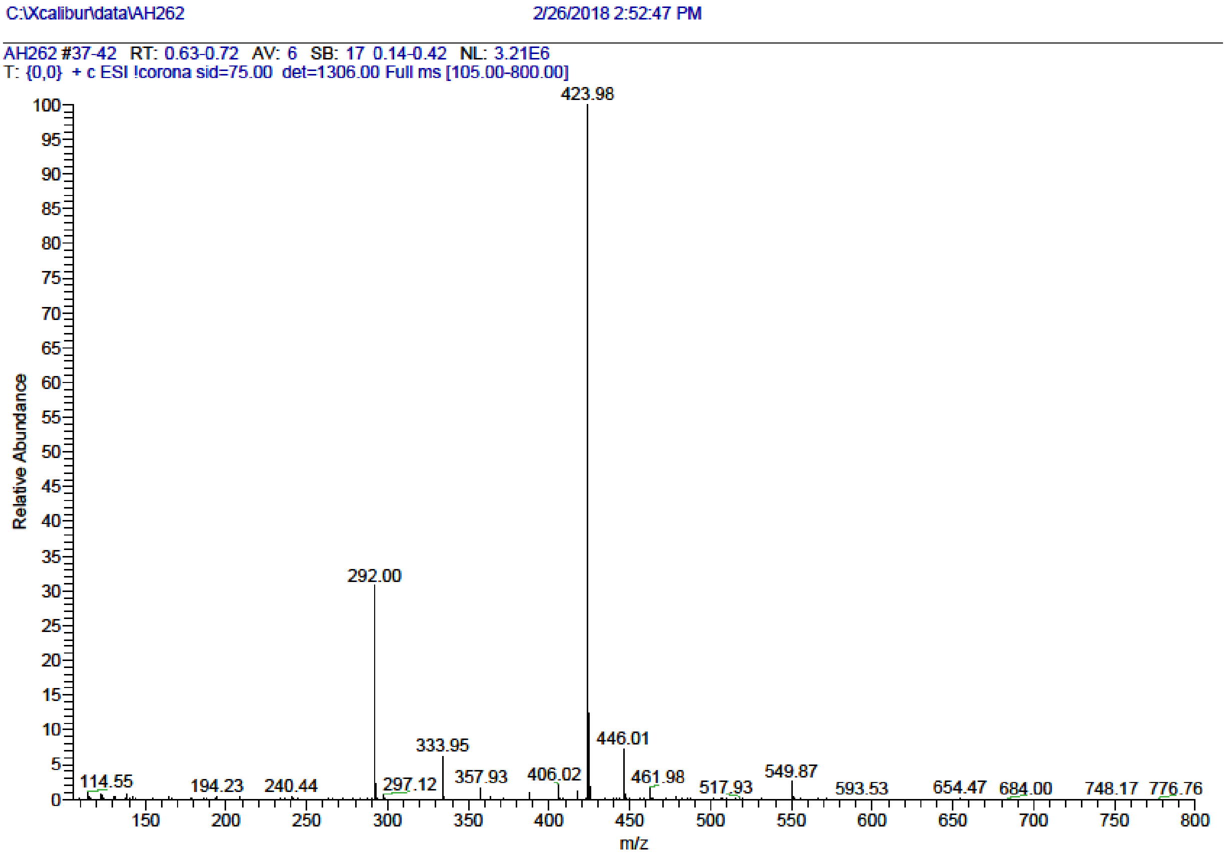

Analytics of 1,7-dihydro-5-(octa-1,7-diynyl)-4-methoxy-7-(*β*-D-ribofuranosyl)-2*H*-pyrrolo[2,3-d]pyrimidin-2-amine (19)

Figure S50: ^{1}H-spectrum

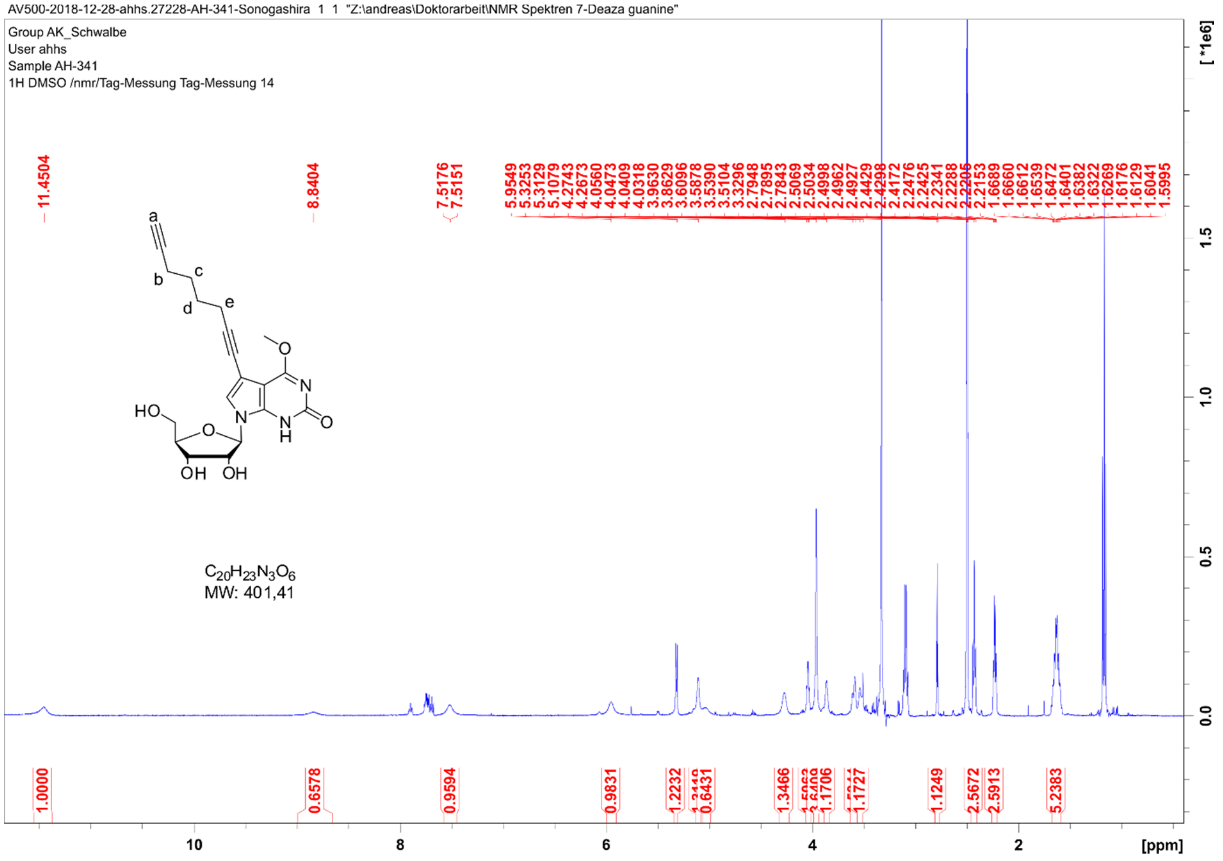

Figure S51: 13C-spectrum

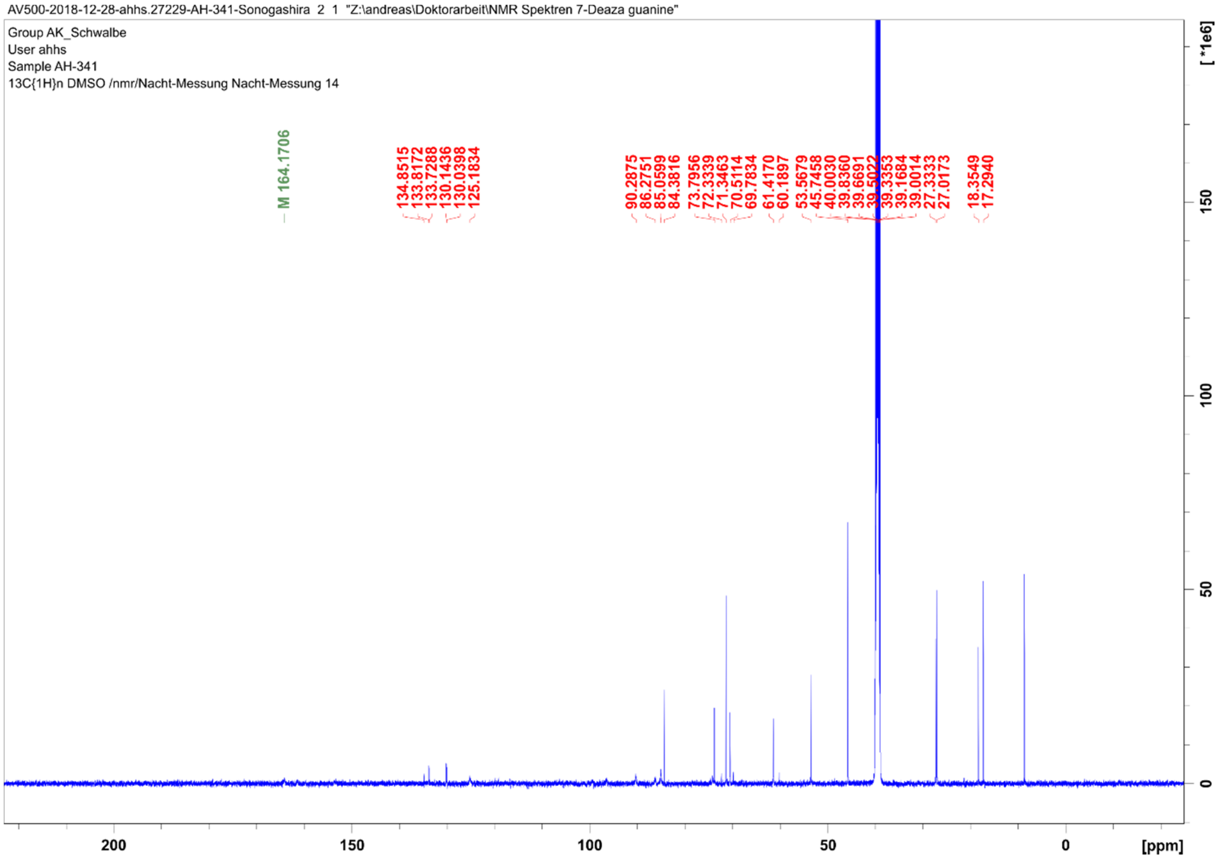

Figure S52: ESI-spectrum

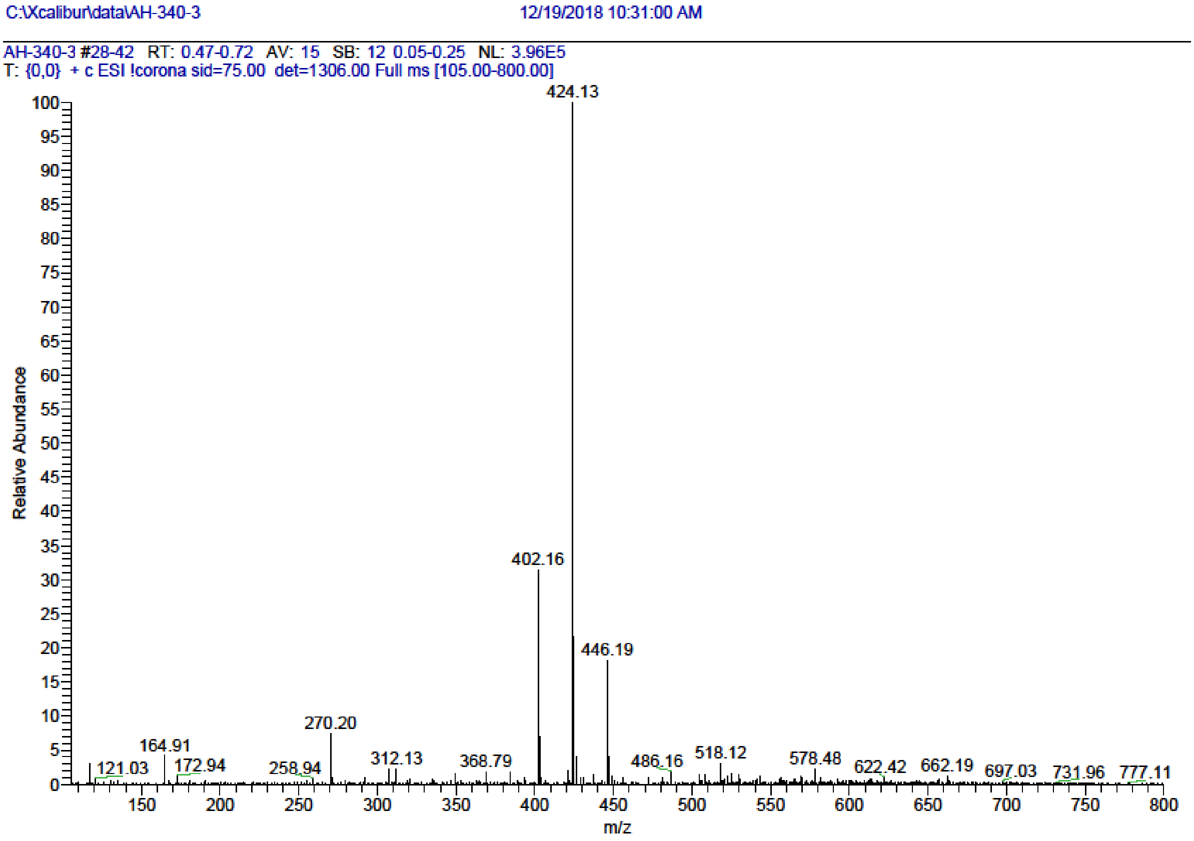

Analytics of 5-(octa-1,7-diynyl)-7-(*β*-D-ribofuranosyl)-1,3,7-trihydro-2*H*,4*H*-pyrrolo-[2,3-d]pyrimidin-2,4-dione (20)

Figure S53: ^{1}H-spectrum

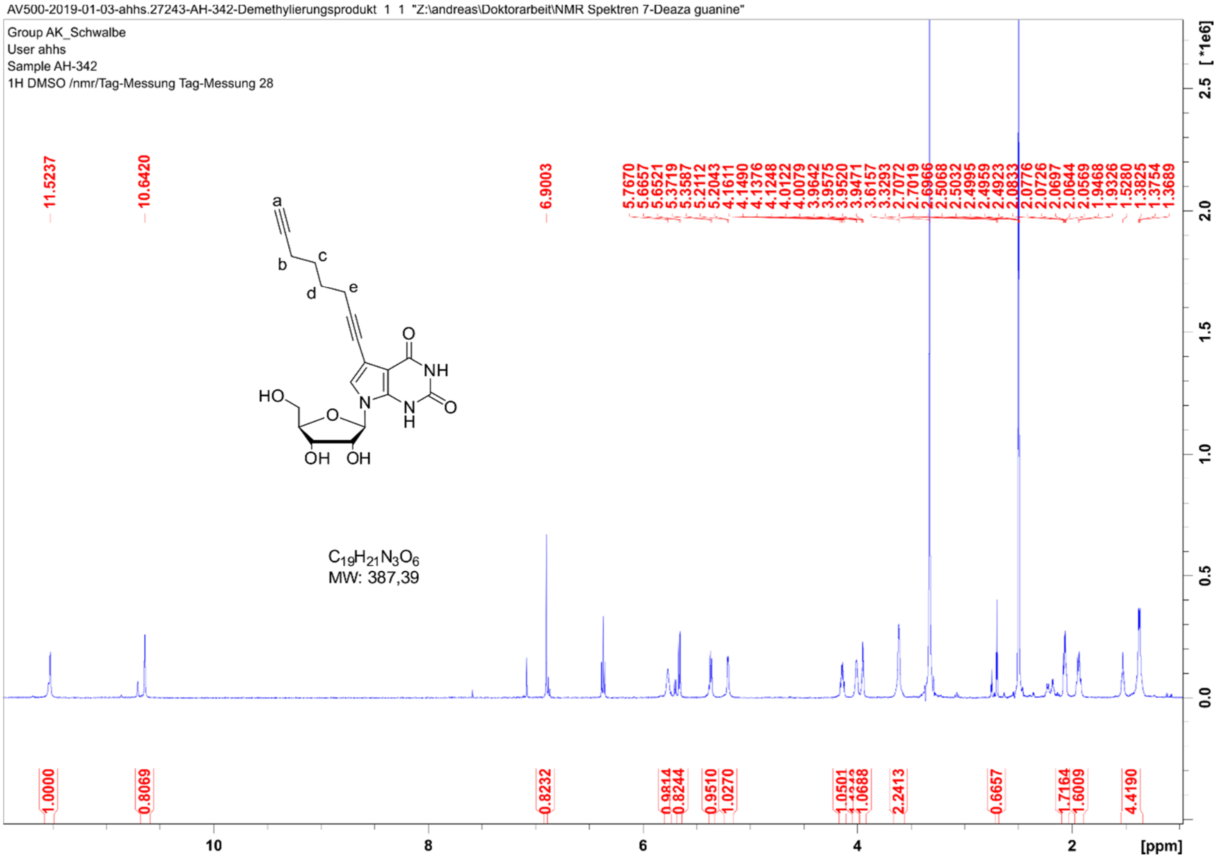

Figure S54: ^{13}C-spectrum

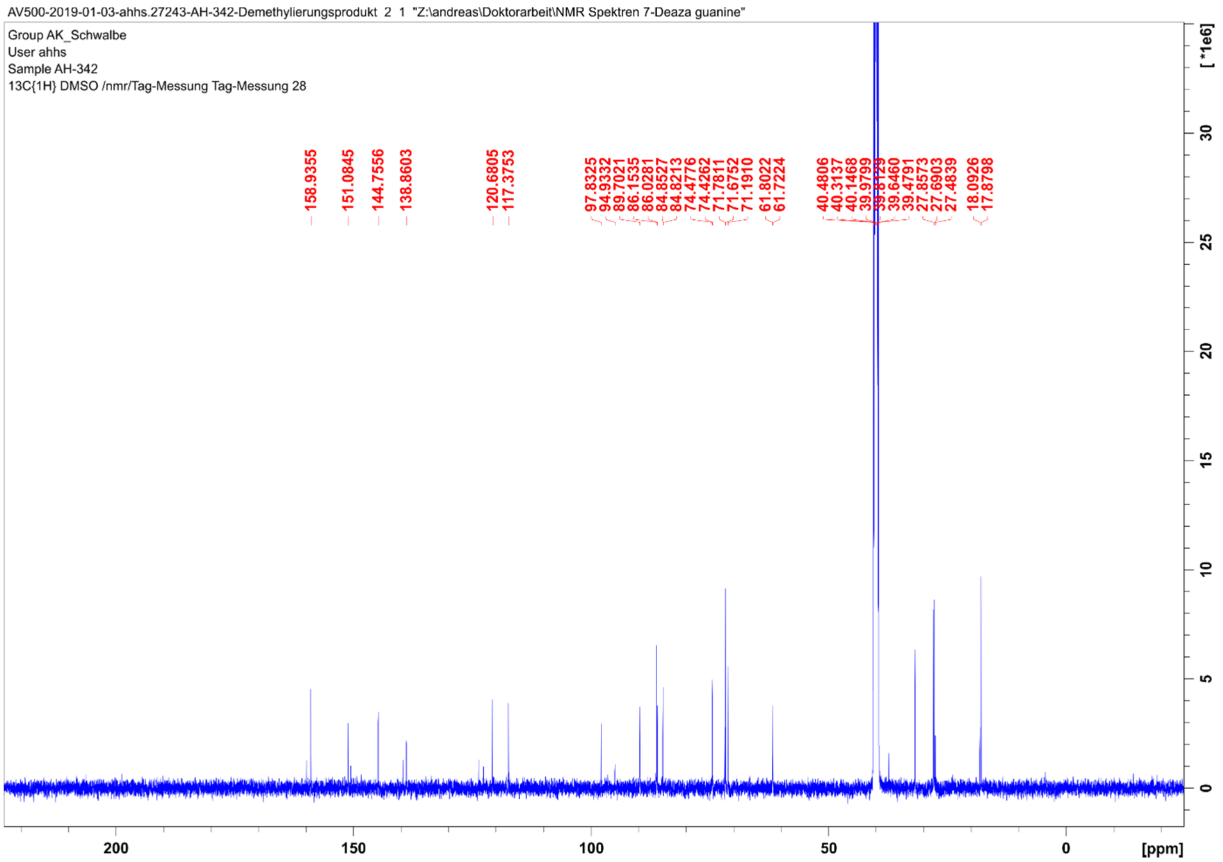

Figure S55: ESI-spectrum

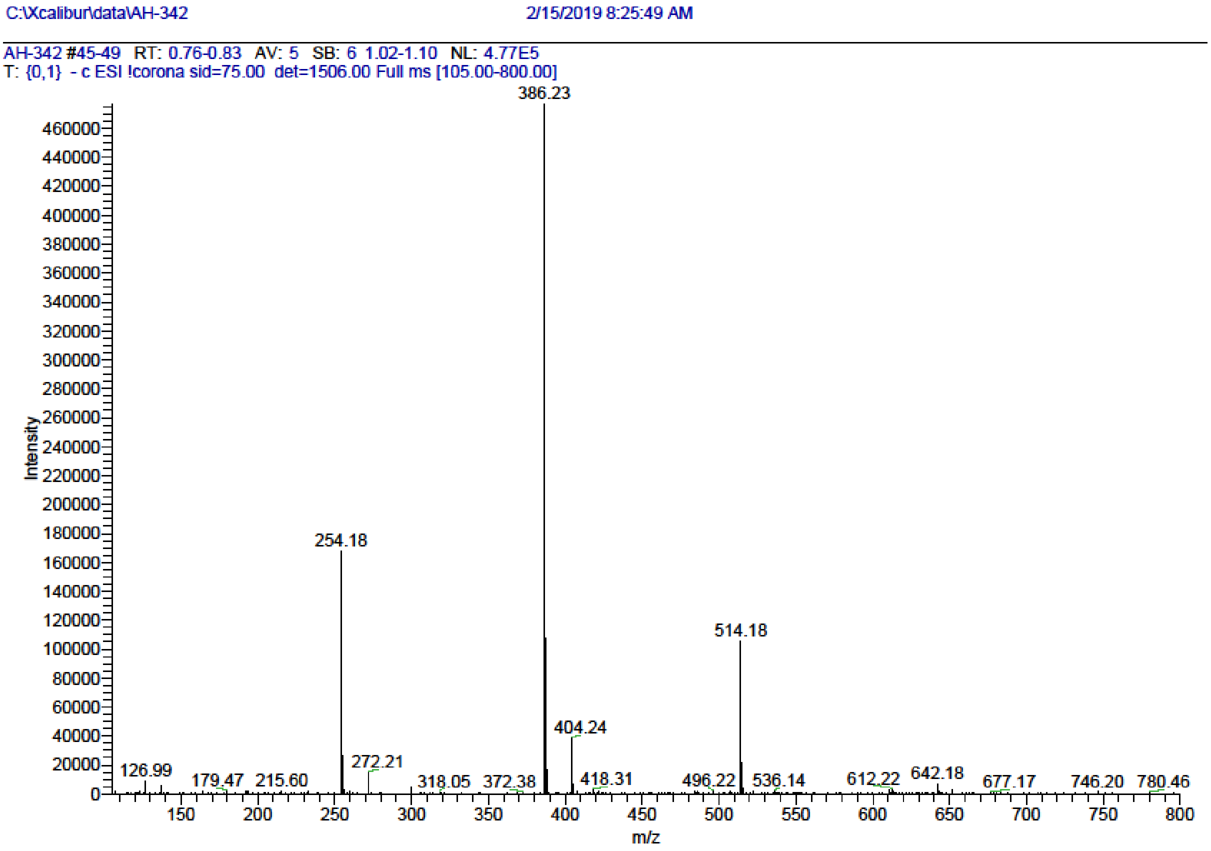

Analytics of 5-(octa-1,7-diynyl)-7-(*β*-D-ribofuranosyl)-1,3,7-trihydro-2*H*,4*H*-pyrrolo-[2,3-d]pyrimidin-2,4-dione TP (21)

Figure S56: ^{1}H-spectrum

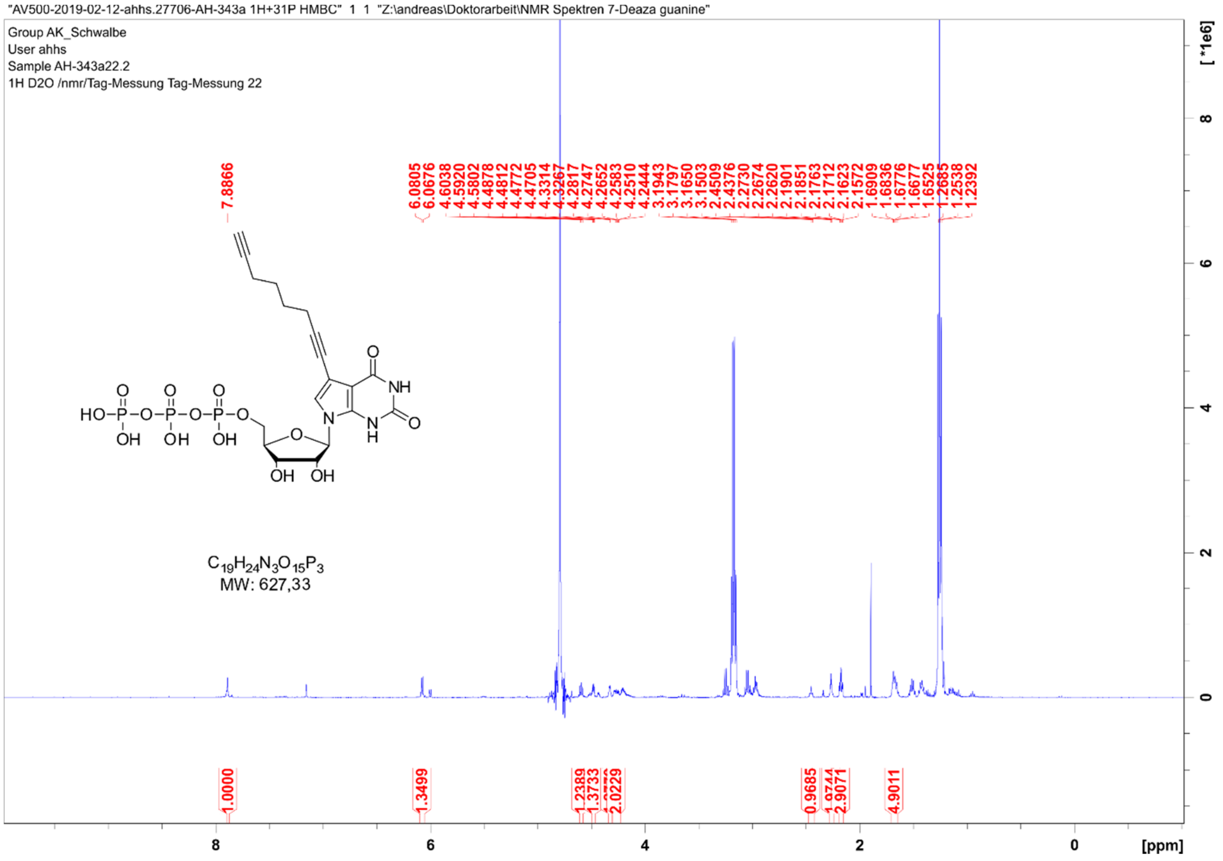

Figure S57: ^{31}P-spectrum

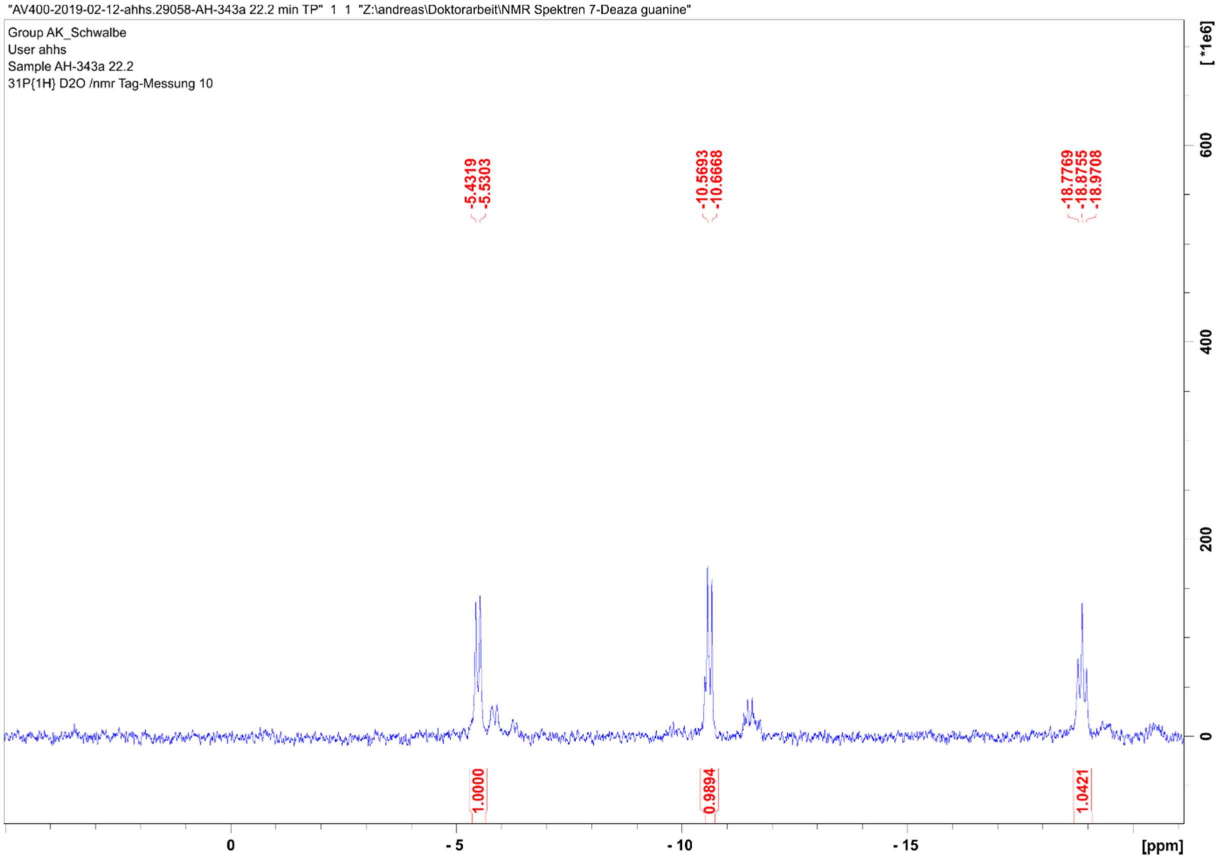

Figure S58: ^{31}P-HMBC-spectrum

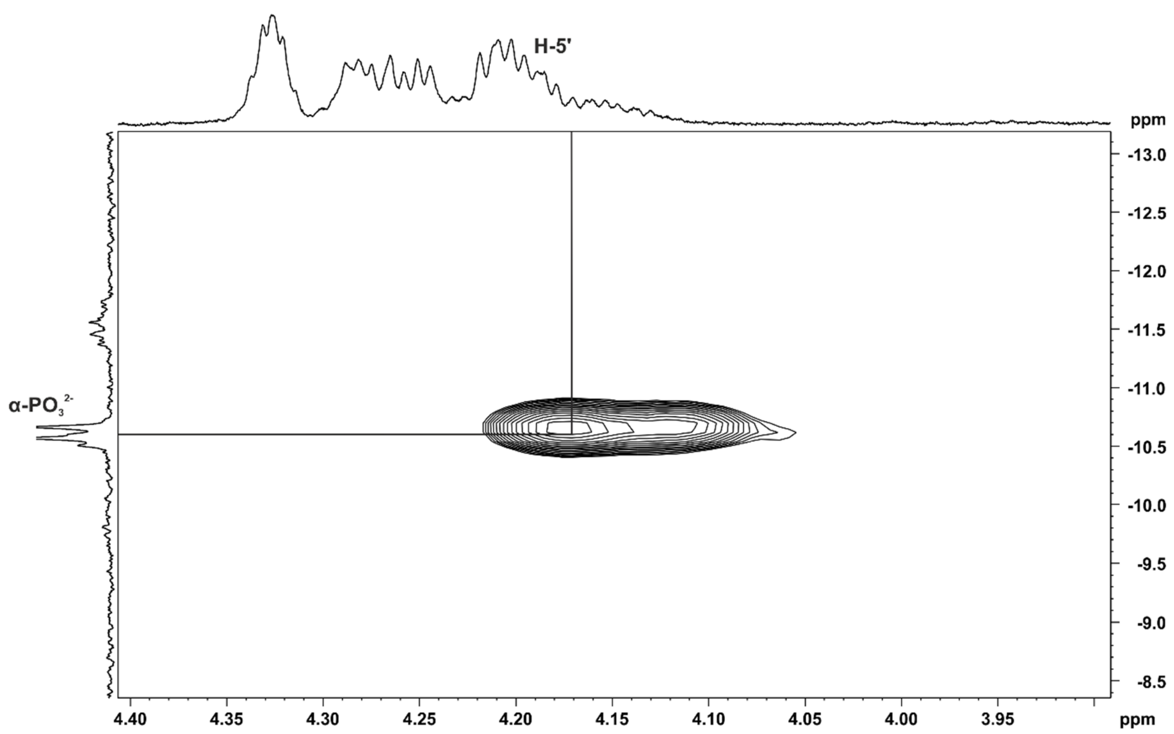

Figure S59: MALDI-spectrum

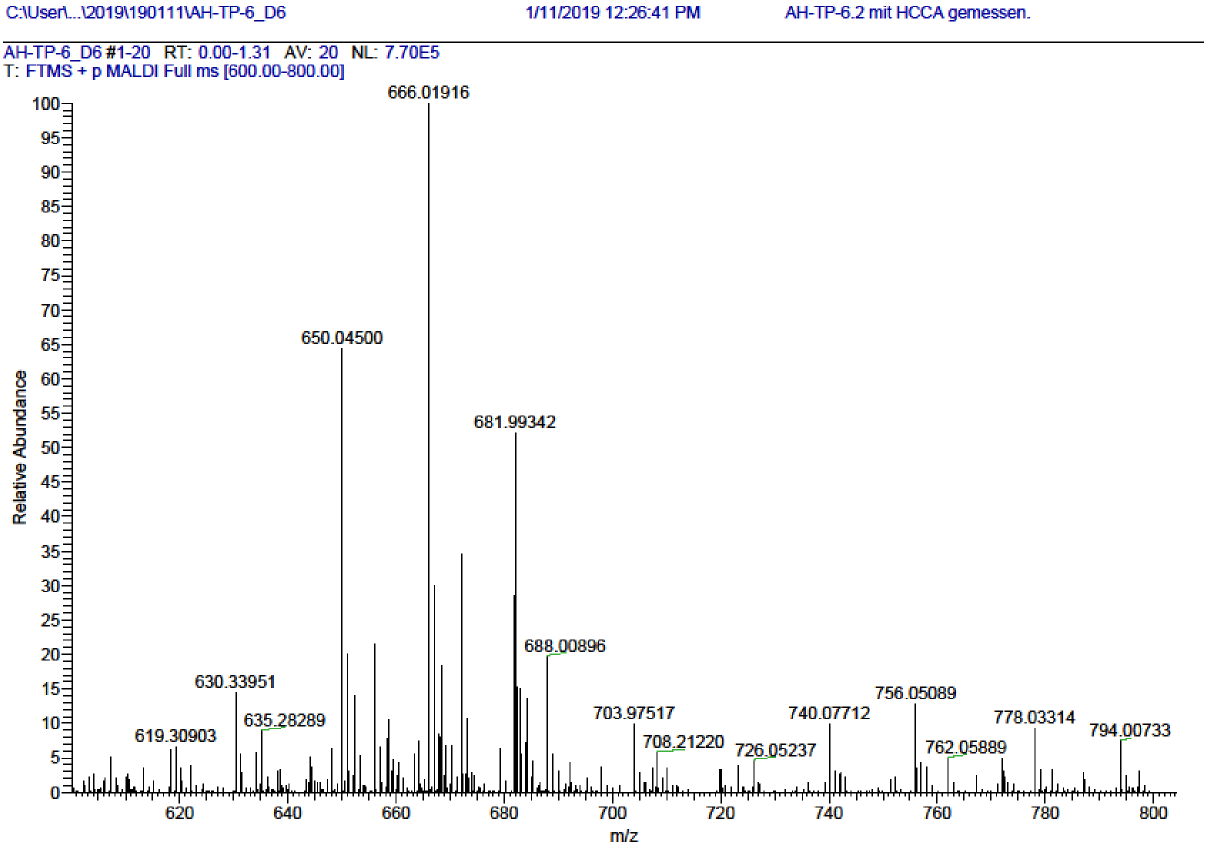

Supplement: Supplementary file 1 — Supplementary [file CHEM-26-1800-s001.pdf]
